# Hsa_circ_0058124 promotes papillary thyroid cancer tumorigenesis and invasiveness through the NOTCH3/GATAD2A axis

**DOI:** 10.1186/s13046-019-1321-x

**Published:** 2019-07-19

**Authors:** Yao Yao, Xinyuan Chen, Hong Yang, Wei Chen, Yichun Qian, Zhongyi Yan, Tian Liao, Weiping Yao, Wenlan Wu, Tonghua Yu, Yun Chen, Yuan Zhang

**Affiliations:** 1 Department of Head and Neck Surgery, Jiangsu Cancer Hospital & Jiangsu Institute of Cancer Research & The Affiliated Cancer Hospital of Nanjing Medical University, Nanjing, 210009 China; 2 Department of Immunology, Key Laboratory of Immune Microenvironment and Disease, Nanjing Medical University, Nanjing, 211166 China; 3 Jiangsu Key Lab of Cancer Biomarkers, Prevention and Treatment, Collaborative Innovation Center for Cancer Personalized Medicine, Nanjing Medical University, Nanjing, 211166 China; 4 Jiangsu Key Laboratory of Oral Disease, Nanjing Medical University, Jiangsu, 210029 China; 5 Department of Oral and Maxillofacial Surgery, Affiliated Hospital of Stomatology, Nanjing Medical University, Jiangsu, 210029 China; 6 Department of Head and Neck Surgery, Fudan University Shanghai Cancer Center, Shanghai, 200032 China; 7 Department of Oncology, Shanghai Medical College, Fudan University, Shanghai, 200032 China

**Keywords:** Papillary thyroid cancer, Circular RNA, Hsa_circ_0058124, NOTCH3, GATAD2A

## Abstract

**Background:**

Despite a good and overall prognosis, papillary thyroid cancer (PTC) can still affect the quality of life of many patients, and can even be life-threatening due to its invasiveness and metastasis. Emerging studies demonstrate that circular RNAs (circRNAs) participate in the regulation of various cancers. However, the circRNA profile in invasive PTC is still not well understood.

**Methods:**

Competing endogenous RNA (ceRNA) microarrays were performed to determine circRNAs contributed to the tumorigenesis and invasiveness of PTC. Bioinformatics methods were used to narrow down the candidate circRNAs. Quantitative real-time polymerase chain reaction (qRT-PCR) assays revealed a significant upregulation of hsa_circ_0058124 in PTC tissue and a close correlation with a poor prognosis for PTC patients. RNA fluorescence in situ hybridization and Cell fractionation assay were used to investigate the subcellular location of hsa_circ_0058124. Then, we examined the functions of hsa_circ_0058124 in PTC by cell proliferation, cell cycle, apoptosis, migration and invasion assay. Mechanistically, RNA sequencing and GSEA analysis were applied to predict the downstream pathway of hsa_circ_0058124. Dual-luciferase report assays were used to explore the potential miRNA sponge role of hsa_circ_0058124. Western blotting, cell proliferation, cell cycle, cell apoptosis, migration and invasion, and mouse xenograft assay were used to validate the effects of hsa_circ_0058124/NOTCH3/GATAD2A axis on PTC progression.

**Results:**

In the current study, a novel hsa_circ_0058124 on 2q35 was identified and explored in PTC. Hsa_circ_0058124 is associated with the malignant features and poor outcomes of PTC patients. Hsa_circ_0058124 acts as an oncogenic driver that promotes PTC cell proliferation, tumorigenicity, tumor invasion, and metastasis, which functions as a competing endogenous RNA to modulate miRNA-218-5p and its target gene NUMB expression, and consequently with repression of the NOTCH3/GATAD2A signaling axis in vitro and in vivo.

**Conclusions:**

This study unveils a novel biomarker panel consisting of the hsa_circ_0058124/NOTCH3/GATAD2A axis which is critical for PTC tumorigenesis and invasiveness and may represent a novel therapeutic target for intervening in PTC progression.

**Electronic supplementary material:**

The online version of this article (10.1186/s13046-019-1321-x) contains supplementary material, which is available to authorized users.

## Background

Thyroid cancer (TC) is the most prevalent endocrine malignancy in the world, and its incidence has ascended steadily over the past decades [1]. It occurs mostly in young women and is the fifth ranking cancer worldwide in women in terms of overall morbidity [2, 3]. Papillary thyroid cancer (PTC) is the most common histotype of TC, accounting for approximately 85% of all TC [1, 3, 4]. PTC has a generally good prognosis because of its steady growth rate and retention of iodide uptake. However, despite its overall good prognosis, PTC can still affect the quality of life of many patients and can even become life-threatening due to invasiveness and metastasis [5, 6]. Although the overall 5-year survival rate of PTC is 97%, patients with advanced PTC merely have a 5-year survival rate of only 59% [7]. Extrathyroidal extension (ETE) has long been recognized as an independent predictor of poor outcome in patients with PTC. Not only does such invasive disease result in higher rates of recurrence, but 5-year survival rate is compromised in this patient group [8–10]. This poor clinical prognosis of invasive PTC emphasizes the urgency of exploring the mechanism of invasive PTC and developing novel anticancer agents that could intervene in PTC progression.

Both genetic and environmental factors are crucial contributors to PTC, but the role of etiology is still not well understood [3]. However, many large-scale and multi-tissue sequencing programs employing next-generation sequencing technique and bioinformatics methods have been carried out in recent years. Annotation of the data derived from these experiments revealed that only 2% of the genes in the entire human genome are protein coding genes, while the majority are noncoding genes [11]. Among the noncoding transcripts, circular RNAs (circRNAs) represent a typical class generated by back-splicing and characterized by covalently closed loop structures that lack either 5′ to 3′ polarity and a polyadenylated tail. Accumulating studies have found that circRNAs are involved in various physiological and pathophysiological processes, for instance, modulating alternative splicing [12], sponging microRNA (miRNA) [13], and regulating protein-RNA interactions and genes expression [14]. CircRNAs have been detected in various eukaryotes and participate in the pathology of disease [15, 16]. Recent literature has also indicated dysregulated expression of several circRNAs in human cancer [17–19]. Nevertheless, little is known about the relationship between circRNAs and the tumorigenesis and invasiveness of PTC [20, 21]. Due to their closed structure, circRNAs have a high level of stability and resistance to RNA degradation. Therefore, they are likely to be promising and technically suitable biomarkers for PTC and can possibly function as molecular targets in PTC treatment.

In the present study, we detected the differential expression of hundreds of circRNAs in PTC tissues by competing endogenous RNA (ceRNA) microarrays. We then used a bioinformatics method to screen 13 circRNAs that might contribute to the tumorigenesis and invasiveness of PTC. Quantitative real-time polymerase chain reaction (qRT-PCR) assays revealed a significant upregulation of hsa_circ_0058124 in PTC tissue and a close correlation with a poor prognosis for PTC patients. These findings implied that hsa_circ_0058124 may be a novel oncogene associated with PTC tumorigenesis and invasiveness. We also found that hsa_circ_0058124 affects the proliferation, apoptosis, migration, and invasiveness abilities of PTC cells in vitro and in vivo. Mechanistically, we revealed that hsa_circ_0058124 may exert its biological effects in PTC in part through serving as a competing endogenous RNA to modulate miRNA-218-5p and its target gene NUMB expression, subsequently with repression of NOTCH3/GATAD2A axis. Taken together, the results revealed a novel biomarker panel consisting of the hsa_circ_0058124/NOTCH3/GATAD2A axis that is critical in PTC tumorigenesis and invasiveness and may be a novel therapeutic target to intervene PTC progression.

## Materials and methods

### Ethics statement and patient tissue samples

This study was approved by the Ethics Committee of Nanjing Medical University and was conducted in accordance with the government policies and the Declaration of Helsinki. PTC and paired adjacent normal tissues were obtained from 92 patients (Nanjing Medical University Affiliated Cancer Hospital, Nanjing) with a definite pathological diagnosis of PTC during operation. The adjacent normal tissues were collected 3 cm away from the PTC edge and were confirmed no tumor cells by two specialized pathologists. All samples were immediately frozen in liquid nitrogen after collection. After histopathological vetting, a total of 12 samples, consisting of eight PTC samples (including four invasive tumor tissues with extrathyroidal extension and metastasis and four non-invasive tumor tissues with no extrathyroidal extension or metastasis) and four matching adjacent normal tissues, were ultimately selected for ceRNA microarray analysis.

### RNA isolation and microarrays

The microarray experiments were performed by Shanghai Biotechnology Corporation (SBC) following the protocol of Agilent Technologies Inc. (CA, USA). Briefly, total RNA was isolated and purified using a mirVana™ miRNA Isolation Kit (Ambion, TX, USA), according to the manufacturer’s instructions. RNA samples from each group were then used to generate labeled complementary RNA (cRNA) targets using the Low Input Quick Amp WT Labeling Kit (Agilent Technologies, CA, USA) for the SBC human ceRNA microarray (4 × 180 K). The labeled cRNA targets were then hybridized with the slides and the slides were scanned on the Agilent Microarray Scanner (Agilent Technologies, CA, USA). Data were extracted with Feature Extraction software 10.7 (Agilent Technologies, CA, USA). CircRNAs with high expression abundances, expression levels with more than 2-fold alteration, and a *P* < 0.01 were selected for further analysis.

### RNA preparation and quantitative real-time PCR (qRT-PCR)

Total RNA extraction and quantification, RNA purification, and cDNA synthesis were conducted as described previously [22]. For RNase R treatment, 2 μg total RNA was incubated for 15 min at 37 °C with or without 3 U/μg RNase R (Epicentre Technologies, WI, USA). To detect RNA expression, quantitative real-time PCR (qRT-PCR) was performed with PowerUp™ SYBR™ Green Master Mix (Thermo Fisher, MA, USA) and the Applied Biosystems StepOnePlus™ Real-time PCR Detection System (Life Technologies, CA, USA). The relative gene expression was calculated using the 2^-ΔCT^ method normalized to GAPDH, and the fold change of gene expression was calculated by the 2^-ΔΔCT^ method. The primer sequences are shown in Additional file 1: Table S1. Bulge-loop™ miRNA qRT-PCR Primer Sets (one RT primer and a pair of qPCR primers for each set) specific for miR-218-5p was designed by RiboBio (Guangzhou, China). The relative expression of miR-218-5p was normalized to human U6 snRNA.

### Cell culture and transfection

TPC-1 and K1 cells were purchased from University of Colorado Cancer Center Cell Bank. These PTC cell lines were maintained in a humidified incubator (95% air/5% CO_2_) at 37 °C in 90% RPMI 1640 (Invitrogen, CA, USA) supplemented with 10% fetal bovine serum (FBS; Invitrogen, CA, USA), 100 U/ml penicillin, and 100 μg/mL streptomycin. The cells were passaged at a 1:3 ratio in 10 cm dishes once 90% confluence was reached. All PTC cell lines used in this study were reported previously [21, 23]. Purchased siRNAs (RealGene, Nanjing, China) were used in transfection experiments. The TPC-1 and K1 cells were cultured to 50–60% confluence, and the transfection was carried out with Lipofectamine 3000 (Invitrogen, CA, USA). Sequences of the siRNAs are shown in Additional file 1: Table S2. To construct a plasmid expressing NUMB, the full-length human NUMB sequence (NCBI Reference Sequence: NM_001005745.1) was synthesized and cloned into the pCDNA3.1 vector (RealGene, Nanjing, China).

### Fluorescent in situ hybridization (FISH)

Hsa_circ_0058124 FISH staining was performed according to the manufacturer’s instructions. Briefly, cells were washed with a solution of 0.5% Triton X-100 in 1 × PBS, an appropriate amount of anti-HNF1A-AS1 oligodeoxy-nucleotide probe (RiboBio, Guangzhou, China) was added in a hybridization solution containing 1% blocking solution, and hybridization was allowed to occur overnight in a humid chamber at 37 °C. The cells were then given three 5-min washes in the dark in 4× sodium citrate buffer (SSC), 2× SSC, and 1× SSC containing 0.1% Tween-20 at 42 °C. The cells were then given one 5-minwash in phosphate buffered saline (PBS) at room temperature and then stained with 4′,6-diamidino-2-phenylindole (DAPI). The cells were then given three 5-min washes with 1× PBS before imaging by confocal laser scanning microscopy (Zeiss LSM 710 confocal microscope**,** Germany) to detect the location of hsa_circ_0058124 in the PTC cells.

### Cell fractionation assay

Cytoplasmic and nuclear RNA was acquired using a Cytoplasmic and Nuclear RNA Purification Kit (Invitrogen, CA, USA). Briefly, the cells were harvested and incubated for 10 min with lysis solution on ice. The cells were then centrifuged for 3 min at 12000 *g*. The supernatant was collected for cytoplasmic RNA and the nuclear pellet was used for nuclear RNA extraction. GAPDH was used as the cytoplasmic endogenous control. The U6 small nuclear RNA was used as the nuclear endogenous control.

### Cell viability assay

Cells were seeded at a density of 2 × 10^3^ cells per well (100 μL) in a 96-well plate. Cell viability was evaluated with the cell counting kit-8 (CCK-8; Bimake, Shanghai, China) after 0, 24, 48, 72, and 96 h of transfection. The absorbance was determined with a BioTek Synergy HTX multi-mode reader at 450 nm.

### Colony formation assay

Crystal violet (KeyGen, China) was used to detect colony formation. About 200 cells were seeded in each well of 6-well plates. After transfection, the growth medium was changed every 3 days. After 9 days, the cells were fixed with 75% ethanol for 2 h, followed by staining with 0.2% crystal violet for 2 h, and colonies (> 50 cells/colony) were counted and photographed. Each experiment was performed three times independently.

### 5-ethynyl-2′-deoxyuridine (EdU) assay

EdU assays were performed using the Cell Light™ EdU kit (RiboBio, Guangzhou, China). Briefly, 6 × 10^4^ cells were seeded in well apiece of a 24-well plate. After transfection for 48 h, the culture medium was supplemented with 50 μM EdU for another 2 h. The cells were fixed with 4% formaldehyde for 30 min and incubated with glycine (2 mg/mL) for 10 min. The cells were then permeabilized with 0.5% Triton X-100 for 20 min, and then Hoechst 33342 were added. Cell proliferation (the percentage of EdU positive cells) was then determined by fluorescence microscopy (Olympus IX73, Japan). Assays were performed independently three times in triplicate.

### Cell cycle analysis and apoptosis assay

After transfection for 48 h, the cells were collected for cell cycle and apoptosis assays. The apoptosis assay was performed with the Apoptosis Detection Kit (BD Pharmingen, CA, USA) by suspending the cells in 100 μL binding buffer containing 5 μL PE and 5 μL 7-ADD, incubating them in the dark for 15 min, and adding 400 μL binding buffer to resuspend the cells. The cells were divided into viable cells, dead cells, early apoptotic cells, and late apoptotic cells. The early and late apoptotic cells were measured. For cell cycle analysis, the cells were fixed in 75% ethanol overnight at − 20 °C, then stained with propidium iodide (PI) for 30 min, away from light. The cells were analyzed by flow cytometry (FACS Calibur Flow Cytometer, BD Biosciences, USA) to quantify the cell cycle or cell apoptosis. All experiments were performed independently in triplicate.

### Cell migration and invasion assay

Transwell migration and invasion abilities were assessed using 8 μm pore transwell chambers either without Matrigel (for migration assays) or with Matrigel (for invasion assays). Each well of the upper chamber was filled with 300 μL of serum-free RPMI 1640 medium containing 1 × 10^5^/mL infected cells. The lower chambers contained 700 μL complete medium. The cells were fixed in 4% paraformaldehyde and stained with crystal violet after a 48-h incubation. The cells remaining in the top chamber were removed with cotton swabs, and imaged by light microscopy (Olympus, China). All assays were performed three times independently using triplicate wells.

### Scratch wound assay

Scratch wound assays were conducted by measuring the widths of the scratches at different times. Each well of 6-well plates was plated with 3 × 10^5^ cells. After 48 h incubation, when the well was completely covered with cells, a straight scratch was made with a 200 μl pipette tip. Images of the same fields were taken at different time points (0, 12, 24, 30, 36, and 40 h) after the scratch. Images at different time points were used to create an animation sequence in accordance with the timeline. The experiment was repeated at least three times independently.

### RNA sequencing

We transiently transfected 3 × 10^6^ TPC-1 cells with 100 nM indicated siRNAs for 48 h, and the total RNA samples were collected using TRIzol reagent for whole-genome transcriptome profiling by RNA sequencing. The RNA sequencing and bioinformatics data analysis were performed by Shanghai Biotechnology Corporation. In brief, total RNA was extracted using the RNeasy Micro Kit (Qiagen, Hilden, Germany) and purified with the RNAClean XP Kit (Beckman Coulter, Inc., CA, USA). Paired-end libraries were then synthesized using the TruSeq® RNA Sample Preparation Kit (Illumina, USA) following the TruSeq® RNA Sample Preparation Guide. The purified libraries were sequenced on the Illumina HiSeq X-ten (Illumina, USA). For data processing, we used Hisat2 (version:2.0.4) [24] to map the cleaned reads to the human GRCh38 reference genome with two mismatches. After genome mapping, Stringtie (version:1.3.0) [25] was run with a reference annotation to generate FPKM (Fragments Per Kilobase of transcript per Million mapped reads) values for known gene models. The *P*-value and FDR were used to define the threshold of significance.

### KEGG analysis and gene set enrichment analysis (GSEA)

To gain insight into hsa_circ_0058124-mediated molecular pathways in PTC, we used the Kyoto Encyclopedia of Genes and Genomes (KEGG) pathway analysis (https://www.kegg.jp). GSEA was also performed using the Broad Institute GSEA version 3.0 software. The gene sets used for the enrichment analysis were downloaded from the Molecular Signatures Database (MsigDB, http://software.broadinstitute.org/gsea/index.jsp).

### Western blotting

The proteins in PTC cells were extracted using the Total Protein Extraction Kit (Thermo Fisher, MA, USA). The protein extracts (30–40 μg) were separated on 12% sodium dodecyl sulfate polyacrylamide gel electrophoresis gels and then electrophoretically transferred to polyvinylidene difluoride membranes (Millipore, USA). After blocking in 5% non-fat milk for 2 h, the membranes were incubated overnight at 4 °C with primary antibodies recognizing N3ICD, HES1 (1:1000 dilution; Cell Signaling Technology, USA), NUMB (1:1000 dilution; Abcam, UK), GATAD2A (1:1000; Proteintech, USA), and GAPDH (1:1000 dilution; Proteintech, USA). After incubation with secondary antibodies (1:5000 dilution; Jackson ImmunoResearch, PA, USA), the protein bands were visualized by chemiluminescence using a GE Amersham Imager 600 (GE, USA).

### Dual-luciferase reporter assay

To construct luciferase reporter vectors, the full length of hsa_circ_0058124 or the 3’UTR fragment of NUMB (NM_001005745.1; 3’UTR: 2104–3467) containing the predicted potential binding sites were cloned at the XhoI and NotI sites into the pmiR-RB-REPORT™ luciferase reporter vector (RiboBio., Guangzhou, China). The hsa_circ_0058124 mutant sequence or the 3’UTR mutant sequence of NUMB, in which seven nucleotides were mutated within the miR-218-5p binding sites, were also constructed into the vector.

For luciferase activity assay, each construct was cotransfected with indicated miRNAs (RiboBio) in 293 T cells using Lipofectamine 3000 (Invitrogen) for 48 h. Luciferase assays were performed with the Dual-Luciferase Reporter Assay System (Promega, WI, USA) according to the manufacturer’s instructions. Luminescent signal was quantified by BioTek Synergy HTX multi-mode reader, and luciferase activity was presented by relative hRluc/ hluc ratio.

### Immunohistochemistry (IHC) analysis

All tissues were fixed overnight in a formalin solution, dehydrated in ethanol, embedded in paraffin, and sectioned at 5 μm. Then, the slides were treated with xylene and ethanol to remove the paraffin. The slides were blocked with 5% normal goat serum and incubated with anti-GATAD2A overnight at 4 °C. After washing with PBS, the slides were incubated with goat anti-rabbit horseradish peroxidase (Jackson ImmunoResearch, PA, USA) for 1 h at room temperature, and was detected the immunohistochemical reactions using a DAB kit (Vector Laboratories, CA, USA). The slides were examined under a phase contrast light microscope (Olympus IX73, Japan), and the average integral optical density of each positively stained slide was measured using an Image-Pro plus 6.0 image analysis system. Three areas were chosen randomly from each section for measurement.

For validation of differentially expressed genes, the IHC data of thyroid cancer tissues and normal thyroid tissues were downloaded from Human Protein Atlas [26] available from www.proteinatlas.org. Protein expression levels were scaled based on an overall antibody staining score ranging from not detected to high.

### Animal studies

All animal experiments were performed in accordance with a protocol approved by the Institutional Animal Care and Use Committee of Nanjing Medical University. Nude mice (5 in each group) aged 5–6 weeks were injected subcutaneously with 0.1 ml of a cell suspension containing 3.0 × 10^6^ TPC-1 cells for the control group, the LV-sh-hsa_circ_0058124 group, the LV-sh-NOTCH3 group, the LV-sh-GATAD2A group, the LV-sh-hsa_circ_0058124 together with LV-sh-NOTCH3 group or the LV-sh-hsa_circ_0058124 together with LV-sh-GATAD2A group (Genechem, Shanghai, China) in the right flank. After the tumor was palpable, it was measured every 3 days. Its volume was calculated according to the formula (volume = length × width^2^ × 0.5).

### Statistical analysis

Data analysis was handled with SPSS ver. 19.0 (SPSS, Chicago, IL, USA). All experimental data were expressed as the mean ± S.E.M. For continuous variables were analyzed with a two-sided independent t-test or paired t-test when data assumed to have an equal variance per test; otherwise, the Wilcoxon signed-rank test was used to perform the comparisons. Pearson’s chi-squared test was used for comparisons of categorical variables. In this study we considered *P* < 0.05 as statistically significant.

## Results

### CircRNA and mRNA profiling in human PTC tissues and hsa_circ_0058124 characterization

A total of twelve fresh PTC tissue samples, decribed in the previous paragraph, were selected for ceRNA microarray analysis. Detailed information of patient characteristics is provided in Additional file 1: Table S3.

**Fig. 1:**
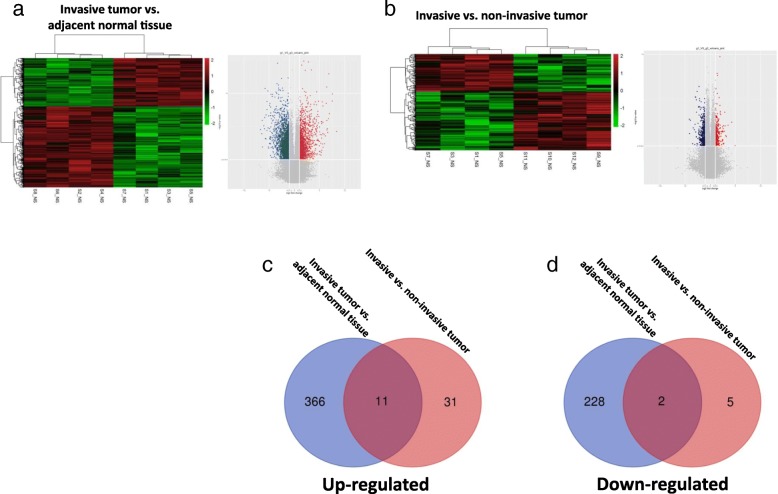
Screening of PTC-related circRNAs using multiple microarrays and bioinformatics. Hierarchical clustering analysis and volcano plots of significantly differentially expressed circRNAs in the invasive tumor vs. adjacent normal tissue set (**a**) and the invasive vs. non-invasive tumor set (**b**). **c**, **d** The Venn diagrams demonstrated the significantly changed circRNAs that overlapped in the foregoing comparison in PTC

The circRNA expression in the PTC samples was profiled using a 4 × 180 K ceRNA microarray (circRNA, lncRNA, and mRNA). The differentially expressed circRNAs were identified by fold-change filtering (|fold change| > 2) and the Student’s t-test (*P*-value < 0.01), which revealed 607 circRNAs that were significantly differentially expressed in the invasive tumor vs. adjacent normal tissue set (Fig. 1a) and 49 circRNAs that were significantly differentially expressed in invasive vs. non-invasive tumor set (Fig. 1b). Hierarchical clustering was then performed to demonstrate the circRNA expression patterns among the sets (Fig. 1a, b).

**Fig. 2:**
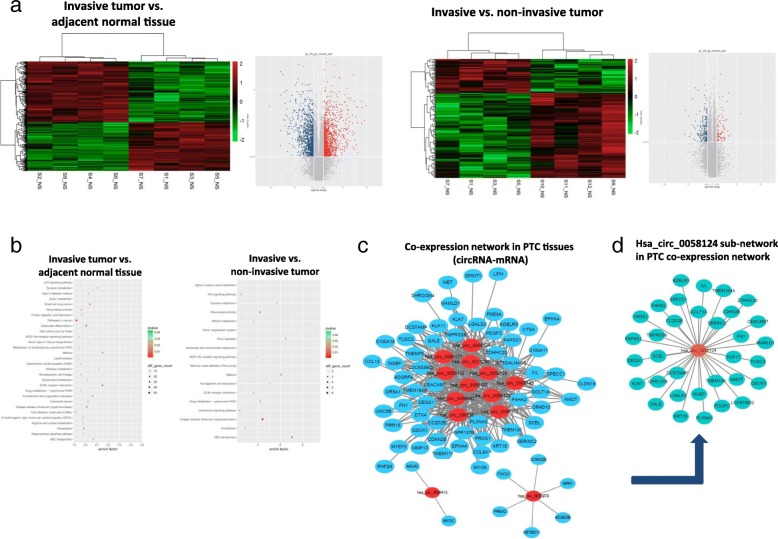
The mRNA profiling and coexpression network of circRNA-mRNA interactions in PTC. **a**, **b** Hierarchical clustering analysis and KEGG analysis of the mRNA expression profile in tumor vs. non-tumor set and in invasive vs. non-invasive tumor set. **c** The coexpression network map included the 13 identified significantly changed circRNAs (represented as red nodes). The blue nodes around the red nodes are the predicted mRNAs that interacted with the indicated circRNAs in the eight PTC tumor samples. **d** The coexpression network for hsa_circ_0058124

To investigate the contribution of circRNAs to the tumorigenesis and invasiveness of PTC, we calculated the intersection of differential expressions of circRNA between the invasive vs. non-invasive tumor set and the invasive tumor vs. adjacent normal tissue set. This narrowed the number of candidate circRNAs down to 13. Figure 1c and d showed the Venn diagrams for this intersection and identified 11 significantly upregulated circRNAs and 2 downregulated circRNAs that overlapped the results of the previous comparisons (Additional file 1: Table S4). The mRNA expression profiles among the sets were also demonstrated using hierarchical clustering analysis and Kyoto Encyclopedia of Genes and Genomes (KEGG) pathway enrichment analysis (Fig. 2a, b). To investigate the potential target gene of the identified 13 circRNAs, a coexpression network analysis was used to screen these circRNAs in the eight PTC tumor samples (Fig. 2c). We also randomly selected 6 target genes to validate this circRNA-mRNA coexpression by qRT-PCR in 20 PTC tissues (Additional file 2: Figure S1). We then experimentally validated these 13 circRNA expression levels by qRT-PCR in larger-scale PTC tissue samples. The qRT-PCR results indicated hsa_circ_0058124 showed highest fold-change in the PTC tissues than in the control tissues, and was significantly upregulated in the invasive PTC tissues compared with non-invasive tumor tissues (Additional file 2: Figure S2). We therefore chose hsa_circ_0058124 for further analysis. The coexpression network for hsa_circ_0058124 was also shown in Fig. 2d.

**Fig. 3:**
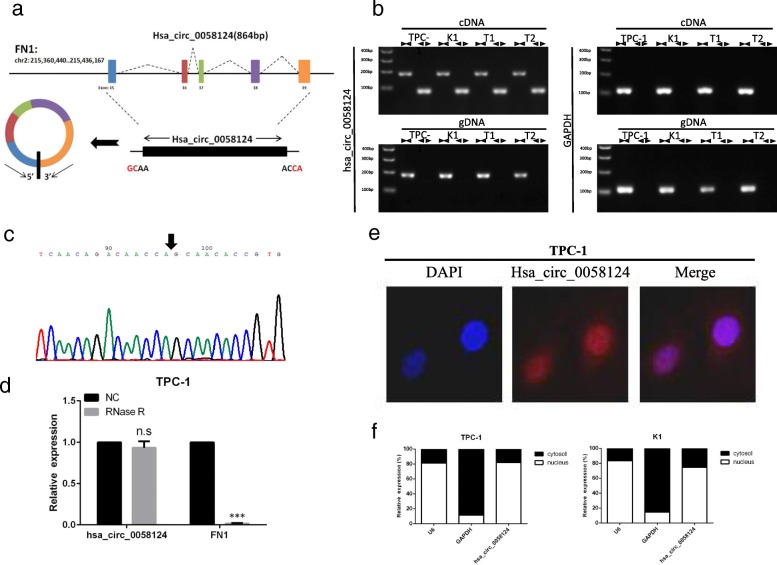
Characterization of hsa_circ_0058124 in PTC cells. **a** Schematic diagram of the genomic location and splicing pattern of hsa_circ_0058124 (864 bp). **b** The existence of hsa_circ_0058124 was validated by RT-PCR in two PTC tumor tissue samples (T1 and T2) and two PTC cell lines (TPC-1 and K1). **c** Arrow represents the “head-to-tail” splicing sites of hsa_circ_0058124 according to Sanger sequencing. **d** qRT-PCR was used to determine the abundance of hsa_circ_0058124 and linear FN1 mRNA in TPC-1 cells treated with RNase R (normalized to negative control). **e** RNA fluorescence in situ hybridization (FISH) showed the localization of hsa_circ_0058124 in TPC-1 cells. DAPI solution was used to stain the nuclei. **f** qRT-PCR data indicating the abundance of hsa_circ_0058124 in either the cytoplasm or nucleus of TPC-1 and K1 cells. The data represent the mean ± S.E.M. from three independent experiments. ^*^P < 0.05; ^**^P < 0.01

Hsa_circ_0058124 is located on chromosome 2, is 864 base pairs (bp) in length, and consists of 5 exons (exons 15–19) from its host gene Fibronectin 1 (FN1) genome (Fig. 3a; Additional file 3). FN1, a fundamental component of the extracellular matrix, has been considered as a biomarker of PTC, and an important modulator of thyroid cancer aggressiveness [10, 27]. We avoided trans-splicing or genomic rearrangements, including head-to-tail splicing, by employing several universal circRNA detection methods [28]. We first designed convergent primers to amplify FN1 mRNA (a 184-bp fragment) and divergent primers to amplify hsa_circ_0058124 (a 83-bp fragment). We used cDNA and genomic DNA from two randomly selected PTC tissue and two PTC cell lines (TPC-1 and K1) as templates to amplify hsa_circ_0058124 from cDNA using only divergent primers (an expected 83-bp fragment), while no amplification product was observed from genomic DNA (Fig. 3b). We also used Sanger sequencing to confirm the head-to-tail splicing in the RT-PCR product of hsa_circ_0058124 with the expected size (Fig. 3c). RNase R is an exoribonuclease that can degrade RNA from its 3′ to 5′ end but does not act on circRNA [29]. In contrast to the linear FN1 mRNA, hsa_circ_0058124 was resistant to RNase R (Fig. 3d). The cell fraction assay and FISH analysis against hsa_circ_0058124 showed that hsa_circ_0058124 was primarily located in the nucleus and also existed in cytoplasm (Fig. 3e-f). Our results implied that hsa_circ_0058124 is a bona fide exonic circRNA that is abundant and stable in TPC-1 cells.

### Upregulation of hsa_circ_0058124 is correlated with unfavorable prognosis in PTC

**Fig. 4:**
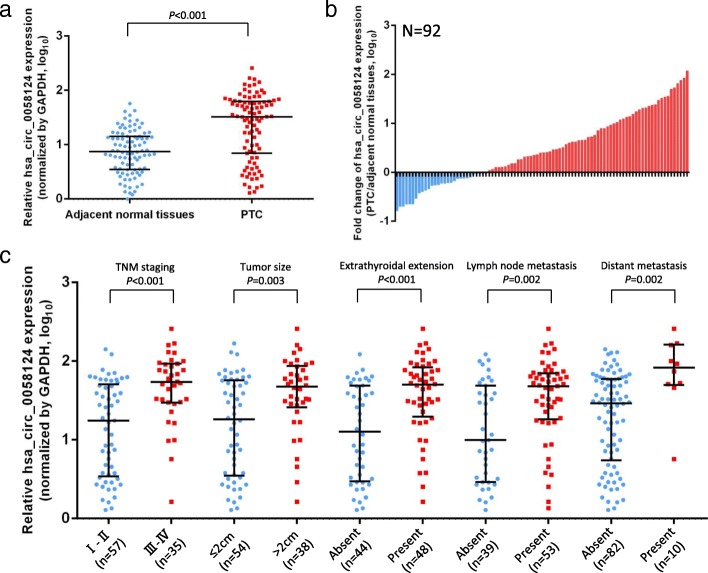
Hsa_circ_0058124 is associated with clinicopathological characteristics in patients with PTC. **a** The copy numbers of hsa_circ_0058124 were determined by qRT-PCR in 92 pairs of PTC tissues and adjacent normal tissues. **b** Fold change of hsa_circ_0058124 copy number variations in 92 paired tissues. **c** Clinical significance of hsa_circ_0058124 in patients with PTC; high hsa_circ_0058124 expression was positively correlated with advanced stage, tumor size, extrathyroidal extension, lymph node metastasis, and distant metastasis. Values are expressed as the median with interquartile range in (**a**, **c**)

Quantitative real-time PCR analysis was conducted to detect hsa_circ_0058124 expression patterns in 92 paired PTC tissue samples. The hsa_circ_0058124 expression was significantly upregulated in most tumor tissues (63/92, 68.48%) when compared with the adjacent normal counterparts (Fig. 4a, b). We also investigated the clinical significance of hsa_circ_0058124 by analyzing the correlations between its expression level and clinicopathological characteristics. The median value of the relative expression of hsa_circ_0058124 in PTC tissues was 1.514, and this was used as a cut-off value to divide the patients into two groups: a high hsa_circ_0058124 relative expression (≥1.514; *n* = 46) and a low hsa_circ_0058124 relative expression (< 1.514; *n* = 46). The clinicopathological characteristics of the 92 PTC patients are shown in Table 1 and Fig. 4. The analysis results showed that hsa_circ_0058124 expression level was significantly higher in patients with large tumor size, advanced stage, extrathyroidal extension, lymph node metastasis, or distant metastasis (Table 1, Fig. 4c). No significant correlation was noted between hsa_circ_0058124 expression and age, gender, or multifocality (*p* > 0.05; Table 1). These results suggest that hsa_circ_0058124 was upregulated in PTC and correlated with unfavorable prognosis. Hsa_circ_0058124 might therefore be considered as a novel prognostic biomarker.

**Table 1:** Correlation between hsa_circ_0058124 expression and clinicopathological features of 92 PTC patients

Characteristics	*N*	Relative hsa_circ_0058124 expression		*P* value^†^
		High	Low	
Age (years)				0.210
<55	44	19	25	
≥ 55	48	27	21	
Gender				0.402
male	42	23	19	
female	50	23	27	
TNM staging				0.005^*^
I-II	57	22	35	
III-IV	35	24	11	
Tumor size				0.034^*^
≤ 2 cm	54	22	32	
>2 cm	38	24	14	
Extrathyroidal extension				
Present	48	29	19	0.037^*^
Absent	44	17	27	
Multifocality				
Present	25	14	11	0.482
Absent	67	32	35	
Lymph node metastasis				0.020^*^
Present	53	32	21	
Absent	39	14	25	
Distant metastasis				0.007^*^
Present	10	9	1	
Absent	82	37	45	

^†^Pearson’s chi-squared test

^*^P < 0.05

### Hsa_circ_0058124 loss of function dramatically impairs the PTC cell malignant phenotypes in vitro

**Fig. 5:**
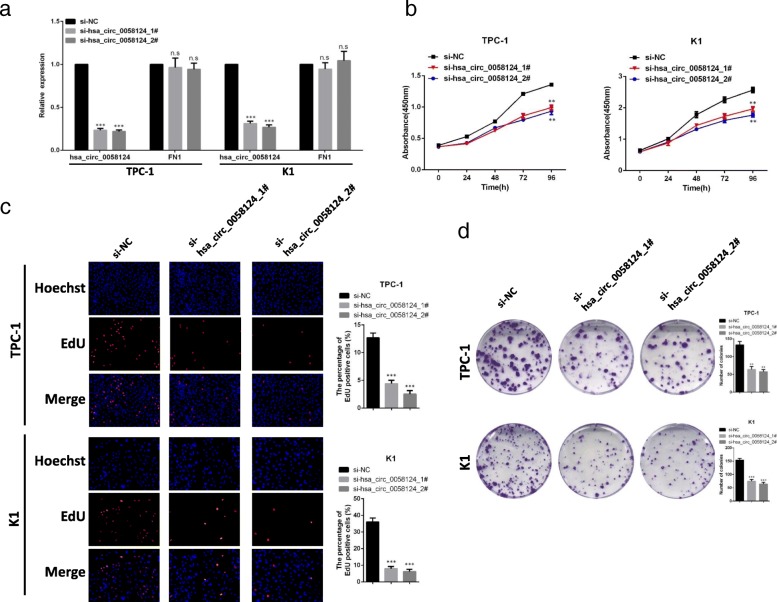
Hsa_circ_0058124 loss of function impairs PTC cell proliferation in vitro. **a** qRT-PCR analysis of hsa_circ_0058124 and its host gene FN1 relative expression in TPC-1 and K1 cells transfected with two specific hsa_circ_0058124 siRNAs. **b**-**d** CCK-8 assays (**b**), EdU assays (**c**), and colony formation assays (**d**) were performed to determine cell proliferation ability in TPC-1 and K1 cells transfected with si-NC or si-hsa_circ_0058124. ^*^P < 0.05, ^**^P < 0.01 and ^***^P < 0.001

**Fig. 6:**
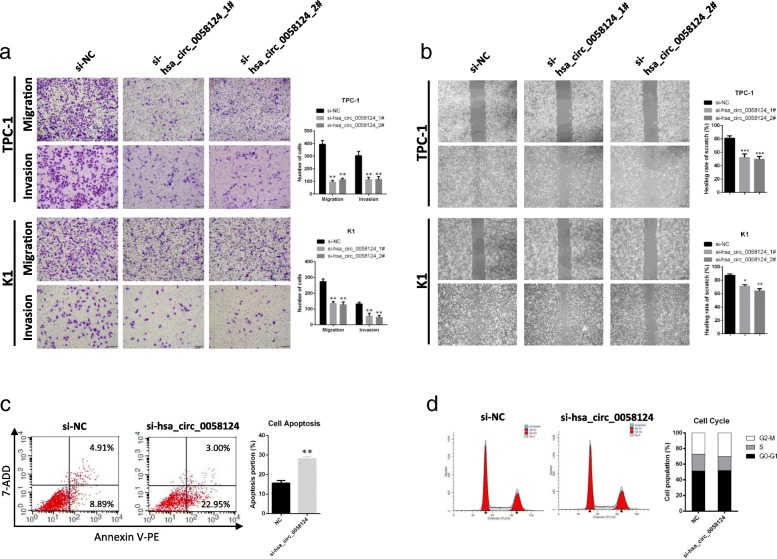
Hsa_circ_0058124 affects the migration, invasion abilities, and apoptosis of PTC cells in vitro. **a** Representative images and quantification results of cell migration and invasion abilities of TPC-1 and K1 cells harboring control or hsa_circ_0058124 siRNAs. **b** Scratch wound assays in hsa_circ_0058124-deficient PTC cells and corresponding controls. **c**, **d** Flow cytometry assays showed the rate of apoptosis (**c**) and the cell cycle distributions (**d**) in TPC-1 cells transfected with si-NC or si-hsa_circ_0058124. ^*^P < 0.05, ^**^P < 0.01 and ^***^P < 0.001

We further studied the functional role of hsa_circ_0058124 in PTC cell lines by designing two siRNA oligonucleotides that would target the unique backsplice junction. These backsplice junction-specific siRNAs successfully decreased hsa_circ_0058124 expression but did not affect the linear FN1 mRNA level in TPC-1 and K1 cells (Fig. 5a). We then observed that hsa_circ_0058124 knockdown significantly decreased the cell viability determined by CCK-8 assays (Fig. 5b). Similar results were obtained with the EdU assays, as the percentage of EdU positive cells was significantly decreased when compared with the control group (Fig. 5c). Similarly, colony formation assays revealed that hsa_circ_0058124 knockdown greatly attenuated the numbers of visible colonies (Fig. 5d). Transwell migration and invasion assays demonstrated that hsa_circ_0058124 knockdown impaired the migration and invasion ability (Fig. 6a), and the wound-healing assays showed that hsa_circ_0058124 silencing significantly suppressed the migration (Fig. 6b). Further flow cytometry investigations of the effects of hsa_circ_0058124 on cell cycle and apoptosis revealed the percentages of apoptotic cells significantly increased after hsa_circ_0058124 knockdown in TPC-1 cells (Fig. 6c), while the proportions of cells in each cell cycle phase were not significantly different between the si-hsa_circ_0058124 group and the control group (Fig. 6d).

### The NOTCH3 signaling pathway is a functional downstream pathway of hsa_circ_0058124

**Fig. 7:**
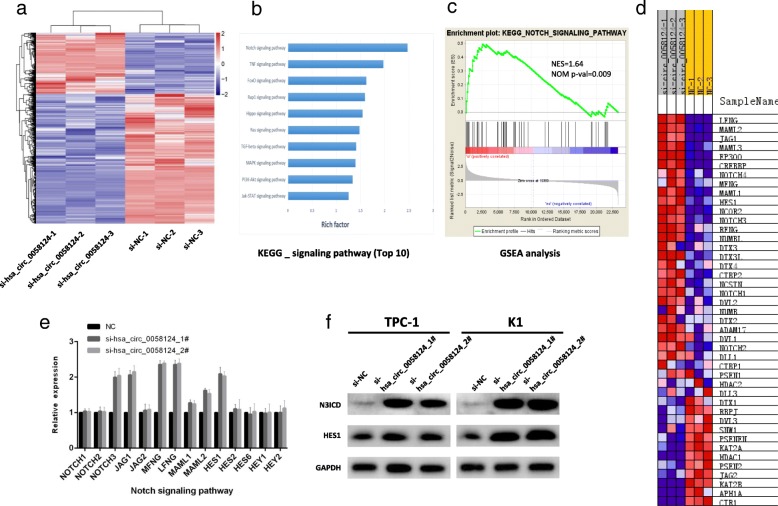
Hsa_circ_0058124 promotes PTC progression through NOTCH3 signaling pathway. **a** Heatmap representation of the significantly differentially expressed genes from RNA-seq data after treatment with the indicated siRNAs. **b** KEGG enrichment focused on a set of signaling pathways after hsa_circ_0058124 silencing, summarized based on the enrichment score. **c**, **d** GSEA analysis of the “KEGG_NOTCH_SIGNALING_PATHWAY” gene set in the si-NC versus si-hsa_circ_0058124 group in TPC-1 cells. The top portion of the figure plots the enrichment scores for each gene, whereas the bottom portion of the plot shows the value of the ranking metric moving down the list of ranked genes. Y-axis: value of the ranking metric; X-axis: the rank for all genes. NES, normalized enrichment score. **e** The mRNA levels of the related genes in the NOTCH signaling pathways in TPC-1 cells treated with si-hsa_circ_0058124. **f** NOTCH3 and HES1 protein levels in TPC-1 and K1 cells with hsa_circ_0058124 deficiency. Values are expressed as the mean ± S.E.M, *n* = 3 in a, b, c, d. ^*^P < 0.05, ^**^P < 0.01 and ^***^P < 0.001

We investigated the potential functional signaling pathways related to hsa_circ_0058124 in PTC using unbiased transcriptome profiling by RNA sequencing in TPC-1 cells transfected with si-hsa_circ_0058124. The depletion of hsa_circ_0058124 affected the expression levels of 457 genes: 239 genes were upregulated and 218 genes were downregulated (q-value < 0.05, |fold change| > 2, Fig. 7a). The KEGG pathway enrichment analysis showed that hsa_circ_0058124 affected several downstream signaling pathways involved in cell proliferation and metastasis, including the Notch, TNF, and FoxO signaling pathways (Fig. 7b). Since the Notch signaling pathway ranked first above all signaling pathways and plays an essential role in cell migration and invasion [30], we investigated this signaling pathway as a potential functional downstream pathway for hsa_circ_0058124. GSEA analysis showed that the gene set of KEGG_NOTCH_SIGNALING_PATHWAY was significantly enriched in si-hsa_circ_0058124 group compared to si-NC group, indicating that this gene set could be significantly associated with hsa_circ_0058124 expression in PTC (Fig. 7c-d). We further validated the related genes in the NOTCH signaling pathways and found that the NOTCH3/HES1 axis showed the most significant differential expression in the pathway [NOTCH4 and MAML3 were excluded because of too low expression (CT value>30)] (Fig. 7e). We also verified the NOTCH3 and HES1 protein levels by western blotting and confirmed that NOTCH3 pathway dramatically negatively related to hsa_circ_0058124 (Fig. 7f). Taken together, these findings demonstrated that hsa_circ_0058124 is an oncogenic driver that function by down-regulating the NOTCH3 signaling pathway in PTC.

### Hsa_circ_0058124 affects the NOTCH3 pathway via miR-218-5p/NUMB

**Fig. 8:**
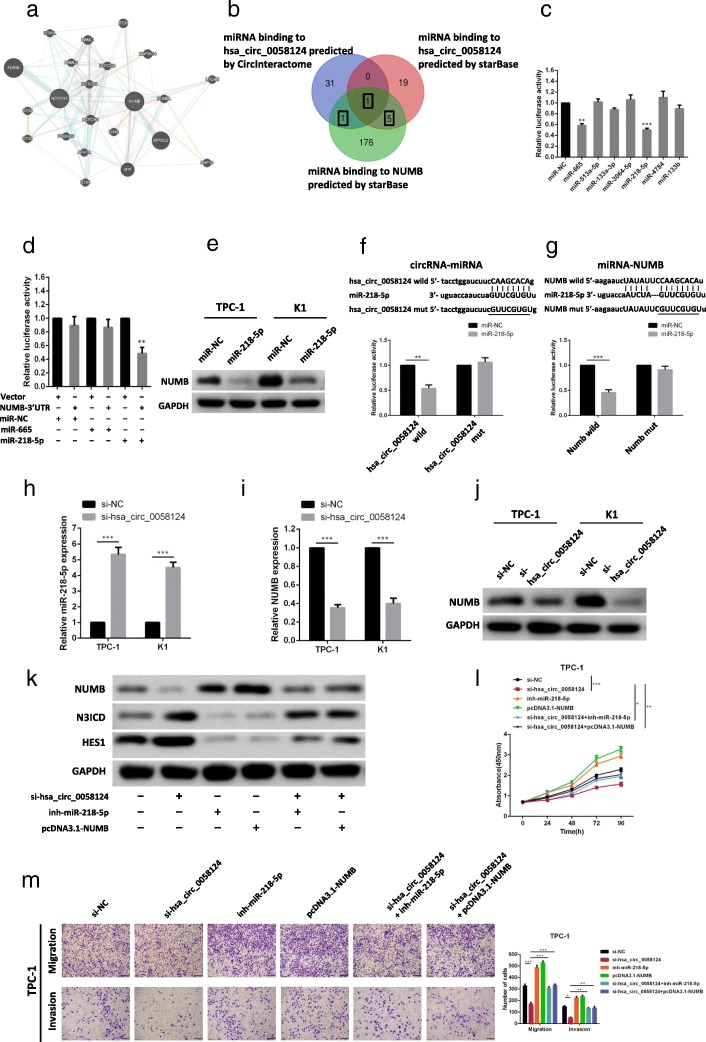
Hsa_circ_0058124 affects the NOTCH3 pathway via miR-218-5p/NUMB. **a** The interaction network between the NOTCH3 pathway and hsa_circ_0058124 using GeneMANIA web tool [31]. **b** Venn diagram showing the mutual putative target genes of hsa_circ_0058124 and NUMB predicted by CircInteractome and starBase. **c** Dual-luciferase assays showing the luciferase activity of the pmiR-RB-Report-hsa_circ_0058124 vector in 293 T cells cotransfected with indicated miRNA mimics. **d** Dual-luciferase assays confirmed the interaction of NUMB with miR-218-5p. **e** NUMB protein level after transfection with miR-NC or miR-218-5p. **f**, **g** mutant (mut) and wild-type (wt) luciferase reporter constructs of hsa_circ_0058124 (**f**) or NUMB (**g**) were transfected into TPC-1 cells together with miR-218-5p mimics or controls (miR-NC). Dual-luciferase assays were conducted to detect relative luciferase activity. **h** Expression level of miR-218-5p in TPC-1 and K1 cells infected with si-hsa_circ_0058124 or negative control (si-NC) were measured by qRT-PCR. **i-j** NUMB mRNA (**i**) and protein level (**j**) in TPC-1 and K1 cells transfected with si-hsa_circ_0058124 or si-NC. **k** Western blot assays were performed to measure the NOTCH3 and HES1 protein level in TPC-1 cells with indicated treatments. **l-m** CCK-8 assays (**l**) and transwell assays (**m**) in TPC-1 cells with indicated treatments

Given that circRNAs have been reported to function as miRNA sponges, we speculated that hsa_circ_0058124 might regulate the NOTCH3 pathway by sponging miRNAs. Western blottings have indicated that the NOTCH3 pathway is significantly downregulated by hsa_circ_0058124, and NUMB was reported to be a strong suppressor of the NOTCH pathway (Fig. 8a) [32, 33], we hypothesized that hsa_circ_0058124 may upregulate NUMB by sponging miRNA, which resulted in the NOTCH3 pathway suppression. Two powerful web tools, CircInteractome [34] and starBase [35], were employed to predict the potential miRNA binding partners of hsa_circ_0058124 and NUMB. As shown in Fig. 8b, we further performed Venn diagram to intersect the predicted target miRNAs and narrowed the number of miRNAs down to 7 (miR-665, miR-513a-5p, miR-133a-3p, miR-3064-5p, miR-218-5p, miR-4784, miR-133b). Dual luciferase assays were conducted to validate whether these miRNAs could directly target hsa_circ_0058124 and NUMB. Luciferase reporters were cotransfected with each miRNA mimic into 293 T cells. We found that 2 miRNAs (miR-665, miR-218-5p) significantly inhibited the luciferase reporter activity (Fig. 8c), suggesting that hsa_circ_0058124 could bind to these miRNAs. The pmiR-RB-Report vector containing 3′-UTR regions of NUMB cotransfected with miR-218-5p mimic resulted in a remarkable decrease in the Renilla/firefly ratio compared with the control vector, but not in the NUMB 3′-UTR vector cotrasfected with miR-665 (Fig. 8d), suggesting that miR-218-5p could directly bind to NUMB. Concordantly, the protein level of NUMB was significantly reduced in cells transfected with miR-218-5p mimic when compared with control cells (Fig. 8e). The results above indicated that miR-218-5p could bind both hsa_circ_0058124 and NUMB directly, and modulated NUMB expression. To further verify the miR-218-5p binding sites in hsa_circ_0058124 and NUMB, mutant (mut) and wild-type (wt) luciferase reporter constructs were transfected into TPC-1 cells together with miR-218-5p mimics or controls (miR-NC). miR-218-5p mimics significantly inhibited luciferase activity in cells transfected with wild-type constructs, but luciferase activity was not affected in cells transfected with mutant constructs (Fig. 8f, g), indicating that miR-218-5p directly targets hsa_circ_0058124 and NUMB. Then, we determined the expression of miR-218-5p and NUMB by qRT-PCR in 20 pairs of PTC and matched normal tissues. We observed that miR-218-5p expression was downregulated in PTC tissues compared with normal tissues, while the expression of NUMB was significantly upregulated in PTC tissues (Additional file 2: Figure S3). There was also a negative correlation of hsa_circ_0058124 and miR-218-5p, similar to that of miR-218-5p and NUMB (Additional file 2: Figure S4). Moreover, miR-218-5p expression was significantly upregulated with hsa_circ_0058124 knockdown (Fig. 8h), and the expression of NUMB at both the mRNA (Fig. 8i) and protein level (Fig. 8j) were significantly decreased after hsa_circ_0058124 silencing in TPC-1 and K1 cells. As shown in Fig. 8k, we demonstrated that the *NOTCH3 pathway* was regulated by hsa_circ_0058124/miR-218-5p/NUMB axis. NOTCH3 pathway was significantly activated by hsa_circ_0058124 suppression, while si-hsa_circ_0058124 induced NOTCH3 pathway activation was attenuated with miR-218-5p inhibition or NUMB overexpression. These results indicated that hsa_circ_0058124 suppress NOTCH3 pathway via miR-218-5p/NUMB. Furthermore, miR-218-5p inhibition or NUMB overexpression significantly attenuated the si-hsa_circ_0058124-induced inhibition of growth (Fig. 8l), migration and invasion (Fig. 8m). Taken together, these results suggest that hsa_circ_0058124 promotes PTC progression through miR-218-5p/NUMB axis to suppress NOTCH3 pathway.

### Hsa_circ_0058124 promotes PTC progression through the NOTCH3/GATAD2A axis

**Fig. 9:**
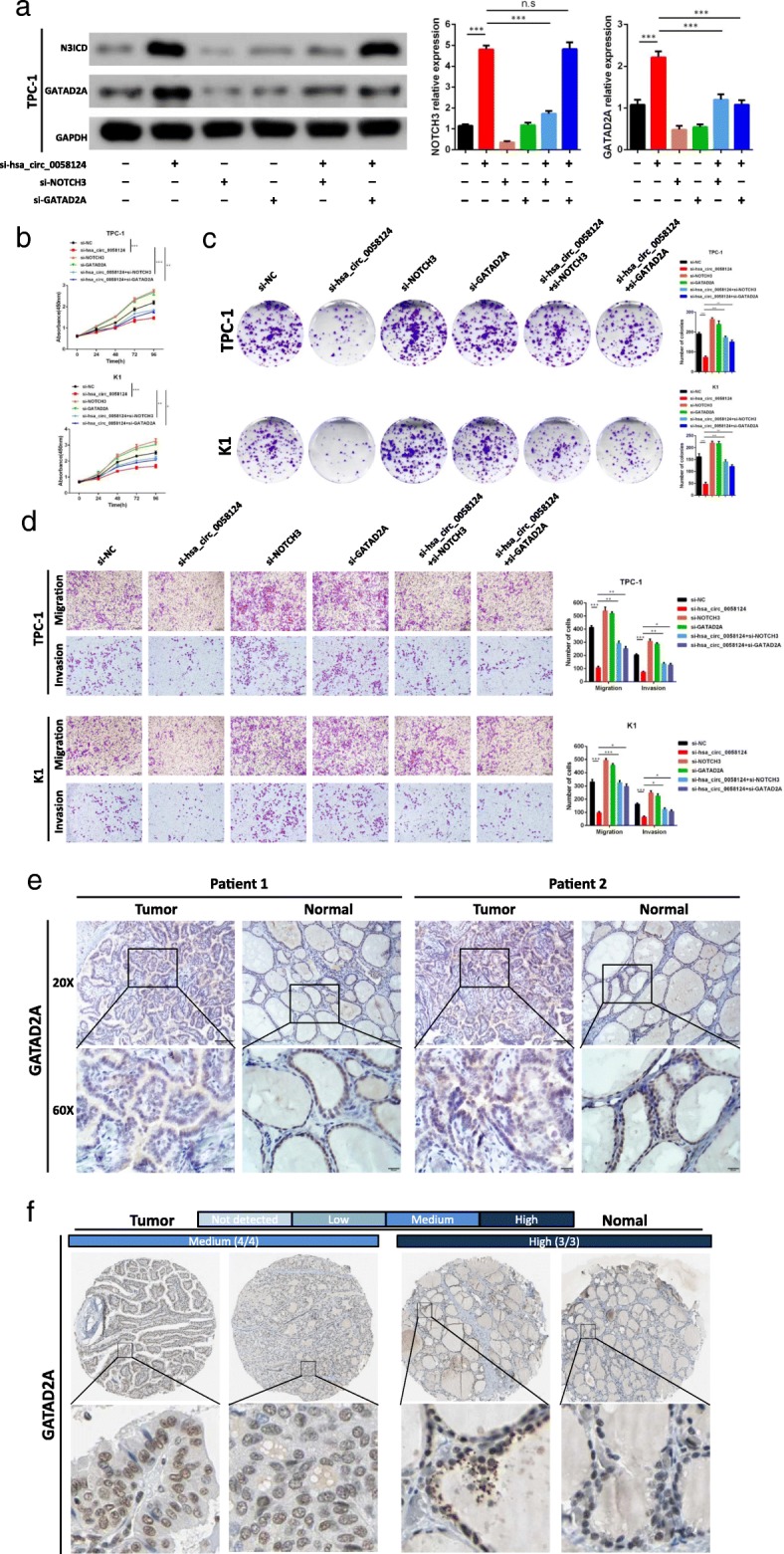
The oncogenic hsa_circ_0058124/NOTCH3/GATAD2A axis in PTC cells. **a** Expression of Notch3 and GATAD2A in TPC-1 cells at protein level analyzed by western blot, after transfection with the indicated siRNAs. **b**, **c** The CCK-8 assays (**b**) and colony formation assays (**c**) were used to evaluate the cell growth after transfection with si-hsa_circ_0058124 or co-transfected with si-hsa_circ_0058124 and si-GATAD2A or si-NC in PTC cells. **d** Transwell assays were applied to evaluate the migration and invasion of PTC cells after transfection with si-hsa_circ_0058124 or co-transfected with si-hsa_circ_0058124 and si-GATAD2A or si-NC. **e** Immunohistochemistry analysis of GATAD2A protein levels in PTC tissues. Representative images were shown. Scale bar, 100 μm. **f** Representative images for the expression of GATAD2A in thyroid tumor tissues and normal thyroid tissues are shown with the fraction of samples with antibody staining/protein expressions evaluated as high, medium, low, or not detected based on the blue-scale color coding. Data are presented as the mean ± S.E.M., analyzed using independent samples student’s t-test. ^*^P < 0.05, ^**^P < 0.01 and ^***^P < 0.001

The GATA family is known to interact with the NOTCH signaling pathway [36–38]. Our mRNA-sequence data also showed GATAD2A was significantly dysregulated in si-hsa_circ_0058124 group. Therefore, we questioned whether hsa_circ_0058124 may promote PTC progression through down-regulating NOTCH3/GATAD2A axis. Consistently, hsa_circ_0058124 silencing significantly increased the expression of Notch3 and GATAD2A at the protein level. In addition, blocking the NOTCH3 pathway with si-NOTCH3 also downregulated GATAD2A, even with suppressed hsa_circ_0058124, while suppression of GATAD2A did not significantly affect the NOTCH3 expression in TPC-1 cells (Fig. 9a). These findings indicated that the suppression of GATAD2A by hsa_circ_0058124 depends on NOTCH3.

We confirmed whether the oncogenic effects of hsa_circ_0058124 knockdown were mediated by NOTCH3/GATAD2A axis in PTC cells by cotransfecting si-hsa_circ_0058124 and si-NOTCH3 or si-GATAD2A into two PTC cell lines. Subsequent functional experiments showed that the suppression of NOTCH3 or GATAD2A could partly impair the si-hsa_circ_0058124-induced inhibition of growth (Fig. 9b-c), migration, and invasion (Fig. 9d). Thus, suppression of NOTCH3 or GATAD2A could restore the malignant behavior of hsa_circ_0058124 knockdown. However, the results also indicated that suppression of NOTCH3 or GATAD2A only partly impaired the si-hsa_circ_0058124-induced inhibition of the malignant behavior in PTC cells, which suggested that NOTCH3/GATAD2A axis might just be one of the major downstream target genes regulated by hsa_circ_0058124.

To investigate the GATAD2A expression levels in PTC tissues, we collected 5 PTC tissues with paired adjacent normal tissues, and measured the expression levels of GATAD2A by IHC assays. The results showed that GATAD2A expression was much lower in the PTC tissues compared with adjacent normal tissues (Fig. 9e). We also validated the differential expression of GATAD2A in PTC by examining IHC staining data of thyroid tumor tissues and normal thyroid tissues from the Human Protein Atlas. The fraction of samples with high, medium, low, or not detected levels of protein expression were provided by the blue-scale color-coding. As shown in Fig. 9f, all 4 thyroid tumor samples showed medium expression of GATAD2A, while all 3 normal samples showed high expression. Collectively, these data indicated that the underlying mechanism by which hsa_circ_0058124 promotes PTC progression is associated with a suppression of the NOTCH3/GATAD2A axis.

### The effects of hsa_circ_0058124/NOTCH3/GATAD2A axis in PTC tumor formation in vivo

**Fig. 10:**
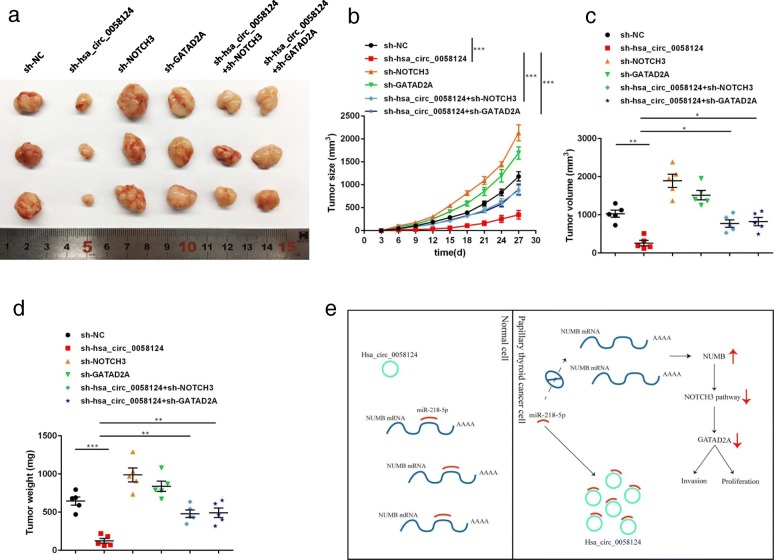
Hsa_circ_0058124/NOTCH3/GATAD2A axis promotes PTC tumor formation in vivo. **a** TPC-1 cells with indicated transfection (3 × 10^6^ cells per mouse, *n* = 5 for each group) were inoculated into nude mice to establish subcutaneous xenograft tumors. The mice were sacrificed at 27 days. The dissected tumors were photographed. Representative macroscopic views showed the tumors in the indicated groups. **b** Tumor sizes were measured every 3 days after the tumors were palpable. **c**, **d** Dissected tumor volumes and weights were measured at 27 days. **e** Schematic model of depicting hsa_circ_0058124 as an oncogene in PTC. ^*^P < 0.05, ^**^P < 0.01 and ^***^P < 0.001

To further explore the effects of hsa_circ_0058124/NOTCH3/GATAD2A axis on PTC cells in vivo, we injected nude mice (aged 5–6 weeks, 5 in each group) with TPC-1 cells transfected with indicated shRNAs (3 × 10^6^ cells) subcutaneously for the control group, the hsa_circ_0058124 stably knocked down group, the NOTCH3 stably knocked down group, the GATAD2A stably silencing group, the stable knock down hsa_circ_0058124 with NOTCH3 inhibition group or the stable knock down hsa_circ_0058124 with suppressing GATAD2A group. Increases in tumor sizes were examined and recorded in each group every 3 days. At 27 days after TPC-1 cell injection, the mice were humanely euthanized, and the tumor volume and weight of the dissected tumors were measured. As demonstrated in Fig. 10a-d, the in vivo tumor formation assay suggested that hsa_circ_0058124 knockdown dramatically inhibited tumor growth when compared to the negative control group, whereas suppression of NOTCH3 or GATAD2A partly abolished this reduction of tumor growth by hsa_circ_0058124 knockdown. Taken together, these findings demonstrated that hsa_circ_0058124 is an oncogenic driver that functions by suppressing the NOTCH3/GATAD2A axis in PTC in vivo.

## Discussion

The incidence of PTC has been rapidly increasing over the past few decades. Although the prognosis of patients with PTC is generally good, tumor invasiveness and metastasis are the major risk factors that lead to poor prognosis [5, 6, 39]. Cancer cell migration and invasion are heterogeneous and adaptive processes that include changes in cell morphology, loss of cell polarity, and gains in cell motility that ultimately result in translocation. Cancer invasion is what lies behind the metastatic that remains the major cause of mortality in patients with cancer [9, 30]. Therefore, the major challenges in PTC research are elucidating the mechanisms underlying the invasiveness and metastasis of PTC and developing novel clinical strategies that could intervene the progression of PTC.

In the present study, we investigated the circRNAs that may contribute to the tumorigenesis and invasiveness in PTC by selecting eight PTC samples (including four invasive extrathyroidal extension tumor samples with metastasis and four non-invasive tumor samples with no metastasis) and four matching adjacent normal tissue samples. We then divided these tissue samples into two sets (invasive tumor vs adjacent normal tissue set and invasive vs non-invasive tumor set) and used ceRNA microarray analysis to identify dysregulated circRNAs. These upregulated and downregulated circRNAs and mRNAs were located on different chromosomes and formed a coexpression network for PTC. Combined with the results of previous studies showing that circRNAs are strongly related to thyroid cancer [20, 21], we found that specific circRNAs are candidate biomarkers and molecular targets for PTC.

Our investigations identified a novel circRNA hsa_circ_0058124 in PTC pathogenesis. We demonstrated that hsa_circ_0058124 was present in PTC tissues and cell lines and was resistant to RNase R. We found that hsa_circ_0058124 was frequently upregulated in PTC tissues and its upregulation was also associated with poor prognosis. The results also revealed hsa_circ_0058124 significantly promoted the growth, proliferation, invasiveness, and migration of PTC in vivo and in vitro. These findings are of great significance because this is the first study to demonstrate the differentially expression circRNAs of invasive PTC compared with ordinary PTC. The results also pointed to critical roles of a previously uncharacterized circRNA, hsa_circ_0058124, involved in the migratory and invasive capacities of PTC, and suggested that hsa_circ_0058124 might function as a tumor oncogene and an unfavorable prognostic biomarker. Our findings also indicated an involvement of the NOTCH pathway.

The NOTCH pathway is highly conserved in evolution and plays important roles in the stem cell maintenance, cellular differentiation, and determination [40]. Canonical NOTCH signaling consists of four NOTCH receptors (NOTCH1–4) and their ligands (Delta-like 1, 3, and 4 and Jagged 1 and 2). In mammals, all NOTCH receptors have a similar structure consisting of extracellular, transmembrane, and intracellular subunits [41]. In the most widely accepted core pathway of NOTCH activation, ligand binding unfolds the negative regulatory region to permit the first cleavage through metalloproteinases of the ADAM family. Subsequently, the γ-secretase complex, as the second cleavage mediator, performs an intramembrane cleavage releasing the NOTCH intracellular domain (NICD), which then translocates to the nucleus [42]. Following NOTCH activation, downstream targets, such as the hairy and enhancer of split (HES) family and the hairy-related transcription factor, are expressed and in turn orchestrate the NOTCH-induced nuclear reprogramming [43]. NOTCH signaling pathway participated in the development of a variety of cancers by regulating the tumor microenvironment, tumorigenesis, progression, angiogenesis, invasion and metastasis [44, 45]. NOTCH has also been implicated in cancer cell metabolism, survival and drug resistance; maintenance of cancer stem cells; epithelial-mesenchymal transition; and genomic instability [46]. Our unbiased transcriptome profile in TPC-1 cells transfected with si-hsa_circ_0058124 by RNA sequencing indicated that the NOTCH3 signaling pathway is a functional downstream target for hsa_circ_0058124. As for how hsa_circ_0058124 modulate NOTCH3 pathway, we assumed that hsa_circ_0058124 suppress NOTCH3 pathway by sponging miRNA and then upregulating NUMB expression, on basis of circRNA often acting as miRNA sponge and NUMB as a strong suppressor of the NOTCH pathway. Luciferase assays have been shown efficiently identify the precise and authentic interactions between miR-218-5p and hsa_circ_0058124 or NUMB. Our results demonstrated that hsa_circ_0058124 suppresses the NOTCH3 pathway via miR-218-5p/NUMB. The results further suggested an involvement of GATAD2A.

GATAD2A (GATA zinc finger domain containing 2A) is a subunit of the nucleosome remodeling and histone deacetylation (NuRD) complex, which is a key factor involved in various biological progresses including tumor growth inhibition, cellular differentiation, embryonic development and the general repression of transcription [47, 48]. A previous study reported the differential expression of GATAD2A in thyroid cancer and suggested its possible involvement in thyroid cancer progression [49]. Meanwhile, several reports have shown interactions between the GATA family and the NOTCH signaling pathway [36–38]. Therefore, we hypothesized that hsa_circ_0058124 might promote PTC progression via a NOTCH3/GATAD2A axis. Consistently, our findings demonstrated that hsa_circ_0058124 promote PTC progression by suppressing the NOTCH3/GATAD2A axis in vitro and in vivo.

In the current study, we demonstrated that hsa_circ_0058124 promotes PTC tumorigenesis and invasiveness through sponging miR-218-5p and upregulating its target gene NUMB expression, subsequently with suppression of NOTCH3/GATAD2A axis (Fig. 10e). These findings indicate a critical role for the hsa_circ_0058124/NOTCH3/GATAD2A oncogenic axis in the tumorigenesis and invasiveness of PTC and provide a preclinical rationale for the design of novel therapeutic strategies that will target the this axis to improve the clinical outcome of patients with PTC.

## Conclusions

In conclusion, our findings showed the overexpression of a novel circRNA, hsa_circ_0058124, located on 2q35 in PTC cells. Hsa_circ_0058124 acts as an oncogene and significantly promotes PTC cell proliferation, tumorigenicity, and tumor invasiveness both in vitro and in vivo. Hsa_circ_0058124 exerts its oncogenic activity by suppressing the NOTCH3/GATAD2A axis in a miR-218-5p/NUMB dependent manner in PTC cells. This hsa_circ_0058124 is a promising prognostic indicator, and its discovery provides insight into PTC carcinogenesis and aggressiveness, and may facilitate the development of precise approaches for cancer screening and treatment.

## Additional files

Additional file 1: **Table S1.** The primer sequences of the RNAs used in this study. **Table S2.** Sequence of siRNAs used in this study. **Table S3.** Clinical information of twelve samples from eight PTC patients included for ceRNA microarray analysis. **Table S4.** Differentially expressed circRNAs overlapped between the invasive vs. non-invasive tumor set and the invasive tumor vs. adjacent normal tissue set. (DOCX 21 kb)

Additional file 2: **Figure S1.** The circRNA-mRNA coexpression validated by qRT-PCR. **Figure S2.** Hsa_circ_0058124 expression in PTC tissues. **Figure S3.** The miR-218-5p and NUMB expression in PTC and matched normal tissues tissues. **Figure S4.** Correlation between miR-218-5p and hsa_circ_0058124 or NUMB. (PPTX 1455 kb)

Additional file 3: The genomic location and splicing pattern of hsa_circ_0058124 (864 bp). (DOCX 72 kb)

## Data Availability

All data and materials can be provided upon request.

## Abbreviations

bp: Base pairs
ceRNA: Competing endogenous RNA
CircRNAs: Circular RNAs
EdU: 5-ethynyl-2′-deoxyuridine
ETE: Extrathyroidal extension
FISH: Fluorescent in situ hybridization
GAPDH: Glyceraldehyde-3-phosphate dehydrogenase
GSEA: Gene Set Enrichment Analysis
KEGG: Kyoto Encyclopedia of Genes and Genomes
OD: Optical density
PTC: Papillary thyroid cancer
qRT-PCR: Quantitative real-time polymerase chain reaction
RNA-seq: RNA sequencing
shRNAs: Short hairpin RNA
siRNAs: Small interfering RNAs
TC: Thyroid cancer
