# Correction to: Hsa_circ_0058124 promotes papillary thyroid cancer tumorigenesis and invasiveness through the NOTCH3/GATAD2A axis

**DOI:** 10.1186/s13046-020-01560-8

**Published:** 2020-04-01

**Authors:** Yao Yao, Xinyuan Chen, Hong Yang, Wei Chen, Yichun Qian, Zhongyi Yan, Tian Liao, Weiping Yao, Wenlan Wu, Tonghua Yu, Yun Chen, Yuan Zhang

**Affiliations:** 1 Department of Head and Neck Surgery, Jiangsu Cancer Hospital & Jiangsu Institute of Cancer Research & The Affiliated Cancer Hospital of Nanjing Medical University, Nanjing, 210009 China; 2 Department of Immunology, Key Laboratory of Immune Microenvironment and Disease, Nanjing Medical University, Nanjing, 211166 China; 3 Jiangsu Key Lab of Cancer Biomarkers, Prevention and Treatment, Collaborative Innovation Center for Cancer Personalized Medicine, Nanjing Medical University, Nanjing, 211166 China; 4 Jiangsu Key Laboratory of Oral Disease, Nanjing Medical University, Jiangsu, 210029 China; 5 Department of Oral and Maxillofacial Surgery, Affiliated Hospital of Stomatology, Nanjing Medical University, Jiangsu, 210029 China; 6 Department of Head and Neck Surgery, Fudan University Shanghai Cancer Center, Shanghai, 200032 China; 7 Department of Oncology, Shangha Medical College, Fudan University, Shanghai, 200032 China

**Correction to: J Exp Clin Cancer Res**

**https://doi.org/10.1186/s13046-019-1321-x**

In the original publication of this manuscript [1], the Fig. 6a invasion si-hsa_circ_0058124_2# group (row 2 right and row 3 right) and Fig. 9c TPC-1 clone formation assay control group (row 1 left) were misplaced and need to be revised. The updated figures are shown below.

The authors apologize for the inconvenience that the corrections caused.

**Fig. 6:**
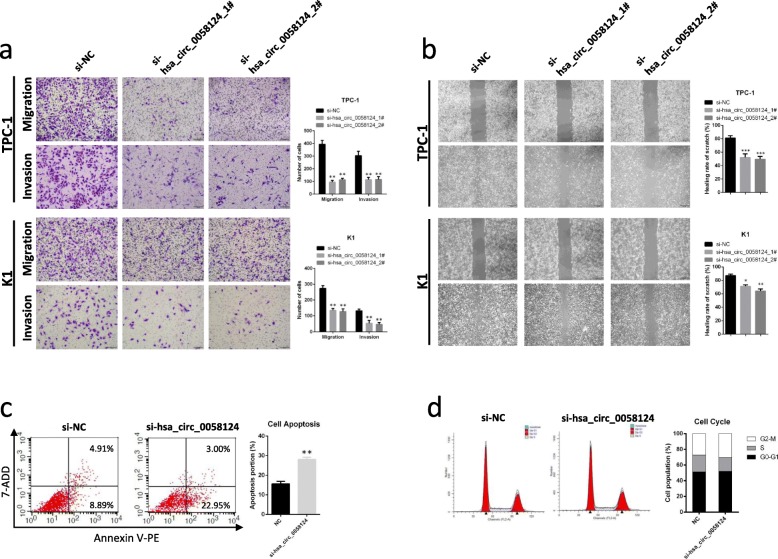
Hsa_circ_0058124 affects the migration, invasion abilities, and apoptosis of PTC cells in vitro. **a** Representative images and quantification results of cell migration and invasion abilities of TPC-1 and K1 cells harboring control or hsa_circ_0058124 siRNAs. **b** Scratch wound assays in hsa_circ_0058124-deficient PTC cells and corresponding controls. **c**, **d** Flow cytometry assays showed the rate of apoptosis (**c**) and the cell cycle distributions (**d**) in TPC-1 cells transfected with si-NC or si-hsa_circ_0058124. ^*^P < 0.05, ^**^P < 0.01 and ^***^P < 0.001

**Fig. 9:**
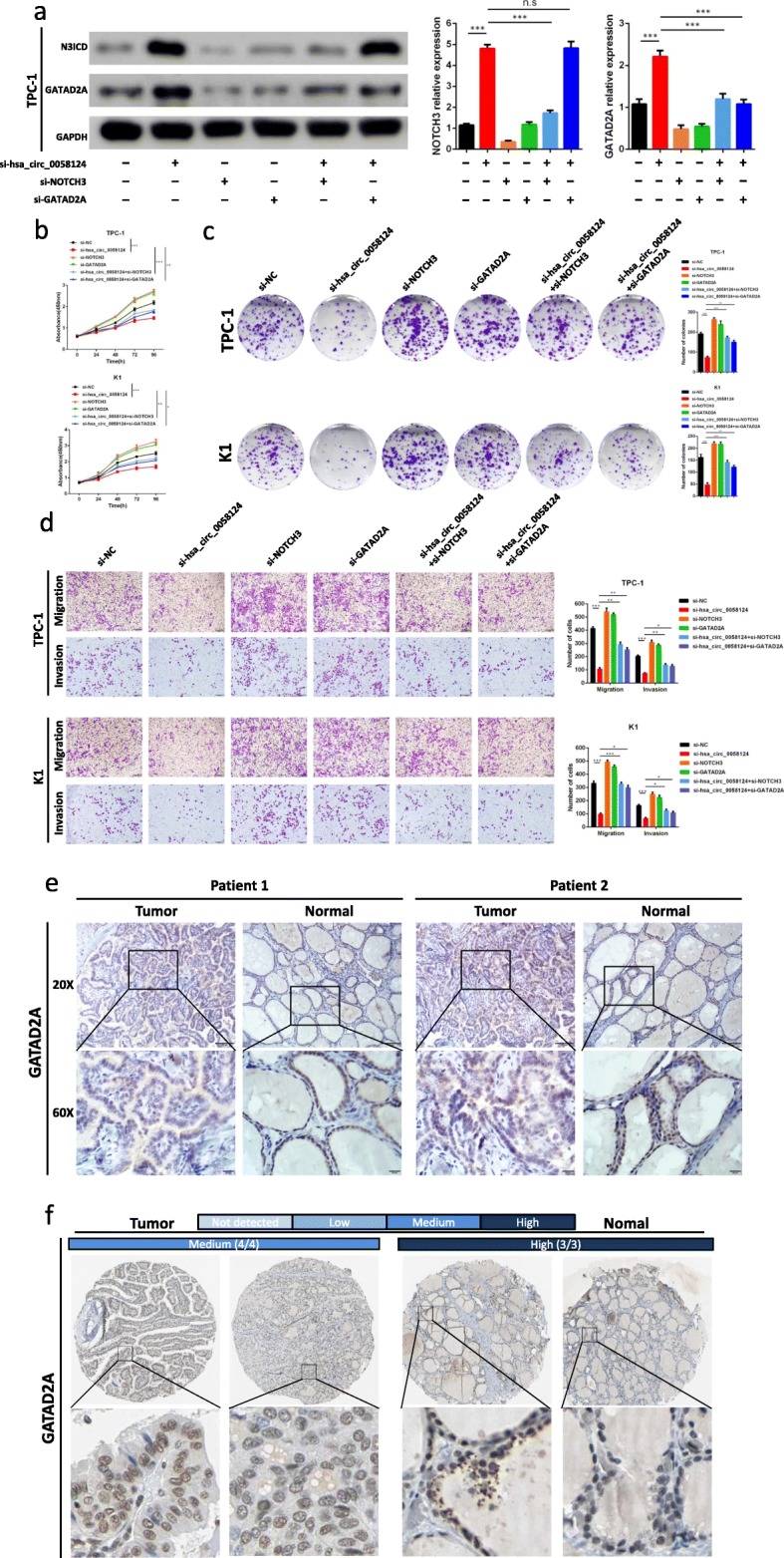
The oncogenic hsa_circ_0058124/NOTCH3/GATAD2A axis in PTC cells. **a** Expression of Notch3 and GATAD2A in TPC-1 cells at protein level analyzed by western blot, after transfection with the indicated siRNAs. **b**, **c** The CCK-8 assays (**b**) and colony formation assays (**c**) were used to evaluate the cell growth after transfection with si-hsa_circ_0058124 or co-transfected with si-hsa_circ_0058124 and si-GATAD2A or si-NC in PTC cells. **d** Transwell assays were applied to evaluate the migration and invasion of PTC cells after transfection with si-hsa_circ_0058124 or co-transfected with si-hsa_circ_0058124 and si-GATAD2A or si-NC. **e** Immunohistochemistry analysis of GATAD2A protein levels in PTC tissues. Representative images were shown. Scale bar, 100 μm. **f** Representative images for the expression of GATAD2A in thyroid tumor tissues and normal thyroid tissues are shown with the fraction of samples with antibody staining/protein expressions evaluated as high, medium, low, or not detected based on the blue-scale color coding. Data are presented as the mean ± S.E.M., analyzed using independent samples student’s t-test. ^*^P < 0.05, ^**^P < 0.01 and ^***^P < 0.001
