# Supplementary material for: Hsa_circ_0058124 promotes papillary thyroid cancer tumorigenesis and invasiveness through the NOTCH3/GATAD2A axis
Source: J Exp Clin Cancer Res. 2019 Jul 19;38:318. doi: 10.1186/s13046-019-1321-x (PMC6642504; doi:10.1186/s13046-019-1321-x)
Supplement: Supplementary file 1 — Table S1. The primer sequences of the RNAs used in this study. Table S2. Sequence of siRNAs used in this study. Table S3. Clinical information of twelve samples from eight PTC patients included for ceRNA microarray analysis. Table S4. Differentially expressed circRNAs overlapped between the invasive vs. non-invasive tumor set and the invasive tumor vs. adjacent normal tissue set. (DOCX 21 kb) [file 13046_2019_1321_MOESM1_ESM.docx]

Additional file 1

Table S1. The primer sequences of the RNAs used in this study

| Gene | Primer (5’→3’) |
| --- | --- |
| hsa_circ_0058124-F | CTTTGCAGTGAGCCATGGGA |
| hsa_circ_0058124-R | GAGTCGTCTCTCCTGTCACG |
| hsa_circ_0058127-F | GGCACCCCACGCTCA |
| hsa_circ_0058127-R | TTCTGTACCCTGTGATGGGAG |
| hsa_circ_0058129-F | TCAACAAGAAACCACTGGCAC |
| hsa_circ_0058129-R | CACACACTCTAACATGTAACCACCA |
| hsa_circ_0058092-F | GAATAATCAGAAGAGCGAGCCC |
| hsa_circ_0058092-R | GCACCAGGAAGTTGGTTAAATCA |
| hsa_circ_0058107-F | AGAGTCAGCCTCTGGTTCAGACT |
| hsa_circ_0058107-R | ATGTTGGTGAATCGCAGGTCAG |
| hsa_circ_0058128-F | CAACAAGAAACCACTGGCACC |
| hsa_circ_0058128-R | CTGTCTGTGACACAGTGGCCATA |
| hsa_circ_0058112-F | AACCTCACTCCAGGCACAGAG |
| hsa_circ_0058112-R | GAAGGGATGCAGAGGACCAGA |
| hsa_circ_0058130-F | AACATCCCTGACCTGCTTCCT |
| hsa_circ_0058130-R | CAGAGGACCAGAGAAGTTGTGG |
| hsa_circ_0058149-F | GTACATGCTTCGGTCAGGGTC |
| hsa_circ_0058149-R | CCCACAGAGTAGACCACACCAC |
| hsa_circ_0060983-F | ACCACGTAGGTACTGTGTGAG |
| hsa_circ_0060983-R | GTGACAACATCGGTGAAGGG |
| hsa_circ_0011290-F | CTGGACTTCGTGCCTTACTGAG |
| hsa_circ_0011290-R | TGGCACAGATCTTCACCCACA |
| hsa_circ_0030979-F | CTCAAGTGGATCGACAGGTCC |
| hsa_circ_0030979-R | CTTGGTAGAGACAGTGGGCTG |
| hsa_circ_0085413-F | CCTCTGAAGGGCTTGACACC |
| hsa_circ_0085413-R | CTGCAGATTCAGCTGGTTAGTTT |
| Liner FN1-F | TACCATCAGAGAACAAACACTAATG |
| Liner FN1-R | AAGAACTCTAAGCTGGGTCTGC |
| GAPDH-F | CTCTGCTCCTCCTGTTCGAC |
| GAPDH-R | GCGCCCAATACGACCAAATC |
| GAPDH divergent-F | GTATTGGGCGCCTGGTCACC |
| GAPDH divergent-R | CGGCTGGCGACGCAAAAGAA |
| U6-F | GTGCTCGCTTCGGCAGCACATATAC |
| U6-R | AAAAATATGGAACGCTTCACGAATTTG |
| JAG1-F | GTCCATGCAGAACGTGAACG |
| JAG1-R | GCGGGACTGATACTCCTTGA |
| JAG2-F | TGGGCGGCAACTCCTTCTA |
| JAG2-R | GCCTCCACGATGAGGGTAAA |
| MFNG-F | TGGGCTATATCATTGAGTGCAAG |
| MFNG-R | AAAGACACCGTAGCTGAGGGT |
| LFNG-F | GTCAGCGAGAACAAGGTGC |
| LFNG-R | GATCCGCTCAGCCGTATTCAT |
| NOTCH1-F | GAGGCGTGGCAGACTATGC |
| NOTCH1-R | CTTGTACTCCGTCAGCGTGA |
| NOTCH2-F | GATCACCCGAATGGCTATGAAT |
| NOTCH2-R | GGGGTCACAGTTGTCAATGTT |
| NOTCH3-F | ATGCAGGATAGCAAGGAGGA |
| NOTCH3-R | AAGTGGTCCAACAGCAGCTT |
| NOTCH4-F | CCTGGCTCCTTCAACTGCC |
| NOTCH4-R | GCAAGTAGGTCCAGACAGGT |
| MAML1-F | AGACCTCAACCTTAACGAGCA |
| MAML1-R | TGTGGAGAGCCTAACTGTTCTT |
| MAML2-F | AAGCGACCCAATGGCTTTGT |
| MAML2-R | GAGTGTTAGTCTTTCGCAGGG |
| MAML3-F | GAGGATAGCTTCACCATCTTGC |
| MAML3-R | CCTCAGGAACCGTGTTGGC |
| HES1-F | TCAACACGACACCGGATAAAC |
| HES1-R | GCCGCGAGCTATCTTTCTTCA |
| HES2-F | AACCAGAGCCTGAGCCAGCTTA |
| HES2-R | TGCAGGAAGCGCACGGTCATTT |
| HES6-F | GCTGGAGAACGCCGAAGTGCT |
| HES6-R | TGGACACGAACGTGTGCACCTC |
| HEY1-F | GTTCGGCTCTAGGTTCCATGT |
| HEY1-R | CGTCGGCGCTTCTCAATTATTC |
| HEY2-F | AAGGCGTCGGGATCGGATAA |
| HEY2-R | AGAGCGTGTGCGTCAAAGTAG |
| NUMB-F | AGGCCAGTCGTCCACATCA |
| NUMB-R | GGTACTTAACCGGGAAGCTACAT |
| GATAD2A-F | CTCTTTGGGTGTGGAGGCTGT |
| GATAD2A-R | CATCTGGTGGCAAACATTTCTT |

Table S2. Sequence of siRNAs used in this study

| siRNAs | Sequence (5’→3’) |
| --- | --- |
| si-hsa_circ_0058124_1# | CAGACAACCAGCAACACCGTT |
| si-hsa_circ_0058124_2# | CAACAGACAACCAGCAACATT |
| si-NOTCH3_1# | GCUGCUGUUGGACCACUUUTT |
| si-NOTCH3_2# | GCAGAUGACACAUCAGCUATT |
| si-GATAD2A_1# | CCACAGCAUUAGGCAACAUTT |
| si-GATAD2A_2# | GGCAGACAUUCUGAGAGAATT |
| Negative control siRNA | UUCUCCGAACGUGUCACGUTT |

Table S3. Clinical information of twelve samples from eight PTC patients included for ceRNA microarray analysis

| NO. | Age (years) | Gender | TNM classification | Clinical stage | Subtype  (WHO classification) |
| --- | --- | --- | --- | --- | --- |
| 1 | 60 | Female | T4N1M1 | Ⅳ | Classic PTC |
| 2 | 59 | Female | T4N1M1 | Ⅳ | Classic PTC |
| 3 | 72 | Female | T4N1M1 | Ⅳ | Classic PTC |
| 4 | 64 | Male | T4N1M1 | Ⅳ | Classic PTC |
| 5 | 56 | Female | T1N0M0 | Ⅰ | Classic PTC |
| 6 | 57 | Male | T2N0M0 | Ⅰ | Classic PTC |
| 7 | 66 | Female | T1N0M0 | Ⅰ | Classic PTC |
| 8 | 61 | Female | T1N0M0 | Ⅰ | Classic PTC |

Table S4. Differentially expressed circRNAs overlapped between the invasive vs. non-invasive tumor set and the invasive tumor vs. adjacent normal tissue set

| NO. | CircRNA ID | Fold change | *P* value | Chromosome | Regulation | Host gene |
| --- | --- | --- | --- | --- | --- | --- |
| 1 | hsa_circ_0058124 | 2.3763 | 0.0073 | chr2 | up | FN1 |
| 2 | hsa_circ_0058127 | 2.3559 | 0.0096 | chr2 | up | FN1 |
| 3 | hsa_circ_0058129 | 2.3114 | 0.0025 | chr2 | up | FN1 |
| 4 | hsa_circ_0058092 | 2.3068 | 0.0097 | chr2 | up | FN1 |
| 5 | hsa_circ_0058107 | 2.2730 | 0.0049 | chr2 | up | FN1 |
| 6 | hsa_circ_0058128 | 2.2477 | 0.0044 | chr2 | up | FN1 |
| 7 | hsa_circ_0058112 | 2.2472 | 0.0083 | chr2 | up | FN1 |
| 8 | hsa_circ_0058130 | 2.1903 | 0.0058 | chr2 | up | FN1 |
| 9 | hsa_circ_0058149 | 2.1517 | 0.0073 | chr2 | up | FN1 |
| 10 | hsa_circ_0060983 | 2.0905 | 0.0085 | chr20 | up | VAPB |
| 11 | hsa_circ_0011290 | 2.0124 | 0.0079 | chr1 | up | SERINC2 |
| 12 | hsa_circ_0030979 | 0.4949 | 0.0070 | chr13 | down | F10 |
| 13 | hsa_circ_0085413 | 0.4380 | 0.0030 | chr8 | down | COL14A1 |
