# Supplementary material for: Hsa_circ_0058124 promotes papillary thyroid cancer tumorigenesis and invasiveness through the NOTCH3/GATAD2A axis
Source: J Exp Clin Cancer Res. 2019 Jul 19;38:318. doi: 10.1186/s13046-019-1321-x (PMC6642504; doi:10.1186/s13046-019-1321-x)
Supplement: Supplementary file 2 — Figure S1. The circRNA-mRNA coexpression validated by qRT-PCR. Figure S2. Hsa_circ_0058124 expression in PTC tissues. Figure S3. The miR-218-5p and NUMB expression in PTC and matched normal tissues tissues. Figure S4. Correlation between miR-218-5p and hsa_circ_0058124 or NUMB. (PPTX 1455 kb) [file 13046_2019_1321_MOESM2_ESM.pptx]

## Slide 1
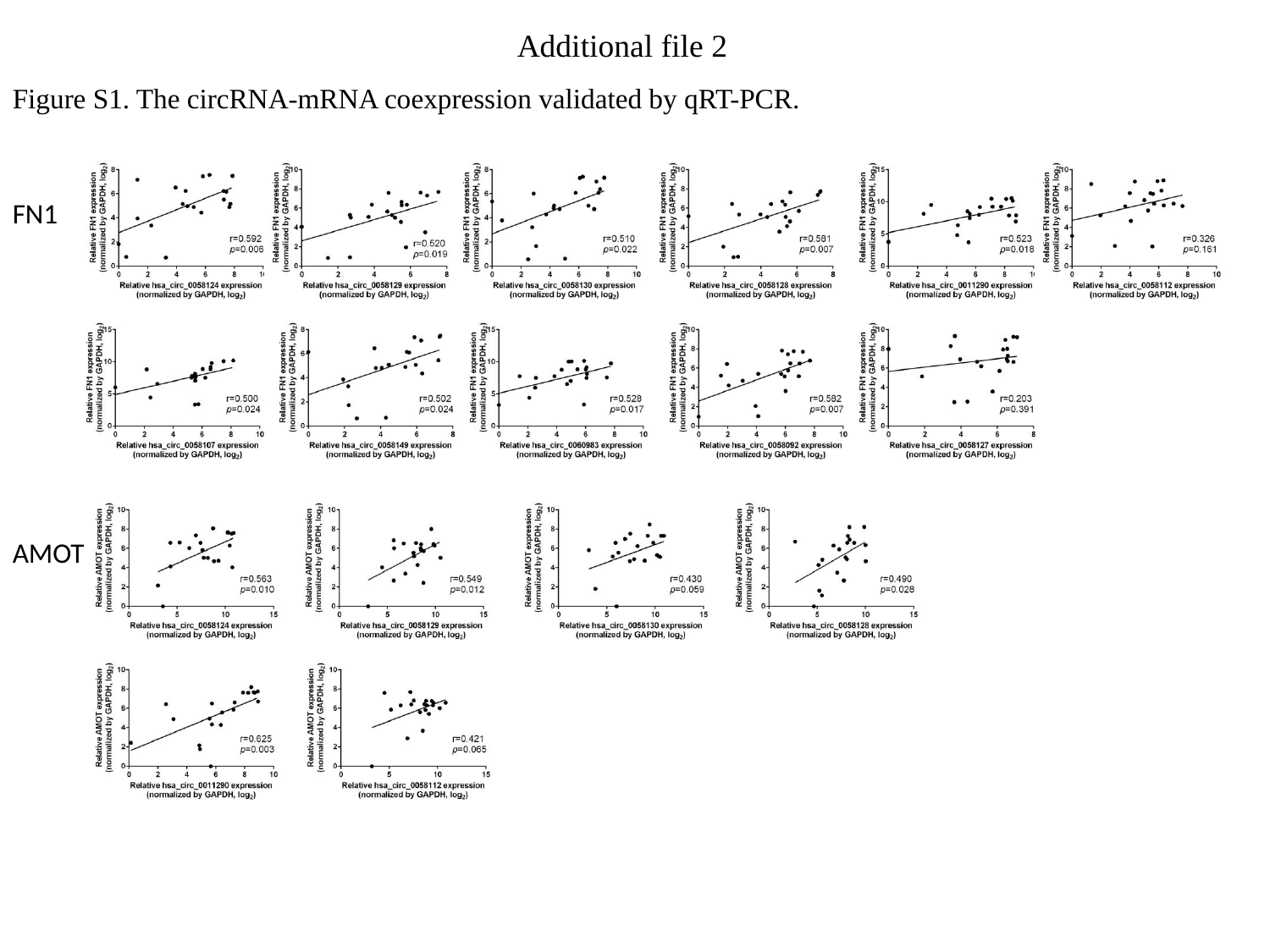

Additional file 2
Figure S1. The circRNA-mRNA coexpression validated by qRT-PCR.
FN1
AMOT

## Slide 2
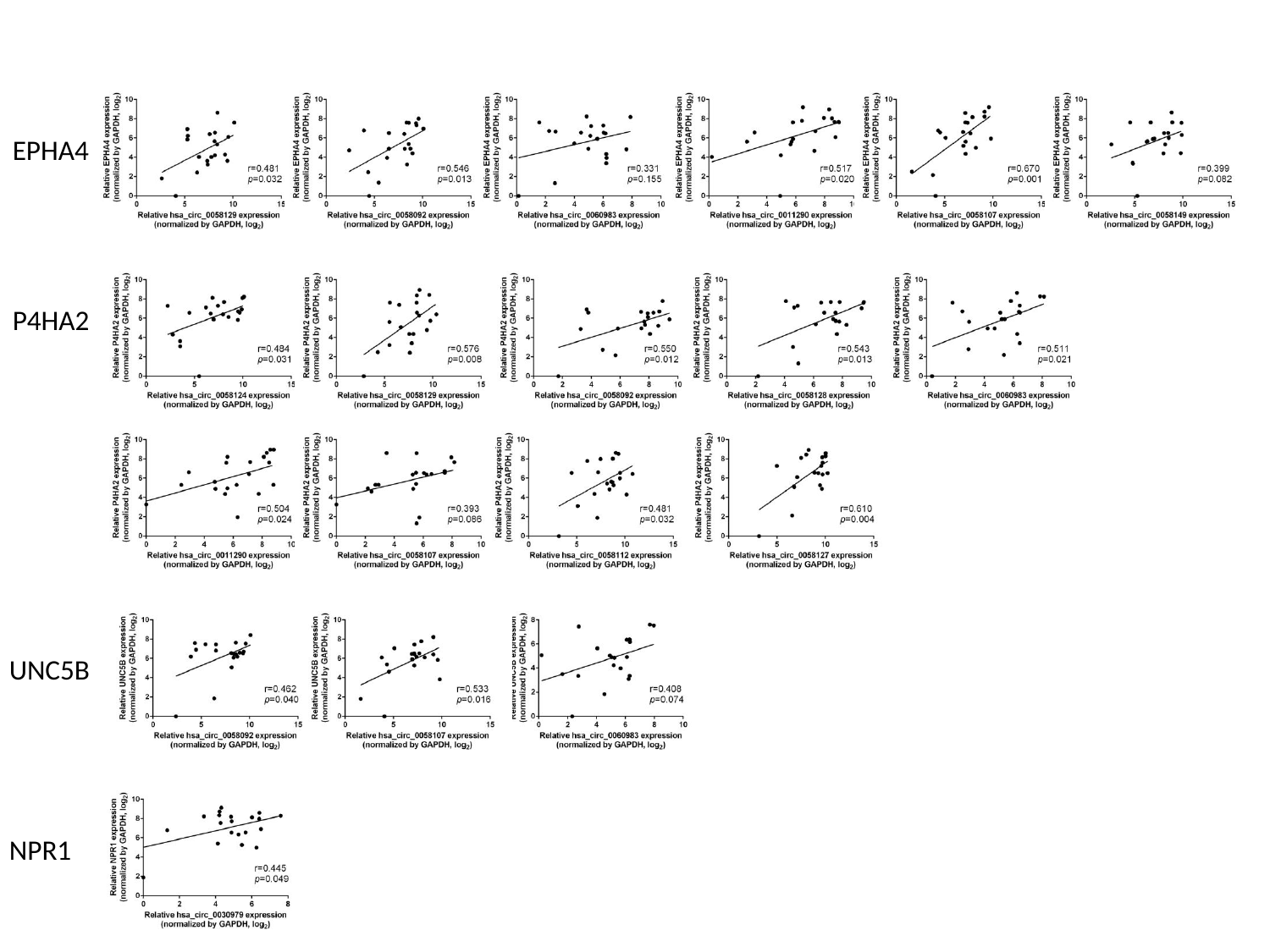

EPHA4
P4HA2
UNC5B
NPR1

## Slide 3
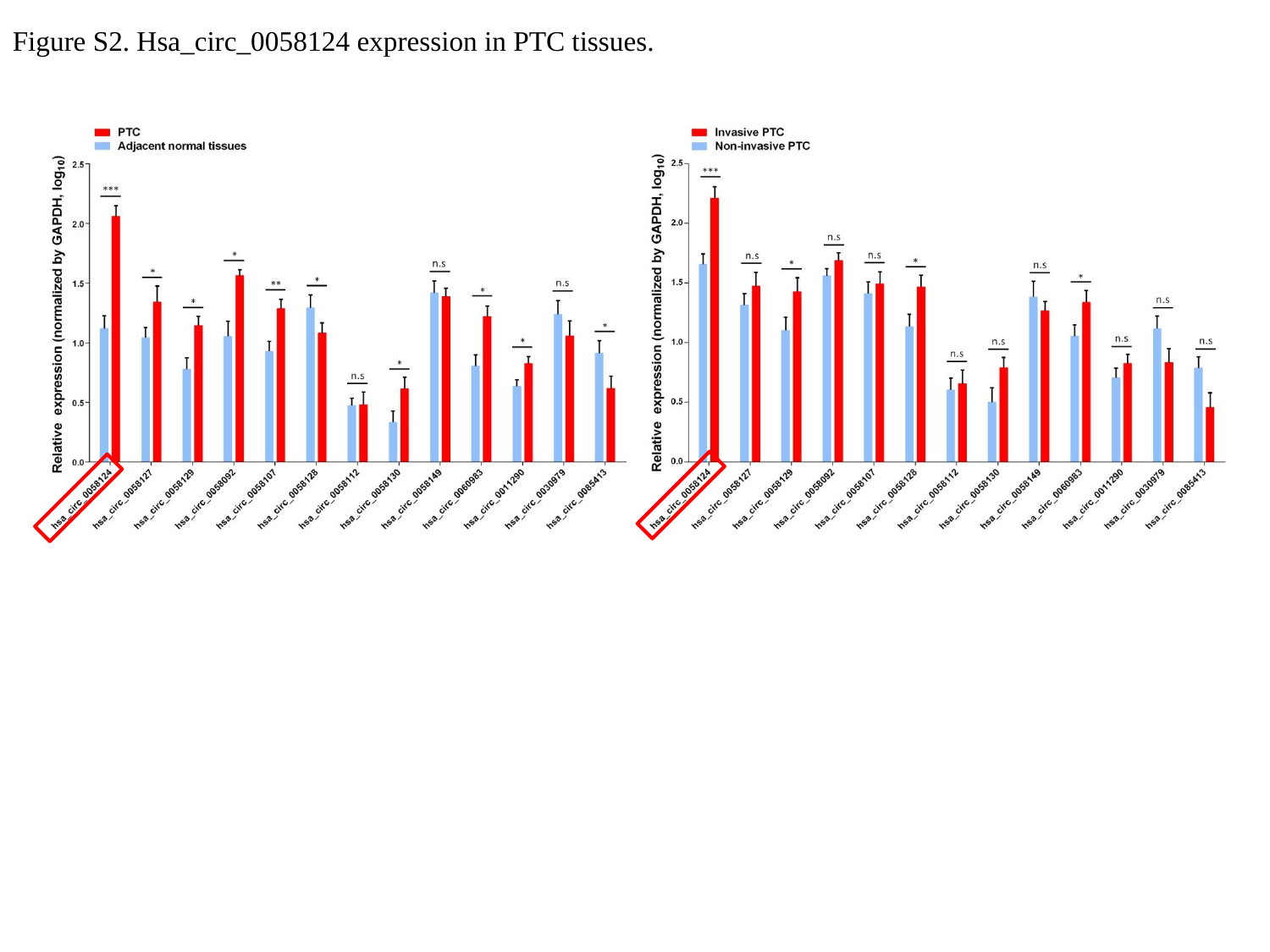

Figure S2. Hsa_circ_0058124 expression in PTC tissues.

## Slide 4
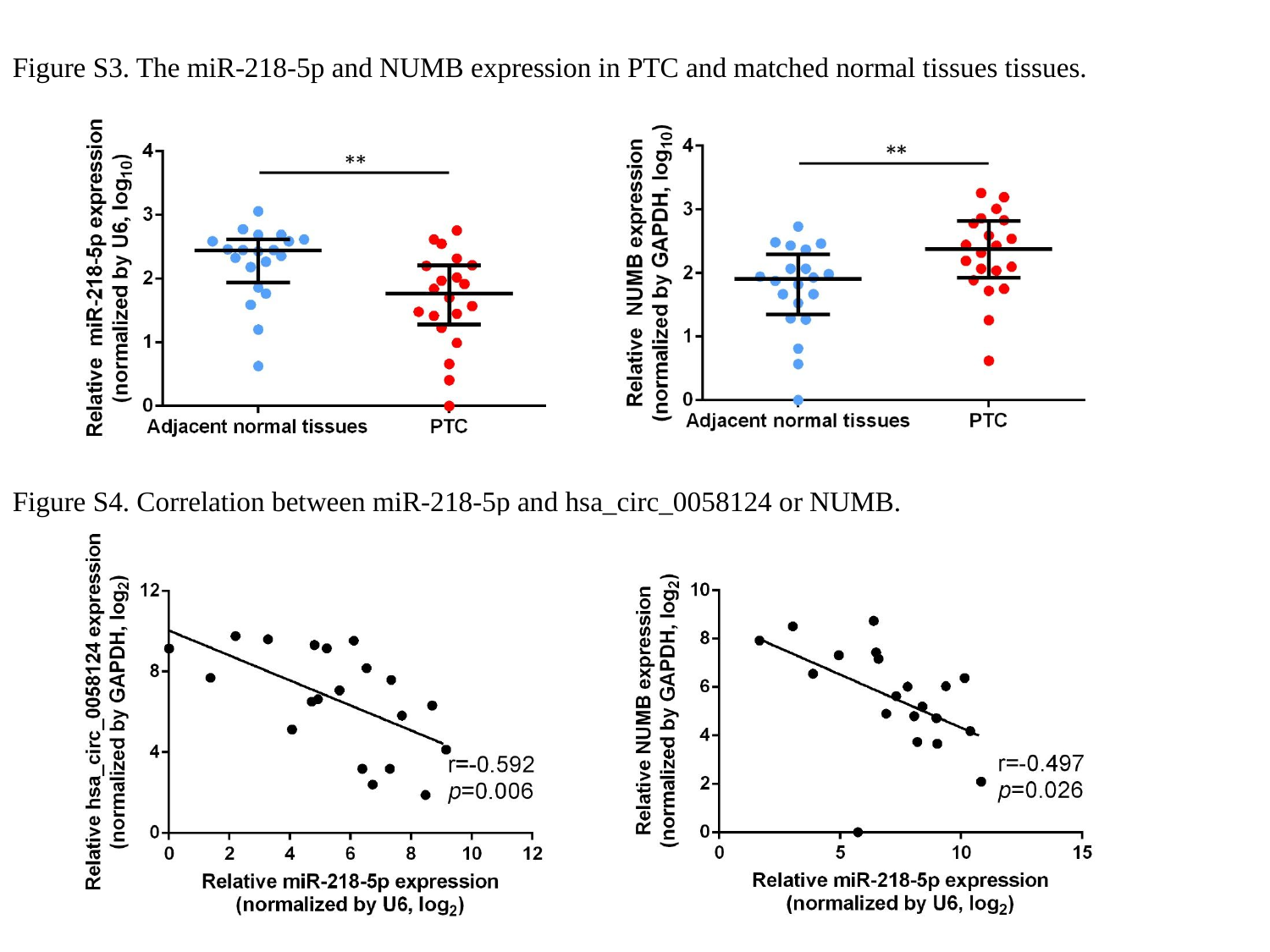

Figure S3. The miR-218-5p and NUMB expression in PTC and matched normal tissues tissues.
Figure S4. Correlation between miR-218-5p and hsa_circ_0058124 or NUMB.
