# Supplementary material for: Hsa_circ_0058124 promotes papillary thyroid cancer tumorigenesis and invasiveness through the NOTCH3/GATAD2A axis
Source: J Exp Clin Cancer Res. 2019 Jul 19;38:318. doi: 10.1186/s13046-019-1321-x (PMC6642504; doi:10.1186/s13046-019-1321-x)
Supplement: Supplementary file 3 — The genomic location and splicing pattern of hsa_circ_0058124 (864 bp). (DOCX 72 kb) [file 13046_2019_1321_MOESM3_ESM.docx]

Additional file 3

The genomic location and splicing pattern of hsa_circ_0058124 (864 bp)

**Homo sapiens_FN1_GENOMIC**

>NC_000002.12 Homo sapiens chromosome 2, GRCh38.p12 Primary Assembly

ORIGIN

1 cggggacagc ccggcgggtc tctcctcccc cgcgccccgg gcctccagag gggcgggagg

61 ggaccgtccc atataagccc cggctcccgg cgctcggacg cccgcgccgg ctgtgctgca

121 cagggggagg agagggaacc ccaggcgcga gcgggaagag gggacctgca gccacaactt

181 ctctggtcct ctgcatccct tctgtccctc cacccgtccc cttccccacc ctctggcccc

241 caccttcttg gaggcgacaa cccccgggag gcattagaag ggatttttcc cgcaggttgc

301 gaagggaagc aaacttggtg gcaacttgcc tcccggtgcg ggcgtctctc ccccaccgtc

361 tcaacatgct taggggtccg gggcccgggc tgctgctgct ggccgtccag tgcctgggga

421 cagcggtgcc ctccacggga gcctcgaaga gcaagaggca ggctcagcaa atggttcagc

481 cccagtcccc ggtggctgtc agtcaaagca agcgtgagta ctgaccgcgg gctgaaacag

541 gctgcctcag ggatgggacc ctaaagccga ccaaagttgg ggctgaagtt ttgtgcgcgc

601 gcgtgtgtgc gagtgtgtgc gcgctttact gagagaaacc agctgtgcac acaaaaggac

661 cgagttttga gcacgctggt tctgagggcc tgggatgata agaccgtgca ttggaggacg

721 aggactctgc gactttcccg tgttctaata aattctgcac gttcagattg tccttctagg

781 aattaaccaa aacttgcctt taaagagaaa aatgatgcat gtctataaat tttccgtctg

841 ggattagtgt ggtccttact gctacttatt tccttctgtt aaataattgg tcaaatattt

901 tcaacatggg ggtggaaagg gggtattgaa atagctgtct tgtttctaac taacttggaa

961 gagatgtaat tggttcagac ctctttaggg ccgctcagga tacttcacca agaacagagg

1021 ttggaattct ttccgttttt caaagacaca ccctcctttt gctttgagaa agctgcttaa

1081 agttgtcctt tttgactatt actccaaaag aatatttaag ttccttgcat gttttaaaaa

1141 tgtgacttca attgtctgcc ttccaaaatg tttccaactt ttttatgtag acccctggcc

1201 agatggaaat gacatcattg tatataactt ttagcaaagt taaaaggaaa aaaatatgta

1261 cgtcaatatt cacatgaaga aaattccata attttgggaa aaggagaaat gcaaatgtaa

1321 cgttttcctt caattatttg cagccggttg ttatgacaat ggaaaacact atcagataaa

1381 tcaacagtgg gagcggacct acctaggcaa tgcgttggtt tgtacttgtt atggaggaag

1441 ccgaggtttt aactgcgaga gtaaacctga aggtaagtga caacaagccc catagttagt

1501 atcttttaat acatgaagtg gtaattgtta aactttgcat tagtaagtaa aaatacatac

1561 accatttttc taatagaatt acctgtcatt tcctcttaag tttaaaaact gcttatattt

1621 gcttttcaca tgcttttacc tttaaaacaa agaaacgaat ctttcccaaa ttagttccta

1681 gagtcttctt tttgctttac tctcccaaag tttttgatga gaaaaatgaa agattttgtg

1741 tgtcctccga caaaaaaaat tgcttataaa attttaattt attagaaagc agtctcaaat

1801 cttaaactgt tagtttatga gccagaaaac actttgggga cttacatcgt aaaatgattt

1861 gtcagcggca gttaacacaa aaccattagc cacttcaaag ttctcattcc tttaggacca

1921 atgatatttt tctcataaat tatagcaact ctgtcagaga agcactgatc aggggaaaat

1981 ggaaatcata ggattaacaa ctgtcaaggc cttgtgggag gtggggatct tcgaattgtt

2041 tgtttgtttt tgttttgttt tgtttttgag acagagtctt gctctgtcac caggctggag

2101 tgcagtagca ctatctcagc tcactgcaac ctctgcctcc agggttcaag caattctcct

2161 gcctcagcct cctgagtagc tgggactaca ggcacatgcc accacaccca gctaattttc

2221 atatttttag tagagacggg gtttcaccgt attggtcagg gtggtctcga attcctgacc

2281 tcaggtgatc cacccgcctc agcctcccaa agtgctggga ttacaggcgt gagccactgt

2341 gcccggccaa tcgtttgctt tttatgtgaa ccttgctttg actttctgag tcagagattg

2401 gaatgtgaaa cccttcacaa atctagctct gtcataagtt agtactttat atggcctttt

2461 cctaagagcc tgagattttt ctacaatatg aataatttac agaaaatttg acaatatgtc

2521 aaggtcaaaa accatggcct tattagagct taggataaaa atctgtatct ctcacttcat

2581 tttattcttt gaggtgtacc atgttacttg tggaatagag aagtgggttt tcctttagag

2641 gggattagtg aactagaaaa gcttgtacct aagtgaggct cacatggact ttccttttcc

2701 cctcagctga agagacttgc tttgacaagt acactgggaa cacttaccga gtgggtgaca

2761 cttatgagcg tcctaaagac tccatgatct gggactgtac ctgcatcggg gctgggcgag

2821 ggagaataag ctgtaccatc gcaagtaagg aagagattgt gtaaaatgat gccaaaatat

2881 caaatatgaa tttctctgtt accatcactg tcattttctg ttatccatga ctggatattc

2941 cgaactttga ggtttgcccc tggtgaccag gtactcttaa gtggtcaccc aactggttcc

3001 tgtgtttctt aaagacgggt atgagcacag atggaatcag tgtttgatgt gtgtgcgttt

3061 atgagtgtgt gtgcatttat gagtgtgtgt gtttctgtac atagtagaac caagagacct

3121 cttgggttcc atttcagtaa gacatgctta ggggagttgc ccattttaaa tcacctggat

3181 atcttcaagc tagacaaatc atgagacttt tctgcagtga ctgggaaggt gttcatgaag

3241 agtgaaccag ccatgtgttg tctggtcttc atgtttgcaa tgcagagacc tcttgcacct

3301 cacagaaaca gtctggtttc ttggtgacca gtaggttata cccaggaagc agatgtcact

3361 attcctaggg ataatacaaa attattaacc caatagagtt tgctaaggaa ctttgggaac

3421 cgggctgatt ctcaactcta gtttagctaa ggcactcttt ccagtatgat tcactgggtt

3481 accaatagat tctattaaga tagtatttaa gttttttaat ccattcttta aatataagtc

3541 gtcttaaaga cttctattca aaagaacaag tcccgtgtga ataggcccaa tcaactttcc

3601 ccatatttca tgttagggtt tatccaagtt cacaggcaaa tcgcaagagg caagggtcca

3661 tagtgtttac aatctagttc agcatttgaa tgtgccattg ggcttaacaa cttagaaaac

3721 taccaggatt tccacacttt atatgcatat gtctgtttgc tttccaccaa aatgacattt

3781 ctatcctagg gtaaaataca ggctctccat gctcccaaaa gctggagtgc tgtgcctgat

3841 gtggcctttt cactgaatta gtctcagtct tagcctgctg tgtatgagtg aagaccaagc

3901 ctcccagcct ttcttttctg cttaggaccc aatttcctgt gatctctctg ggaaagcagg

3961 attcatgacc tcttccttgc catccagatt tctctgtggt tttccattgt gttctaagca

4021 agacacttaa ctgaattgac tccaagtgac cagacctgtt aacgtttccc ctgtctctga

4081 tgggaaagct gttgtctgtg tctctacttt agccaaccta agtacctacc atgggtggaa

4141 tatgagacca aaaaaaaaaa tctgttctgc cctctcctaa cattttcgtt gtatcttcaa

4201 cagaccgctg ccatgaaggg ggtcagtcct acaagattgg tgacacctgg aggagaccac

4261 atgagactgg tggttacatg ttagagtgtg tgtgtcttgg taatggaaaa ggagaatgga

4321 cctgcaagcc cataggtgtg tgagtcttag ggctgagcaa gagctgggat gcttagttct

4381 aatgtggggt tggaccagaa tcacatctac ataggtcata gacctgaatt ccagtgaaaa

4441 ccaataaaga aatgggaatt ttgtttgaaa taatgaatta ttatataatc catagtcttc

4501 ttacaggagt tagatcaaaa agtactgact acacatagaa gtcttaactt tgcttcaaaa

4561 gcataaggta gaattgaaag atttagaatg gagtcatttc ttttacctaa tagctgatct

4621 cagattcctc cttcgtcaag atataattta tttaaaagaa aaaaaaatga cactttggac

4681 acatttctat atggaatgtc ctggaccgaa acatgaaata gtgtgtgctt gtcacactct

4741 gctcatttct tttcaaatta aaagttgttg agcttctttg gatctcaatc ctcagttgaa

4801 ttttgtaagt acaagcctga aagtttctgg ctataaattt tactctgttt acttgtcttc

4861 taatttagag gtttttgtct tgttttgtat tgttttgctt tccaatattt aaaaatagct

4921 ttctttgtca ttgtatttag gccactcaaa attcataatt ggtcatttat aattaagatt

4981 ggaattttgc atatgtagtc tcccacagac tagatacata catagatcct tgctactgga

5041 aatgctgctg ggaagtttgg ggctcgctga aaatatgtag tccatgtact tattaggaga

5101 atggaatttc tgcctgccaa ctcagcttga gctttctttt gccttggcta cttactgtgt

5161 gcttagatgc tgggtgtgtc attctttctg aacagagtgc cacttaaaaa aaatgtggct

5221 gaatttttgc ttacacacta cactttaaat tacagggagc ttgcacaatt caaaataacc

5281 tttttttcct gtttttcttc caaatttccc tacagctgag aagtgttttg atcatgctgc

5341 tgggacttcc tatgtggtcg gagaaacgtg ggagaagccc taccaaggct ggatgatggt

5401 agattgtact tgcctgggag aaggcagcgg acgcatcact tgcacttcta gaagtatgtt

5461 ttacatcttt atgttaaaga ttaagccagg tattgttttc tggattccta gagagaaggg

5521 taatactatg ttactcagaa cacatccagt atatcagcat gctttggtaa cttctggaag

5581 tcaagaaaac tttcataacc aacttattcc gcatcttcag agaagactac ataaatagaa

5641 aaacatatca ctttgataag gttcaatctc agctcactgc cactgacata gagttgaaca

5701 aaaggtttag gtttccttct atgtttgaaa tttaaatagg gcacattcac aggctaaatt

5761 gataaaatta aaaagaattt atcccataaa ttaaaatgat ttatctactc tggagttagg

5821 gatagtgtct ctgacctaac gcatttgatt agtgctgtaa agaagctggc ctctggtgtc

5881 tttactgctc cttctaagat tgtcttgggg tcttaattgt tgcctttggg tttgaaggct

5941 ccttttttga tattgtaaac taataacagc tagagagttt gttgaagtaa aacagccatt

6001 aactactggt gttgtaaata agtttaaaat caaatccaaa taatttgaac ctgttttatt

6061 tatctagctg aacccattta actaccttta acatagccat catccaaatt caaattcttt

6121 gctaacaaaa ataggtctct catgaaaagt ggtaaccatt ttgaccaaag ctttcccaga

6181 aacttgctgg tttattagat attttgcatt taaaatgtta ctgtgatcat cagacttcca

6241 agatctttgt ggcaatattt tagcttaaga caaattagat gtctgattca aaccttatct

6301 gttatttaga actctttaaa tagcaagttg ggaaaagttt ctcaaagaga agtcatttat

6361 tccagaaaat tttataagga cttactttgt tcaaggtatt ataggggtgc agatatgaaa

6421 tgaacattag cccagccttc aaagagtact tagggggtca gggagatgag aaagtcttat

6481 acatatttat catctgcaaa acacagtatt aaagatttca acagaaatac tgaaagtagt

6541 gctatggagg ttcaggggat gataatactt ttcgctgggg atttgggaaa aagctctaat

6601 cagtaattat accttcatgc aaacttctat tcttgtggta gatggatgtg ggtgtgtatt

6661 tgtttgaacc tacatcaact attaattttt tttctctaac ccaggagttg caacaatatc

6721 caattcacaa agacatcaga tcctctatac tcacatcgtg gcacagagca aatttggatt

6781 ataatttaaa taatctattt accagataaa tgcacgcata gactaatggt catttagtta

6841 caaattatca ttttatgttg atcccactct tccagtggag ggctaacact gaataatttg

6901 gggctatttt gctagtgatt tttaaatact gtagatgttt gggtataggg gaagggaaaa

6961 taatatttta gtcaaagaaa ttgtgcatcc tctacatttt ttacataaca aatgaagaaa

7021 gagatactac caccttctta tagcttcttt gtagccattg gtgaagaccc tttgatacct

7081 gcttgcctcc ccattgttat aagctttttt ttgtttgctt gttttttttg ttttgttttg

7141 ttttgttttg tttttgagac agtctcaccc tgtcgcccag gctggagtgc aatggtgtga

7201 tctcagctca ttgcaacctc cacctcccgg gttcaagcga ttctcttgcc tcagcttccc

7261 gagtagcttg gattacaggc gcccgccacc acagccggct aatttttttt ggtattttta

7321 gtagagacgg gggtttcacc atgttgccca ggctggtctt gaactcctga ctgcaggtga

7381 tccaccctcc taaagtacta ggattacagg cgtgagccac cgcgcttagc ctgtttttag

7441 ttttctaaag caaggtccct attgaaaggc aggccataaa cagtgatgac taagaaaaat

7501 cctggaagag cctgagaagg aaaaagatga aatataatgc cagagaatga agttagtcaa

7561 aggaacagtg tgaaaacaat aaataaatag ataaatgaaa atgttatttg acagagagat

7621 gaaactagac taaaccattc agctgccttt ccactgtaac aaatgtaatt tcatctttca

7681 gaagtgtaat accttgcagc accagagctg aatatgaaca tattaccaaa aatagattac

7741 caggcataga tagcattcct tttttaagtt tgaattgacc acttgcgact ctcgacctga

7801 tgtatgtatg tgcttccttt gtgacacaga tagatgcaac gatcaggaca caaggacatc

7861 ctatagaatt ggagacacct ggagcaagaa ggataatcga ggaaacctgc tccagtgcat

7921 ctgcacaggc aacggccgag gagagtggaa gtgtgagagg cacacctctg tgcagaccac

7981 atcgagcggt gaggcacagg acgagcaggg gcgggaaatg gggaagcagg tcaagaaata

8041 tttccgcaaa tccatctttc ctttgacatg ccatttgagg ataatttgca gtgtttcagc

8101 taataaccta agataattta cacattattg gttgttaaaa ctttttttaa tgtcaagttt

8161 taaatttttc agaaaaaaag aaaaatgaca tacaaataaa ccttaggggg aaaaaagcca

8221 gatttatctc caaaagataa aactgagttt taaagaatgc tagcatcata aaacttacca

8281 tggatagatc acgcacacac gcacacacac acgtattttg aatatccaaa gttcatttga

8341 aaggaaatga gagttataat taattatatg actacctggt tcttctgcta ggaaaggaca

8401 aaaaaagtgc atttggattt tttgtttgtt tgtttttgaa tgaaatatac ttccctgtcc

8461 cgacattgaa ctctttttgt agtggaaacc atcctttata tgtggtttct atgctctggc

8521 aaactttgtt acattctata aagtaacaca caattatttc cttcatgtat tggcattcga

8581 aattttagaa attcagagag gacttagaga tggccatgaa agacatgata tctaagcatt

8641 ctttttaaaa aacaagtttt aatcattttt ggcatgagaa aaagattttt acgtcataaa

8701 tgtttcataa aaatctgaag agagaaatat ggccaacaag gacgtgcact cctctcatta

8761 tttttaatat gttttgatta actttttact atatgatgtg ccaacatcat tacgtagtgt

8821 ctcagccatc cttcaattaa aaatattaat tgttctaatt tttcttcttt tgatgagttt

8881 ttgtcttgct ttgagcactt atgaaggtga acaagattag atttgataat atctttgagt

8941 tattttatta tcattaataa aattgctact ggccaaaaaa aattataaac atcggccacg

9001 cgcggtggct cacgcctgta atcccagcac tttgggaggc cgaggcaggc ggatcacgag

9061 gtcaggagat caagaccatc ctggctaaca cggtgaaacc ccatctctac taaaaataca

9121 aaaaattaac caggcgttgt ggcgggcgcc tgtagtccca gctactcggg aggctgaggc

9181 aggagaatgg catgaacccg ggaggtggag tttgcagtga cccgagatcg caccactgca

9241 ctccagcctg ggtgatacag cgagacccca tctcaaaaaa ataaaataaa ataaaaaata

9301 aaaaaaatta taaatgtcag tctaccaaaa tagattaaaa gtgtaggtgg gaattaaatg

9361 gggataaaca ctcaataaat gttagctata tatgaatatt gccaatactg aaaagattcc

9421 attgttcaaa aaagtttgag aagcaatggg ttaaacaaaa tggaacctgc tctgcagaat

9481 ctgtgtgttc ctttacatca tactctccat ggtagagtgt aggggatggg cgccatgtct

9541 ccctagtacg tttgaccttg ggattctttg tctgtgaaca tctttgggtt ctagtgttca

9601 gcagcactta gggaggcact gaattcagtg tacctttggt ctagcctcag ccctgattct

9661 gttctgcggt gggccctggc cttcaagaga acagatatct aaaagttgaa agaaaagatc

9721 ggccgggcgc ggtggctcac gcctgtaatc ccagcacttt gggaggccaa ggcgggtgga

9781 tcacaaggtc aggagatcga gaccatcctg gctaacacgg tgaaaccccg tctctactaa

9841 aaatacaaaa aattagccgg gcgtggtggc gggtgcctgt agtcccagct actcgggagg

9901 ctgaggcagg agaatggcgt gaacccggga ggcagagctt gcagtgagcc gagattgcgc

9961 cactgcactc cagcctgggt gacagagtga gactccgtct caaaaaaaaa aaaaaaaaaa

10021 aaaagaaaaa agaaaagatc aacacatcct gttgtgttat tctgaaagga aagctgtctt

10081 aagaggatca attggtttta gaaaaaacac aatagaatca caaataatcc agaggagaaa

10141 taaaatgtgg aaggtggagg tgacctccag aaaatccagg acagctgctg aaggcaccct

10201 ctgatgagct cggttactca gaagagtgag gatgtgttga aggtatctgc tgtatggagt

10261 ggcaggatga tgtctgtgat tgagaaatat aatcccggcc aggcgaggtg gctcatgcct

10321 gtaatcccag cactttggga ggccgaagcg ggtggatccc ctgaggtcag gagtttgaga

10381 caggagtttg aggtcaggag tttgccaaca tggcaaaacc ccgtctctac taaaaaatac

10441 aaaaaaaatc agctgggcat ggtggtgcgt gcctgtaatt gcagctactt gggaggttga

10501 ggcaggagaa tagcttgaac ccaggaggca gaggttgcag tgagccgaga ccgcgccact

10561 gcactccagc ctgggcaaca gagtgagacc ccatctcaaa aacaacccaa aaaaccaaaa

10621 aacaaacaaa caaaaagaaa tataatccca gtagccccag ctgagctgga ggatggagac

10681 cacttggtag acacttgtgg attatttcct aggctaaatg caaaagctac tgctgaataa

10741 gggacatttt tttccagtcc caggccagta gcgacataga tttcagagtg atctctgtga

10801 gatcctgaag atcctgactg cagaaagtag tgaattgtct tctctcaccc agttttgtga

10861 cattcccttt tcatgccatt aggatctggc cccttcaccg atgttcgtgc agctgtttac

10921 caaccgcagc ctcaccccca gcctcctccc tatggccact gtgtcacaga cagtggtgtg

10981 gtctactctg tggggatgca gtggctgaag acacaaggaa ataagcaaat gctttgcacg

11041 tgcctgggca acggagtcag ctgccaagag acaggtatgc attatctttt tgaagaatag

11101 gactgatgac tttattattt agtttttgaa ggacaataca ttttcaatgt gaaacaataa

11161 aacaaacaag aagcctgtaa tcttaccacc ctgtgataac aattagggtt ggcatttgaa

11221 atagtttctt ccaatctttt taatttatgt attttctttc tggtcatgga tatcatgggt

11281 aaaaatttta ttgtatttat ctgtctaaag tgttgttaca agagagctac tttctgaata

11341 atcatcaatg ttttatattc taaatctcaa atttcagcag ctttgtgatg taaacatctt

11401 ccaataacct aatatatgta ttctgcacta caaacatggt agtcactatg gcaataacaa

11461 ttgctacaca attctccccc agaatagtct catatattaa ttttatggca tagatatagt

11521 cataaatatt atcccaacat ccttaagcag catccttaat tgacctgtat aaatatagct

11581 ttacaaatag agaaactgag gcatggcagc agaagtggtc atgaaggaca tcagcagaag

11641 aactcaggtg tcgttctatc cacagtagac atggattcct gagtaatgca ttttgactga

11701 aattaacgag atgatcatct atactcatag cttcttcctt tgagggcaca agctcagtat

11761 ctcattgaag ccataaataa gcagctgctg gtgggagata aagcatctct gtttactgac

11821 actcttttga ttatgattgt agctgtaacc cagacttacg gtggcaactc aaatggagag

11881 ccatgtgtct taccattcac ctacaatggc aggacgttct actcctgcac cacagaaggg

11941 cgacaggacg gacatctttg gtgcagcaca acttcgaatt atgagcagga ccagaaatac

12001 tctttctgca cagaccacac tggtgagtgt cccaaggggg agccacagaa gtgagaaaaa

12061 ctcactttca tgccctagtt ttatttgcca gcattctagc catttatttt gaacccgccc

12121 aagaagcatc gcttttgttc agtttggact caagagatcg cagcgctcac gtaacagctg

12181 aggattcttc catcttcccc agtactgttg ggaaatgaca ccaagggagt agccttccag

12241 ttcatttgat ttaacacatt gggattatga tgtgattaaa gatacttgta ttttggaatc

12301 agtagatgat cccacagggc tgaggaatac aaaggatgaa tgttttagtg ccttagctta

12361 ttttccagtt aaaacaatgt tttattcaaa gctatcattt aatcttttgt ggggggggtg

12421 ctggggaaat gacagtgaaa gtgggattta aacctgtttt gaaggtgtga aggtaaatat

12481 gctaagaagc ttagaactat attatcagac attttttatt ctgagataga ctgtctgtga

12541 atgagctgca gaaacctggc tctctcagac cagtaattct gtgtacattg gaaagctcag

12601 cggtaatctt ttccttcttt gttgtgtatt gttcctggca gttttggttc agactcgagg

12661 aggaaattcc aatggtgcct tgtgccactt ccccttccta tacaacaacc acaattacac

12721 tgattgcact tctgagggca gaagagacaa catgaagtgg tgtgggacca cacagaacta

12781 tgatgccgac cagaagtttg ggttctgccc catggctggt aagatgaagc ccttgtgggt

12841 tgtcttgttt gacaacaatt tagggagtag agactaaaga ctagtgtcca gtttactccc

12901 atttcattca ttaacacaat tttgagacaa cagaaaactt catgtgaagt gtgtttgtgt

12961 gtgtgtgtgt gtgtgtgtgt gtgtgtgatg ttacatcata tacataagga ttgggaagaa

13021 taattagata attatttata taatttttaa acctcattga catgatttaa tgtcaaaaat

13081 ataattactt atttgtaagt ctggaaatat gaatttgcac aggtttgtct ttgtaaagag

13141 cacacaactg agtagcttac aacatttaat atatgtatga cggctttagt cacagagcta

13201 caatattgac acatggttgt ggtttgatgg gcataagctc tatcacttat taataagtgc

13261 caaagtgact aaaactcaat gttttctaac aggtagggaa tctcactctt tttttaaagg

13321 tccccagttt gtatagatgg cgaacaaatg gaaacgaata ccttttactt gttttcagat

13381 ttcaagaacc ccatagattc cctttaattt tccagttgta gaaacaagag cctgggcggt

13441 aggcactgtc aagtgtgact atgagacaaa gaaattgctt atacttttat ttctttcaac

13501 aaaagaagat gctgagttta gaagaaaaaa cccacttttg cttgtaattc tatatccaaa

13561 cccatagttt ttatttgatc cagaataaac tggaactggg aaaagttatg aagctgtagt

13621 taaatccagg cttctagaac agcaagaacc ctttgtgtgg atgtgtagat attatcttag

13681 tttaacatcc cctaaccctt cctgtaacta ttttctatga cacgtttgga ctacgttttc

13741 tgcctccagg gctcaaaaat tctacccctt cacctgacag cacttagatg tctttgatgc

13801 acacaaagct tcttcccaag tgagaattct taggatgacc aaactgaact gatccttttg

13861 cacacataca tgtttagacc tggtgatcat ttatcaagtg catttcttat ccatttccaa

13921 acagcccacg aggaaatctg cacaaccaat gaaggggtca tgtaccgcat tggagatcag

13981 tgggataagc agcatgacat gggtcacatg atgaggtgca cgtgtgttgg gaatggtcgt

14041 ggggaatgga catgcattgc ctactcgcag cttcgaggta tgctggctga ttaacaaaaa

14101 tatttgagat ggcaaaaggt acagaaaggg acactttttt ttatgaaaac ttgcactatg

14161 ccaaaagcag gggaagaaat atggaatgcc acgtcattca ttagtctact gtgcatggta

14221 agataagcct gaaaggctta gcaggcagcc tgctaagaca agcggcatag caatgctaat

14281 gttctgaaac actcctagca tgtaagtact taggctgagc caaaaagatg gcttcaaaag

14341 taagaatgaa acatttgatc cattcagctt taggctatgc cactggattc atgtctagaa

14401 aagataggat aatttctgta aagaaatgaa gaccttgcta ttctaaaatc agatccttac

14461 agatccagat ttcaggaaac aaatacatag gggactaact ttccttgttc agattagttt

14521 ttctcctttg cacccagcta tataatatga ggaagtattg actttttaaa agtgttttag

14581 ttttccattt ctttgatatg aaaagtaata tttcgggaga accctgagct attaataatc

14641 tatgtggcta gtgcgtagat attggtctga atttgttctc cttttgtggt gtccagtggg

14701 taacaccatc cgggagtaat aattacatgt ggtgttgcag aactgaaaga gaccttaata

14761 acacatagag acctcactct atatagatca aggagctgag acccaaaaag gaaaaagtaa

14821 ttttctcagg atctctcaaa gagtgagcaa cagagttggc ctaatttatt ttagcgttgt

14881 gaatactgtt gacattttat ttcccaaatc taagtatctc ctccccttcc ccctattcca

14941 gagaccagac caccacatca tgctgggtgt tagataaata tgtttaatct tcttcttatt

15001 tatcctaaca agcagatatt taaaggaaat tatcaactaa gcaagaaatt ttcagaaagt

15061 aagacatgta tttgttcaaa tactggcttc tcacaggaaa gtgtatttta ccacattctt

15121 tacttgagca tactgtaacc tctgcaaaag ttacacattt tgggaagaaa aaaatttttt

15181 tggcaaaaat tgtattactg accaaacttt gaaaaaaatg ttattctatg cttgtagaaa

15241 agttatttta gtggaaggtg ttgataatta agtggaagta gttgtatgct ttgagaagca

15301 tacctttttt ctttcatcaa tggaacttta aaaagtttct cactcaccca cctgtttcct

15361 aaacagatca gtgcattgtt gatgacatca cttacaatgt gaacgacaca ttccacaagc

15421 gtcatgaaga ggggcacatg ctgaactgta catgcttcgg tcagggtcgg ggcaggtgga

15481 agtgtgatcc cgtcggtgag tagccctatt tccctagatg agtttgcaca gggggaatgg

15541 ttagcaagtt tcagataaga aaagctatgt gaaatcacat gactgaagtt ggctccagac

15601 tttgatcagt tgcttgcaaa gaactttgca aagtcttctc tctaatactg gaccaaaata

15661 tctcgatatt ggtagtcgtc tggtttttgc tgaatttggt gacaaattta ggcttatttt

15721 aattgaatgg aatttattct tgggtttaga atcataaaga taatccatgc tattaaaagt

15781 attctttcct tttttttttg tttgtttttg tttttgtttt tgtttttttg agagagagtt

15841 tcgctcttgt tgcccaggct ggagtgtatg gcacaatctc ggctcactgc aacctctgct

15901 tcctgggttc aagcaattct gctgcctcag cctcctgagt agctgggatt acaggcatgc

15961 gccaccaggc ccagctaatt ttgtatcttt agtagagatg gggtttctcc atgtgggtca

16021 ggctggtctc aaactcactt ccttaccagc tgtgtaacag catgagcaaa gggtgtaaat

16081 atcacccacc aaaacactct aggttttttt ttggccgccc ttcaaaatag aactaagcaa

16141 atagtgaagg ctgagcctta aaagagctgt gttaccagca ctacaaagtt taaggtgatc

16201 cattactatt tctttaccaa aagagacagg ttgctcactg agaaaacaaa ctgataacat

16261 ccgtttgttt gacgtgagat taccagaact gagagagaag cctgagaggt tttcttagaa

16321 gctgctcagc aggtatactc gtaaagtcta gttcattcat ttaaatgtca aacagtttct

16381 ttaaattttg aagaagtaag gaaaatgaaa ttattgcaga tttttttctt gctatttaaa

16441 tgttaagcca gttatattaa tatgggtaaa aataataact aatatttaaa attaatgtgt

16501 agattatcaa tatacactga aatctaaatc tttacatttt tatttagaaa tattaccttt

16561 tagaaaacta aatattcctc ctaataggta ctttggtttt ttttttacta caaactgtcc

16621 tgtaaggtaa agaatgtgaa caaaatattt ttttaactgc atatatttgt aagaacaatt

16681 gcaaatttct atttaagcta aatgtatgct ctagcaccct gaaattaaat tcgtagttat

16741 aagtcttcaa ggctgtttat cttttccttc catgtatttt agaccaatgc caggattcag

16801 agactgggac gttttatcaa attggagatt catgggagaa gtatgtgcat ggtgtcagat

16861 accagtgcta ctgctatggc cgtggcattg gggagtggca ttgccaacct ttacagacct

16921 atccaagtaa gtagctctat tactgcaagt tgagaactgc caattgggtt ataacaacag

16981 ggcagtgatt attaatgctc tcatgcctaa gttgggggtc tcccctcttt cccacccttt

17041 tctcttgtta ttatctaata atcaattgaa tttttgatta aaataatttt tctctcttcc

17101 tctatcaagt aaaaggtaga gaaggctatg aaaatgtgcc tgtttataat tttacttctt

17161 aactctgtaa aatattctgt taggttaaga cactctggct aatttcatct tatatccata

17221 catggaaata aaaaccacca agtgagttat gctgggagta aaggtttggg gctttatatt

17281 atgattctta acagagaagc tgcatagaga gatggcatga aatgcagcat aaggtacgtg

17341 ttcattcaac atgtcatcta agctcccttt gcatcaaact tttcatttgt ttgatcagtt

17401 gccaccagga acacagtatg ttgggccaag ggttgagtaa cttggtcaac tctctgccca

17461 cacagttcaa acactctcaa atgtttattg ctgggttttt ccaggtcaca aagacatcat

17521 gcctagctgt aggtgtaatt agttcatttg gggggaaaat tgcatttaaa tattcactga

17581 gtgattataa taaaacatgt taataaaaca tgaaaggcta attaaaaggc atcagttatt

17641 tgagcaactg ctgaggtgca aagtctccaa agtcttcact aacgtttgac tgaaaatatg

17701 gcctacattc agaaacaaaa gagtttcagg gtgtcagaat ctgcatgcga cagaaattaa

17761 gattaactct gtgataaaag attcactgtg acagagaaca agctatggga acaacttggc

17821 caaaggtagt gttagccaag cctcattcct ccatttcctc atctgtaata tgggaagatt

17881 gtagctgatt acctttaatg ttccatccta aatactaaac acccagatgc accttttcta

17941 ggaacttgga agattctgct tttcccaacc ctcagacact gtcagtgctg gggaaggtga

18001 cttcactttt gaaggcttat agcacagatt gaccaacctc ctaaattgta tttcttggag

18061 gatttaggtg taggaatcac ttaatttttt gtaacttaat atatatttaa atctgattgt

18121 ggaagtacta taagtatatg aatggtttgt ttgtttatat gatgcaaatg atacttaaat

18181 ggtagaaact tctaaaaaaa tgctctgtgg tttctatatt tatgattgtt attggtgttg

18241 caatttgctg aaaatgattt ccttctgaat gattgagaga gatcttcgtc tctctcaaca

18301 taaggctgtc cacatagctg cttgctggaa gcatttaggt ggacatgttg gagataaaat

18361 ctggaaagga aggaatcctt gagtattgga gtattacatg ttgaccttac tcctactctt

18421 taaaaaggag aacagcaaga tcccactgag catagaggtg atttcggagg gaagagattg

18481 gaatttgacc tcagatatgc tctttggtgc ttatctgtat gtctggtttg ctccgtggcc

18541 tagcacgtag ggctttaaga gtgtggtgag aataagggaa cagcagatta ccaacagatt

18601 gtctctgagt cctgccttgt ttgttcctcc tacagagaac ttggtagagt tgttcaaact

18661 agcaaaagat aagaggcatt tggtttgtca ataagcaact agaaaagcac agatctcagc

18721 aaaataaata gagaaaaaaa ggactgagtc aaaaaatcat aaatgtttaa cttctccaag

18781 gacactacta ttggaaatta ttcatttaga ctttatttga aactaatttt aaaagtgtag

18841 acattgtact atctcctttt tttggtatca tctcaactat tttattgtta gtttattcat

18901 attgaataag agagggagta aagatttcac aatggcgata gctagtatat gccaattaag

18961 ttaatttaaa aagttatacc aactaccagt agcgaaaaag gactgtcaaa agtttaaatc

19021 taaataatgt aaaagatgtc ataattttta acttttctgt ctttaaagga tacatagtat

19081 aagctagagt aattatacgt agtataagct agaatatagg aatttaattg atctaagaaa

19141 taactgacac aaagtctctt tacttcctga acaaaaacat gctaaattcc atgctgttca

19201 gtccatttcc tttaaaggtg ggctatgcca cagggctaga ttttaaaacg tggaatttca

19261 caccagtgct cgaaatctta tgaaagcaaa agggacctct gtagttgtac tccactttgg

19321 cttgagtaaa aaccactgct gtaccctttt tctttccttt ccctgcccat ttttatcctc

19381 ctccttgtgt ccttgtggac atagaaatat gattaggctt agaggtgaac agtaaaggtc

19441 atttatgtta tcttttcaaa acttaataga catttatcca tcataagctt gtcccccctc

19501 aaaatcatga ttgacaagac taaataaagt gtatatcagg tgtctcttta tggaggaaat

19561 tgtagtagga tttatttaaa ggatcaatat ttaaatagcc ctatgccaat tatcataaat

19621 aattaaggac atgatattcc tagctttcct gatttacatg gaagtacgtt aaatagtcac

19681 atctccaaaa tttttcttga atagttgtct ttaaaaatgt gtttacattt gtaaggatct

19741 tcataaacag aaggggtaat tcaaagatag gcatggaatt gacttatgcc aattgattaa

19801 aacaaacatc ctgtgtgttc tgtgaaatac atacaacttt aaaatgaaaa actcataatt

19861 ttatgcatga attttggtgt tcatgtggct tggaaatatg tgcattaaat ggaattaaga

19921 ttcaaagtat ttgctaatct tcaaccaact ttgaattgtt actggtgtgg aagtgagcat

19981 attgttttag aatttctgaa tctacatgga tatccaagtt atatattttt tctgctacag

20041 aaagctttgt tttccaagag aatttaatgg cttagataat aaagtttgaa aatcaatgta

20101 ttttttttcc tagaagcttt cagaaaactt aaatctgtta ataatctggt gaagtgcttt

20161 attacacata caaaattttg ctctgtttga cagagtgtca gttagaaatt cttgaaaagg

20221 ttactataag acacaatttt tatttctagt aatttaaaca ttgactgaca tcataaagat

20281 agtgttttaa gaaaagatag ttttctgttc tgcaagcata aattttctag ctatttcatt

20341 attatcttaa atggagttaa cactacttag aaattgatgc tacttccctt attttccttt

20401 tattttaaac aaaagaacca aaaccatact ttaaattttg ttgataacag tgatatacat

20461 caaggtgtag aaatactgaa tttagtatac acttcataaa gtcatttctg ttgacaactg

20521 attttggaaa aaaaataaat ttaatcactt aataatttga tggatcaatg gtgtgatttg

20581 ggagtaaact tcttgaataa aaataaaact tgatgttttt tccaaactga tagactgctt

20641 agactgatga gaaataaaac taggtcttta attattacct ttgctatttg tctaagattc

20701 tacccccatt tagaaatgtg tttgttttac atcatctcat atggcctttg gaatgttgtt

20761 tccttcttag gtagtagatt cattcttgat gaaaaccttc caaaattaga atttgcttta

20821 aaagaggctc aatacaataa gtaaatgaag tctgtgttct gtattacatt atttttgtag

20881 gatcgtttca caattttctc acaattctta ctttgatagt agagaaaata aaagcaagcc

20941 aagagacttt tttttaaaaa tatattttat ttgctataaa caactatgat ttgcatttct

21001 catgtgaaga ataataatta tcggctaaaa atggatttgc tgcattgatt ttcaacgtaa

21061 atatttaaaa gattaatgcc agataatttt attatactca catttaacga caagcaaagc

21121 tgtttataat gatgactgtc catgtacaca gttttaagtt gaagtgagtg aatattcaag

21181 ataattaaat gctacatttt cattttcagg ctcaagtggt cctgtcgaag tatttatcac

21241 tgagactccg agtcagccca actcccaccc catccagtgg aatgcaccac agccatctca

21301 catttccaag tacattctca ggtggagacc tgtgagtatc ccacccagaa accttggata

21361 ctgagtctcc taatcttatc aattctgatg gtttcttttt ttcccagctt ttgagccaac

21421 aactctgatt aactattcct atagcattta ctatatttgt ttagtgaaca aacaatatgt

21481 ggtcaattaa attgacttgt agactgaggg gattttggtt ttggttttgg gttttgtttt

21541 tttgcggtgg gggggctggt atttggaaga atttagctct ttatgttaca gaaatctttt

21601 ttgcaaggac ttagaaatga taatgcttaa gattgttctt gcccaatgtg ggaagagaat

21661 ctaaggtttt tatatgtctt gcaacctcat caaaggaaaa ttactggcat cattttcata

21721 atttgaaaaa aaaagccaaa ttaatatatt tcttttttga ttcacttttt aagtgatcat

21781 ttttaaaact ttacttttga cccactgaat ttatttagat agaaggaaaa gagatgatgg

21841 gagggaagtt tagataaagg atggaagttg gttttattta aacaatagcc ctgtgatttc

21901 ctaatgagaa gtgactagaa attgaagaaa ccaaataagg aggatattgg tcaatttagc

21961 tttagtttct cttactctct caagcctgcc ctgtttaact ccaaagttca tggctcataa

22021 tttgagaaac actgttttaa acacaggaga aaaaaatgtc cattttaaat catagctatt

22081 gaattctaca attacaaaga aacaaacaaa caaaatttga ccaacccagg cggttaaatt

22141 taaactcttc aggaaaaatt taagctgtta aaattattct ttttctaaat ttctaaagtg

22201 gagggacaga atttttcaga tttaaaaggg cctcctaggt gcccagaaaa ttagtggaaa

22261 gaaccacgtc tagacgcatc tttgatgtgt cagagttcca aggataaaaa gaaactttta

22321 aagtcttcta tactcagcca ggttatcaat caaatatgag ggcaaaataa tattttcaga

22381 cagattttag gcagtttatc ttccatatat ccttttcttt aagggtattt gtagatacac

22441 tccagaaaaa caagagtgaa atatgaagga agttgtgggg tccagcaaac agtgcttcca

22501 aatcagaccc ctgatagagg tggaaaactt tgcaatgcaa caactgcgta gctggcttag

22561 aggacagcca atacagatgg aacagaaaga tgaggatggg attgagggat cagggattga

22621 ggtctccaag aataaaaagg gacttcatgg aaaaagtagg cttgtggata attaatcaca

22681 ggggcaaata atgcagttaa aataacaaca tgacaatcag gtggaggaat gtataataaa

22741 cccaaatgtg gctgggtaga gtggctcaca cctgtaatcc cagcactttg ggaggccaag

22801 ccgggcagat tacctgaggt caggagttcg agaccagctt ggccaacatg gcgaaacccc

22861 gtctctacta aaaatacaaa aattagccag gcttgggggc gcacgcctgt agtcccagct

22921 cctcaggagc tgaggtagga gaatcacttg aacccaggag gcaaaggttg cagggagttg

22981 agccaagatc gcgccattgc accctagcct gggcaacaga gcgagattct gtttcaaaaa

23041 acccccaagt gtattataag gcaataattc ctatacgaag caaactaaaa tgcagcaata

23101 ttaaggtata aaaacaaaga ggaataattc cattgaacct tgattctgga aactttgatc

23161 cacccagcag tcatgatgtt agactcattg aaaagaatgt atttctaatg catgatgcga

23221 tcggtctata gatgtgtcat ggaaacttgg ttgcaacttc aagacaaaat aaaaagtaaa

23281 catttacatg aaaaatggtg gatatggaag gtggagaaga gaggagataa cagctttatc

23341 tttcaaaata gagaattgag agatggtacc aaaagctgat gaagtaaaaa aaaaaaaaaa

23401 aaaaaaagat acttaatata atactttaaa ttacaaatat aaacacaaga agaacaaata

23461 taatgataca aatgtcagac actgggaatg tccaagattc tggaaggaaa gggtggtatt

23521 attgagctaa atcctcaact ttgtctgggc acagtggcta aaaattagcc gggcatggta

23581 gcatgcacct gtagtcccag ctacttggga ggctgaggcg gaaggatcgc ttgagcttga

23641 gaggcggaag ttgcagtgag ccaagatggc actactgcac tccagcctgg gagacagaga

23701 aagaccctgt gtcaacataa ataaatatat aaataaatca tcaagtctca tattaaagac

23761 tctgtaaata tgacttattg ttgacaaatg aaacaaatag aggtgtaagc atgttgtcta

23821 catggaggca agaccagaat aatagaaaat ggaaacagat tcccttaaag aggggaatcg

23881 tgtctttctc attggctcaa tgtagtctcc gtagagtcta gaatgcttca gcacctggca

23941 cactgcttaa caaatggtga atgaaaaaaa aaaaaagaaa agtcattctt tttcttcttt

24001 caccctatgt ccataatctg gccatttgca gaacttgatg tccagtgatc gaaatcaaca

24061 gcatcagtgc atccaatatc ttctagtctc tcatcttctt attacatcat taattttatt

24121 tactttaaaa ttaaggatat ccaaagtatt atgtgagacc attgcaatgg gagacttaaa

24181 agtggtataa aatgtacttt gggccaggcg cagtggctca cgcctgtaat cccagcactt

24241 tgggaggcca aggtgggcgg atcacgaggt caagagatcg agagcatcct ggccaacatg

24301 gtgaaacccc gtctctatta aaaatacaaa aaaattagct gggcatggtg gcgcatacct

24361 gtaatcccag ctactcggga agctgaggca ggagaatcgc ttgaaaccag aaggcggagg

24421 ttgcagtgag ccaggatcac gccactgcac tccagcctgg gcaacaagag cgaaactcca

24481 tctcaaaaaa aaaaaaaaaa aagtacattg aattggaaag tcttcaaaaa gcagcagtga

24541 tgaatttttt gagattttta acaattacaa aaattcaggg ttttttctaa tggatgccac

24601 ctgagacttt attttctgtt attttcttgt aataactaac caaacaagct catgttgaaa

24661 aatgattact aaatttgagc taattgcaat gactggtttc aaaattttcc acagtgtatt

24721 tgagttaaaa tttcactgtg aagagtacta cgatcactct cgcttattcc aaaaatataa

24781 atggacactt gagtatttga attattgagg aaatggttga ctgggtaatt tttaaaaatc

24841 actgggcaca aaaaatatat tttgacttat attagtttag agtatttaca cttgaaagag

24901 tctcatcttt tctgaagggt gtttctttca tacacatttt attgcactga gttttgtgac

24961 ccatggcata ttaatgaagc tgaacaggat gtgaaatata aactggaagc aaaagattaa

25021 ataaaccaaa attgcatttt ttctgtagtc ttgtccaaaa ttgggtaacc acttctgatg

25081 gggtagctca tatccaagaa tgagtcacaa aaccagactc gttgaacctg gtatatgatg

25141 agtcacaaag caacattctg cctttgtttt ttcaggacaa gaaacttgaa ttgtatccca

25201 ctgagttaaa agataaaata tatggcattg gcatttctgt acttcagaga ggaatatatc

25261 tgtttgtggt aggaataaaa aataagtgag agaggcaaag cttaggttca tcatattatg

25321 ttactgatat aacacaatta acttggtaaa agtgaaggtg tgggtgggcg tggtggctca

25381 cgcttgtaat cccagcactt tgggagtccg aggcgggtgg atcacctgag gtcgggagtt

25441 cgagaccagc ctgaccaaca tggagaaacc ccatctctac taaaaatata aaattagcca

25501 ggcatggtgg cacacgcctc taatcccagc tactcgggag gctgaggcag gagaagcgct

25561 taaacctggg aggtggagtt tgcagtgagc tgagattgag ccactgcact ctagcctggg

25621 caacaagagc aaaactctgt ctcaaaaaaa aaaaaaaaaa gtgaaggtgc ccagtgtctg

25681 caactatgtc accccgggca tatcacatat cactctgttt ttccgtctgt aaaacgggag

25741 caacaatgcc attgccttat catcagaagg atatggagac taaatgggag aatgtaggta

25801 aacagcacag agtatgggta tcagtaagta aactgcagca gtttgttgat gttaacaata

25861 gttagcatta tatctaacta tatctaacga tataaccatt gggaatccag ttttccatga

25921 ttttcctcta gaatggagct gcctaagtcc tgcttaagtc atttttcttt gaagattact

25981 gaacatcatc ttcaaatgtt catccttgta aacacgtgtg tgtgtgtgtg tgtgtgttaa

26041 tttaaatttt cagaaaaatt ctgtaggccg ttggaaggaa gctaccatac caggccactt

26101 aaactcctac accatcaaag gcctgaagcc tggtgtggta tacgagggcc agctcatcag

26161 catccagcag tacggccacc aagaagtgac tcgctttgac ttcaccacca ccagcaccag

26221 cacacctgtg accagtatgt acacaaccac cctcatgcct cctacccccg aggttcctag

26281 agctaggctc tcctgaggca atgctttcct tctcaattca tattcttcca ggaggggcac

26341 caacgttttt taaaatgatg ttggcgacga ggacggtaaa ttttctagat gactgaaggc

26401 tgactttccc ctttctgtga ctctctaggc aacaccgtga caggagagac gactcccttt

26461 tctcctcttg tggccacttc tgaatctgtg accgaaatca cagccagtag ctttgtggtc

26521 tcctgggtct cagcttccga caccgtgtcg ggattccggg tggaatatga gctgagtgag

26581 gagggagatg agccacagta cctgggtaag ctcaatatgt cgctcaagac aggttcaggg

26641 cagctgctgg aaaactctcc ttgtgggggt gggtggcctc taggcaggtg gtatctgtgg

26701 tttggaactg gttgacagct cagactgaac aaaccaccct ctggcatgag gaagggaagg

26761 actgactctt tctaagaagt ggccgggttt ttcccaagcc actgtcacat gttcctggtc

26821 cctgatgcca gctgcatcat gcgcctacct gtgcacaagt tcctacagca aaagctgtgt

26881 tcttggtgga agtaattacc aggactgcag ctgacaatgt gagcacagta cggtcactca

26941 tacttttcaa attgttatgg tgaggggcct ttaaaaaact tcattggcgc actgaagtgt

27001 gtgccatcgt aagcactgag ttcagtgaat ttgaattctt ataaagtgaa cacaccacaa

27061 gaccagcacc cagatcaagg aaaagaatat ctctctaccc tcaccccatc atcttccgcc

27121 aataatcact accctgactc ttactgcaga gagctatttt taaaaatttg gcattcgatt

27181 acaaaaatta tacgtcttta gaaaaaaagt tggaaaaatg aaaggaagaa ggaggaggag

27241 gagaaaggag aaagacaaga agtggtgttt tcccatagta cattaataat cacagattag

27301 gtgggtgcgg tggctaactc ctataatctc agcacttcgg gaagctgagg ctggtggatc

27361 acttgaggcc aggagttcga gatcagcctg gccaacatgg tgaacctcca tctctaataa

27421 aaatgcaaaa aaaatagccg ggtgtggtgg tgtgcacctg taatcccagc tactcgggag

27481 gccgaggcag aagaattgct tgaaccggaa aggtagagga tgcagtgagc tgaaattgtg

27541 ccactgcact ccagcctggg caacacagca agactctgtg taaaaataat aataataatc

27601 actgattagc tattagcaca ttaccttcta gtcgcttttc cctatgaata tataattctt

27661 aaaatatcct tcttataagc tgtagagtca tttgagggac tagtttgctc tgattagtta

27721 ccttttcttt tcatttcaaa gatcttccaa gcacagccac ttctgtgaac atccctgacc

27781 tgcttcctgg ccgaaaatac attgtaaatg tctatcagat atctgaggat ggggagcaga

27841 gtttgatcct gtctacttca caaacaacag gtacatgtgt gctacatagt gttaaaagaa

27901 tctttttctg taaaacacag gcctgtagta gcacttcctg actgtttgcc ccactttctt

27961 tctttcttag cgcctgatgc ccctcctgac acgactgtgg accaagttga tgacacctca

28021 attgttgttc gctggagcag accccaggct cccatcacag gtcagctaag cgtccccctc

28081 tttggctgct atgttaatct taatgacatc agcagggagg gcgcagattc tgactgcgga

28141 cctgcatatc actttaaatc tccaatataa tttatgggag aggggtttgt gtgtgtgtgt

28201 gtgtgtgtgt gtggcggggg tgggggagtt attttctatg gcacatttcc ccttgaaacc

28261 atttcaccaa ctcccttata cacacacacc acaacataca cacaacctgt aaagccagct

28321 cattggctta ttaaagcaag tgttcccagg gttgaagagg tgtaatttcc tgaaaacgtt

28381 gctctaagat ttatccttaa ggagaaagct gagctgtcgt cttagctcat taggtgattc

28441 aactgcctca tcactgaagt tccaaaaaga cacacacagt gctagacaac tctgcttagg

28501 ctggttcatt aattgcttcc ctcgtctgga gctcaaagag gaaaaatcag cttaacatga

28561 atattttcac ctaatggcat ctctaattga catttattaa ggatgtcagg tcttcaagga

28621 tgacatttat tattaaaaag gttcgcatga ctgttcttat tttatcttcg tgctgaatag

28681 tcattattag aagaagtggc aatattcaaa gagtcaaaaa gtatcactgg ctcttcacta

28741 atcaagcaag atgctaaggg atattagaaa agggaggatt tatggtgttt ctaagcctct

28801 gtctcaaaga aaacagtgca tcttactttt gctcatgaat ctgcagggta cagaatagtc

28861 tattcgccat cagtagaagg tagcagcaca gaactcaacc ttcctgaaac tgcaaactcc

28921 gtcaccctca gtgacttgca acctggtgtt cagtataaca tcactatcta tgctgtggaa

28981 gaaaatcaag aaagtacacc tgttgtcatt caacaagaaa ccactggcac cccacgctca

29041 ggtaactttt ttaagaagac acttcctatg ttatcttatc aggattgttc ctgaaggagg

29101 gttgttttgt ctctgtcaac agtcctctca ttcaagaaat cttatatatt agtttttccc

29161 taaacttctg atatttagct gaaatgtcat aagtaactta tcaaagctgg ctactggcct

29221 ttctgattaa aaactgacac cataacgtcc atctacaaat tttcccctag aagcttaagg

29281 gtcatttcat ttttgattct taagtattat aaaatgattc agtaaaacaa aaactgttac

29341 attattttct gcagttattt caaaggcttt ttctaaaaaa ttttttagta tcttttctta

29401 taaccctctc cccccaccac catatgacac ttcatatgct ggtaactcac ttattaccta

29461 ttttagataa aaggttcaaa tgtcataatt taagcactat gactggacca tgaaaatgtg

29521 atctgattaa gagacaaaac ttagcaaaac tctcgaatga gaggctcaat gaactgccta

29581 acatatccaa gaaactggct tcttaaaagt aatcttcagc agagaatgtg aatgaccagt

29641 tgactcttgt ctgtcagata cagtgccctc tcccagggac ctgcagtttg tggaagtgac

29701 agacgtgaag gtcaccatca tgtggacacc gcctgagagt gcagtgaccg gctaccgtgt

29761 ggatgtgatc cccgtcaacc tgcctggcga gcacgggcag aggctgccca tcagcaggaa

29821 cacctttgca gaagtcaccg ggctgtcccc tggggtcacc tattacttca aagtctttgc

29881 agtgagccat gggagggaga gcaagcctct gactgctcaa cagacaacca gtatgtcttc

29941 tcctatctct atctcccctc caaattctcc accctcactt gcagcctgtg agaaagtgca

30001 gtaaaccatt cactcagagg tgtatggctt agagagaggg aaatacccag ccggcaaggg

30061 aatgcatagt gaacacaaag cacattaaac ttgaaaacaa aactcagaca agctccatgg

30121 atgctaagtg gtaacccatt tctaaaatac atgtaccagc tgaagggtac taagagggga

30181 gaactgaaga gaatctaatt tgagtgcatt tttcgtgtaa ctaaatatat ctagatcaaa

30241 gttaaaatgc aggatcataa cacttagagt agaattcatt taacaatagc aattgtcaag

30301 tgtctagtat tactagccac cagcttatct gctcagtttt tacaagcatt attctcatat

30361 ttactctttg ttttgacctt aggaaggaag gtcttattat tattatttta tttatttatt

30421 tttttgagat ggagtctcgc tctgtcgcca gggctggagt acagtggcac catctcagct

30481 cactggaacc tctaccacct gggttcaagc aattctcctg cctcagcctc ccgagtagct

30541 gggactacag gcgtgtgcca ccatgccctg ctaatttttg tgtttttagt agaaatgggt

30601 ttcgctgtgt tggccaagct ggtctgaaac tcctgacctc aagtgatcca cccactttgg

30661 cctcccaaag tgctgggatt acaggcgtga gccatcgtgc ccagccggaa ggtcttacta

30721 gtatccgtat tgaatactta aagaaactga ggctttaaaa aagttctgca acttgtaggg

30781 tcacagagat aggaagggat agagctggct ctataaccta tgtccgaagc ccatgctctc

30841 aattattata ctcgactgcc tcttaaagat ttcctctatt tgaaaggtaa tttaaatttc

30901 ggtgggaaaa ctgctggtta ttattctcaa gaataaactc cacaacttat gtgattctga

30961 tagtgcaaac tcaccagtat cctaccatga atctgaggat acgttatcat tactgtaatt

31021 actgtctaat ctgaaccatg tgaaaataac ttttatttct ctagcaaagg gctattcaca

31081 gaatattgct tttgacccat agagagcttc ttcttgctgt cattttagga ggcatatccc

31141 tttttcctta atctgtttgg ctcagagcta actgtgaact tcagaagtgt ttgttttgcc

31201 tttttaaaaa taccattgct ttaatgtaac tataatttct gagactgatg cgaaagtctt

31261 gctggaaaat tagacttccc aaaggatcac agtcaagcaa aatggttcca caatttctca

31321 tgactggcag agttttggca aagttttgtg tagcactcaa tctcttactg gctcagtttt

31381 tccagggttt tgactttcac atagttacaa ccttgaggag agaaaactta gacattcaat

31441 caagtttcag gacttgagtt atgatcatta ctgatctaaa tatttcttgg catgtttcat

31501 cttttttcct agaactggat gctcccacta acctccagtt tgtcaatgaa actgattcta

31561 ctgtcctggt gagatggact ccacctcggg cccagataac aggataccga ctgaccgtgg

31621 gccttacccg aagaggacag cccaggcagt acaatgtggg tccctctgtc tccaagtacc

31681 cactgaggaa tctgcagcct gcatctgagt acaccgtatc cctcgtggcc ataaagggca

31741 accaagagag ccccaaagcc actggagtct ttaccacacg taagctgaaa attaagtgcc

31801 ttttcttaac tatatttaca ttctctattc ttcatgcttt aaaacaaaac aaaacaaaac

31861 aaaaaaaaca ttaaaaaatt agtacataat ttaaatcagt gatactaaaa atgtgctcca

31921 tagactggct gctggcccgt taattgtttg ctgctagtct gcaacaagaa aaacggttgt

31981 gccagaacgt aaatcacaaa gcacactgct tagtacagct gagaattttt ctgaagccag

32041 attttcttga tgaaggaagc agtgtgttaa tttgcacaca ttgccaagct cgctctttcc

32101 tctagggcca gcactttgag taacatgggt ttaaagcagt ctgttattag aaaaattaaa

32161 ttcgattaca ttaaatgaat ttaccaaaca ctagttaacg caagaaaaaa ttagcaccta

32221 tgtctacatt ctattacttt gggcattgaa tagtaactat aaatgcagaa taaaaatatc

32281 tatggattga atgggaacca actaattgaa catgaagcca aggaaatgat ttctttatga

32341 gtgttggctg cagaagatta aagtactttt gcagacggaa tcgctctttt cttaaattac

32401 tcttgaaatt cctcagagga gaaaaatact aacaataatt tttggtcatg tctatccttt

32461 tgctcaacat tttaaaggaa gtggtcttaa atctcccaca tatctacatc acaataacaa

32521 cctctattca caaaccgatt cctattaaat acatttccat ttacattaca gagaattatg

32581 agactcctta tttctagctg aacatcattt gttattttca actcgacatt ttgaattata

32641 gaagcaccta acataagtac tttttcagca tatattctaa ccatggacta gtttgcaatt

32701 ttctaagagc tttcaacaaa tgttactctt cgactaattt aaaagtatgg atgttaaaaa

32761 gcattcaaaa agtccataca agcctagttt gtaaataact atggaattga tttcccaaag

32821 aaaatacaaa cttttcccca taagaattca tactttaaga aaaacttact tccatttaaa

32881 tttactgtat gaagtttggc tcatgaaggc tttttcctaa ataatagtta atcgtaagca

32941 agtaaaattc acttttaatt tgcaaataag cttacttgaa aatttggcta aaattttaca

33001 cggttctaag atagtctaag atctactctc atgaaattaa tgtctttata tttcttgtaa

33061 atattcattt cttataaatg tccttcagtg aattagaatg gagatttcag tgaatgcgcc

33121 ctttcagtag atgtcgtctt ttactaaaat gtagaattct atagttgtct tgttcattcc

33181 ttaacatgag acatatttta tgtagtttct tttgttgaac acagtgctta taaaagaaaa

33241 agcattttta atgatgctaa caataattaa gggaaggtgg tgggccaaga tatttcaagt

33301 acttctgaag actgatatat tggatatatt attttatgct ttgcataata cattcatata

33361 aaatataatg attttaactg aagtactgat agccaaaact aattttatta gataaaggtt

33421 aacatagtgt ctggaacatt gtatgctttc aaaaactatt tggtgaatag ataattgaga

33481 aaggaataat aataaaaaca gctaaatgaa gcttatattt aaataaacta atgtaagcag

33541 ggtattctac aactccattt gaactttaag cactccctaa ggctgtaaac atccacaagg

33601 cttgcatgtt tttgaaatta ctaaatttct gtagtttttt actatcttac taagctgaat

33661 tctgggagta acttttctga gttttataac ttgtgctaaa ttcttaagag caaatgtgag

33721 aaaagttagg ggaaaaagct gtttctgggg aaaattcagc ttagtcttat attgataggg

33781 caaattttat ttctttaaca gctgggctgt tcttccctaa caagacctgc cagaacccat

33841 agctcacact tagaatcaca tcctttgttt gacatcgttt gtgggtttgt ggtttggtga

33901 ttttcccata atggccttcc caggcagaga gcatcatctt aaacttggga agactctagg

33961 tggctggctc aagcaataga aaatacctag tcttaaagcc caggacagtt gaggcgaata

34021 taatttgtaa aaaaagttgt ggttttcaca gatgttcagt gaaagaaact gactgttctc

34081 tgaattgttt tttgtgggcc attaaaaatg gtcacacagg gctggccatg gcattttggc

34141 acagtcacca gcagtcaagt ggtgtataat ttcagaggta ctaaaaggca tcgggtcacc

34201 catcccagtc atgtcccccc accccccacc aaacaccatc aaaataataa catacataga

34261 tgaaacagcc cactaaacca gtagtacctc aattcagcca ttagagctat cattatgaag

34321 tggcccacac atatatttgg ctttttctca cacaatattt ttaaaataaa aataaaaaat

34381 ggctccttag aatccagact gaaaaaaaaa tgcgagaatt gcgatgttga ttctaaattc

34441 ccacatggca aagaaaaaaa aattgactgg agttgagttg agactggagt ctgaacactt

34501 tttaatccct gtttgtataa ctctggagac taattctttg tcttgccagc cattcataat

34561 ttagtataaa tgcattcaga ggttttttcc caatgggaac aaaatttgat tgagatgtaa

34621 agagaggaag aattgtggag atgtcaaaca tgtgaccaga gctatgaaca cacttgatac

34681 ccttgaactt aaacttaccc aactcaaaat atctggcctt ctgcgatctt acctttctac

34741 atttataata agatttgcaa ggttgttgga aatctctatc ttaactttat atatacatgc

34801 ctctttcttt ctttttcttt cctttccttt ccctttcttt ctttctcttt ccttccttcc

34861 ttcctttctc tctctctctt ttcttccttt cctttccttt ccttcctttc tttcttcctt

34921 tctttctttc tttctttctt tctttctttc tttctttctt tctttctttt ctttctttct

34981 ctctcactct ctctctcttt ctttctttct tatttttggt gctaaaaccc aaaacaaatc

35041 ttttatttaa aaataagatt tttttttttt ttggccaggt gtgatggctc atgcctgtaa

35101 ttccagcact ttgggaggcc gaggtgggta gatcacctaa ggtcaggagt tcgagactgg

35161 cctggccaac atagtgaaac cccatcttta ctaaaaatac aaaaattagc tggatgtggt

35221 ggtgggcaac tgtagtccca gctacttggg agtctgaggc aggagaatca cttgaaccca

35281 ggaggctgag gttacagtga gatgagattg cgccactgca ctccagcctg ggtgacagag

35341 caagagtcca tctcaaaaaa aaaattgttt aagtaaaatt ttattttctt tctttttttt

35401 tttttttttt ttttttgaga tggagccttg ctctctcacc ctggctggag tgcagtggtg

35461 tgatcttggc tcactgaaac ctccatctcc caggttcagg tgattctcct gcctcagctt

35521 cccaagcagc taggattaca ggcatccacc atcacacctg gctaattttt atatttttag

35581 cagagacaag gtttcaccat gttggccagg ttggtcttga actcctggcc tcaagtgatt

35641 cacctacatc agcctcccaa agtgctggga ttacaggcat gagccactgc gcctggccaa

35701 agtaatattt tcaataagaa aaataacagt gatgtcagga tgctagaaaa tgcaaaataa

35761 atttgttata attcctgatt ctggtgataa aatactaaat ttttgcagta ttattctgaa

35821 agaataatca cgtatttaaa gtacaaatct ttagacttca aagtgcatcc atggtggcag

35881 attttgttta acttttatat agcatctttt attgcaacca aaaatagctg accattattg

35941 tggaataatt cagcgtaaag cttttttttt ttcttttttt gagacggagt ctcattctgt

36001 cacccaggct ggagtgcaat ggcatgatct cggctcactg caacttctat ctccagggtt

36061 caagcgattc ttgtgtctca gcctcccaag aagctgggac tacaggcatg agccaccatg

36121 acagttaatt tttcatattt ttaatagaga cagggtttca ccatgtttgc cagcctggtc

36181 tcgaactcct gacctcaagt gatccaccca cctcggcttc ccaaagtgct gggattacag

36241 tcatgagcca ccgatccccg cccagcataa agctgttttt agatcacctt ctataattta

36301 ccattgttct taaattaatg gttaagaaac aatatgataa tcagtttgtg gtggccagtt

36361 ttacatttta taagggattt tacactgacg atatagggaa atttattgtc atcaaagtca

36421 aatcccaaca atcataaaaa gacatgaatt agattatata atttcaatga aaggaccacc

36481 ttagcaaaca tctaacccct tgttactcag gtatcattca cgggccagca gaattggtat

36541 tatctgtgtt tgtcagaaat gcaaactccc tagttttttg ttggacctcc agaaacagaa

36601 tctgcatttt agccagaccc cagatgatgt gtgtggcaca ttaaagattg agaagcctga

36661 tcctctccca attctcaccc aatgaaaaaa tatgttacat cctctatcca ctgcttggtt

36721 aaactgaggt tctccataaa aatacttgtt atctatatgc tatgcaatca tctgtgagtt

36781 tgagttttga atatgtgcat tgattctctc tcacagtgca gcctgggagc tctattccac

36841 cttacaacac cgaggtgact gagaccacca ttgtgatcac atggacgcct gctccaagaa

36901 ttggttttaa ggtaaactgc agatgttcct aatctctgtg atacagccct gagctgtcct

36961 tgtggttccc atgtagtgga aacagggtgc tcaggagtca ggagacctgg gttttgtcac

37021 ctgcttctgt ccatacatct ttgactacat tgtcagggcc taacagtcct tccctgccta

37081 cctcactgaa ttgttggaag ggtagatgga ggctgcgaaa gtgttttgca aaggataaaa

37141 cattagcacg aagctgctgc ttattgttat cttattttct ctatcctttc ctgcagggaa

37201 ttacatttca aaaaaacatg ggaaaacttt atttgatgtg ttgttctaaa tgagtgtgaa

37261 caagttcaca aaagccagtt tagggagacc agttaaactc agagtcactt aaaaatcgca

37321 ttttcatcca atcagtttca tctccaactg ttcaaagcac tgagggtgaa tctcttaata

37381 gaagttaaga ttaaggtttc cctgtggata tctggattca tcttctttaa agtaatgata

37441 ttagggaagc ggtgaataca aatgaatatg tttaaaagaa ttccattctt tggcatttag

37501 tgtgaagaga gaaatatttg ttatcgctgg aaatcatgac tcaatcccct tgatcgttta

37561 aaaaaataca ccaaagataa agtttgtaaa tggccatatt tatgattatg ctactcaaat

37621 atagaagaac tttctgaaga gtgccagtat accttttaat tcccttaata atgtcatgct

37681 gactttcaga agccttatga tgtgtgaagg atctctctag agttgaacac tattggataa

37741 cagtgttacc taagtttttg aaatagaatc ttaaaaggat tttaaattat gggcatagtt

37801 attctaattc ttctcttgta atgtatcatc ctgcagttga agctatgtac atatctcttc

37861 aaaaggtgtg tttttgcaat acagttgcta caggggctgg tgcctttaaa tggcaactaa

37921 aaggttaatt gaatgtgaat aactcgttaa agggagagct cagacattcc ttctagcaca

37981 cacacagaaa aatagaaatg aactaccatg tgacccacag tccctgtata cgtctggttt

38041 gtaacaagag attctttatc aagcaaaaca gtatgtaatg acatttctct ggaaccttcc

38101 attacaagcc accttaatgc agtttggaag atacctcccc cacctggggg aatttccagc

38161 taaagtatat aaaagagtcc ccaaatcatt tcccaataaa agtacactgt gcagtttctg

38221 aagagtttac actatttaaa gcataatcat agcctcacag cagtaacagt cctctggaaa

38281 tatttgtcct tggtgtttac tttgcattcc ttcctctagc tgggtgtacg accaagccag

38341 ggaggagagg caccacgaga agtgacttca gactcaggaa gcatcgttgt gtccggcttg

38401 actccaggag tagaatacgt ctacaccatc caagtcctga gagatggaca ggaaagagat

38461 gcgccaattg taaacaaagt ggtgacacgt aagaagaatt ttttcccttt tctattagtt

38521 tttaaaactg ttctactttt cgaaaaaagc tagtgtcaat atcacttttt acttatgaga

38581 atggcacagg ggagatatct tatccttact tatattatta atgctattcc tgaatttgga

38641 gtagcaggtt caattcatgc tttatatttt atggcatcag aaatgttctt tccatgtcaa

38701 gaatatttat gaactggcaa gatgaaaata atttcaatag tattgcaaaa tcattaatca

38761 taaagaagtc tttgtcagag aattactgct gtccatcata ttttcataat gtaacctata

38821 tttttatggg agggagggag ggagggaaga agcatggaag agagggaggt agggaaggag

38881 gaaagattac acaggtcaag agctttgtgt cagctttgac ttttaaaatg ttctttctgc

38941 atagcattgt ctccaccaac aaacttgcat ctggaggcaa accctgacac tggagtgctc

39001 acagtctcct gggagaggag caccacccca ggtaagtttg ggatggatca gagggcaagt

39061 atacaccata ccttcccaag acaaagattt tagaaactgt gtttctttca gagaaagaag

39121 ggattcaaat tacaaatgct tagctctcca taaaaactat agcagtacat gatgtacatc

39181 atggagcagc ctgcaggatg ctttaatgca cgttgacttc aatcacagga agcagaacaa

39241 ccttacacta gtctaggggg acaagacaga tcctcacaca gctgtagggc tgaagaaaag

39301 cactgtggaa ggtggctttt gctgagtgca ttaaaaattg ccaaacaaat ggttgaagtc

39361 atattacgta atttcccttt tgtgagttgt tacagagcct aagtttatta ttccctgaag

39421 ttttatgaat gttttgtcat gggttgcacc acaaatattt aagggtgatg aaaggcagaa

39481 ataccttcat tttacagaat aagaaaactg agacttagaa aaaattggcc tactcatagt

39541 cacacatttt aaatgttaca aaactgggat tgtaacttaa gtttgtagac tccttctcac

39601 acccaatgtc acatatttgg aatgtaattt ttttttaatt atattctatg caaactgaaa

39661 attctgatta aggggtttcc tgaccatttt tagagcttta aatgaagcat ttgtctaaat

39721 tccttgttca catatatttg aaaattattt ataaaatgct aaaatgtata aagatagttg

39781 ttaacaatat tcaaacagca tgacgtacta tagcaataac aggaaatttt agatacccat

39841 tacttttgcc aaaaccacat ggaagtctca acaaacctat ggagaaaaat cttaaacaaa

39901 aataaaagtt ccaattaatg ttgattgcat tcttacctca tatacttgtt aatttaaggg

39961 atatgtttag gttattattt agctatttct aattttactg taaaattctt gtgaaatttt

40021 tgtttaaaaa aaagtattat atagttctta tctttgccgg ggcacagagc taaggctatc

40081 atctctaaat ctgattaatg tatgcaaaca cacagaatga aactagctca gaatatctct

40141 tttaatctcc ctctgaagta gagtgatttt ggtaaagttt tcattatctg cggaaacatt

40201 gtttaagcca aagctataca atttccagct gagttgctct gaatttgaaa ctttaagttg

40261 acaatcttcg tgcttgttag cagcaggatc attaatatct cgtctcaatg gcccagccca

40321 cacatatgga tgaccactag caagtgtaat gatctcaata tttatttctc attcagttgg

40381 gtttccttgt atttgccaca ttagtgttta ccctgttcct aatggcaaaa tattctgtca

40441 tctccttgcc ttttataaag tttaatatac tttctcattt taatctgtcc ccacagatct

40501 ctagtcatca ctgtttttat ttgaatgtct ctcatccctc tcaactcttt tactgcccaa

40561 tttctgtgat tcctgaagac ttcaacaatc aatactctct ttttttgttt gttttgtttt

40621 ttttttttga gacagagtct cactctgtca cccaggcttg gagtgcagtg gcgccatctc

40681 aactcactgc aacctccgtc tcctgggctc aagcgattct cgtgcctcag cctccccaag

40741 tagctgggac tacagacatg cgccaccaag cccagctatt ttttagattt ttagtagaga

40801 cagggtttta ccgtgttggc caggctggtc tcgaactccc aacctcaggt gatctgcctg

40861 cctcaacctc ccaatcaata ctctttctag aataagtatc agcacttttg tttctcacct

40921 tttctccttt cttggttctc ttcctataaa tcccatagtt tcagaccttt taaattagga

40981 gagctctctg gggaatgtgc ttaaggtgga gagcgattct atactaggca ggtagaaagg

41041 aatattcctc agctgtcttc aaatgattca ttaaggaaaa gcagggtaca gtgataggac

41101 catgagattt ggaaacaaag aaagctttgg ggaatcactc ccctggttca agatttcctt

41161 taaagtgagg atcttggcgg aggttgaagt gagccaagat cacaccgctg cactccagcc

41221 tgggtgatag agggagactg tctcaaaaaa taaaaataaa aaaataaagt gaggatctta

41281 gtactgcctg aaaggattgt tgcaagcatt gaataacagt gacagtggag tcctcagtaa

41341 atgccaagtc ctgcattccg ccctgtgaat ccatcattgg agtctagtta aatatgctct

41401 ggctcacaga tcctctgtgc aataacttcc cttttctttt ttctccagac attactggtt

41461 atagaattac cacaacccct acaaacggcc agcagggaaa ttctttggaa gaagtggtcc

41521 atgctgatca gagctcctgc acttttgata acctgagtcc cggcctggag tacaatgtca

41581 gtgtttacac tgtcaaggat gacaaggaaa gtgtccctat ctctgatacc atcatcccag

41641 gtaatagaaa aataagctgc tatcctgaga gtgacacttc caataagagt ggggattagc

41701 atcttaatcc ccagatgctt aagggtgtca actatatttg ggatttaatt ccgatctccc

41761 agctgcactt tccaaaacca agaagtcaaa gcagcgattt ggacaaatgc ttgctgttaa

41821 cactgcttta ctgtctgtgc ttcactggga tgctgtgtgt tgcagcgagt atgtaatgga

41881 gtggcagcca tggctttaac tctgtattgt ctgctcacat ggaagtatga ctaaaacact

41941 gtcacgtgtc tgtactcagt actgataggc tcaaagtaat atggtaaatg catcccatca

42001 gtacatttct gcccgatttt acaatccata tcaatttcca acagctgcct ataaaatagt

42061 tttgtccctg tatgtgagca ctgaaacagc atttggttga cacatctagt ttttcatctt

42121 gcagtttcaa atccttcttt ttgaaaattg gattttaaaa aaaagaagta aaagtcacac

42181 cttcagggtg ttctttcttg tggcttgaaa gacaacattg caaaggcctg tctaaggata

42241 ggcttgtttg tccattgggt tataacataa tgaaagcatt ggacagatcg tgtccccctt

42301 tggactcttc agtagaatgc ttttactaac gctaattaca tgttttgatt atgaatgaac

42361 taaaatagtg gcaatggcct taaccttagg cctgtctttc ctcagcctga atgtgctttt

42421 gaatggcaca tttcacacca tacattcata atgcattagc gttatggcca tgatgttgtc

42481 atgagttttg tatgggagaa aaaaaatcaa tttatcaccc atttattatt ttttaacctt

42541 cttcatgcaa gcttattttc tactaaaaca gttttggaat tattaaaagc attgctgata

42601 cttacttcag atattatgtc taggctctaa gaatggtttt gacatcctaa acagccatat

42661 gatttttagg aatctgaaca gttcaaattg taccctttaa ggatgttttc aaaatgtaaa

42721 aaatatatat atatatatat tccctaaaag aatattcctg tttattcttc tagggaagca

42781 aactgttcat gatgcttagg aagtcttttc agagaattta aaacagattg catattacca

42841 tcattgcttt aacattccac caattttact actagtaacc tgatatacac tgctttattt

42901 tttcctcttt ttttccctct attttccttt tgcctccccc tccctttgct ttgtaactca

42961 atagaggtgc cccaactcac tgacctaagc tttgttgata taaccgattc aagcatcggc

43021 ctgaggtgga ccccgctaaa ctcttccacc attattgggt accgcatcac agtagttgcg

43081 gcaggagaag gtatccctat ttttgaagat tttgtggact cctcagtagg atactacaca

43141 gtcacagggc tggagccggg cattgactat gatatcagcg ttatcactct cattaatggc

43201 ggcgagagtg cccctactac actgacacaa caaacgggtg aattttgaaa acttctgcgt

43261 ttgagacata gatggtgttg catgctgcca ccagttactc cggttaaata tggatgtttc

43321 atgggggaag tcagcaattg gccaaagatt cagatagggt ggattggggg gataaggaat

43381 caaatgcatc tgctaaactg attggagaaa aacacatgca agtattcttc agtacactct

43441 catttaaacc acaagtagat ataaagctag agaaatacag atgtctgctc tgttaaatat

43501 aaaatagcaa atgttcattc aatttgaaga cctagaattt ttcgtcttaa ataccaaaca

43561 cgaataccaa attgcgtaag taccaattaa ttataagaaa tatatcacca aaatgtacca

43621 tcatgatctt ccttctaccc tttgataaac tctaccatgc tccttctttg tagctaaaaa

43681 cccatcaaaa tttagggtag agtggatggg cattgttttg aggtaggaga aaagtaaact

43741 tgggagcatt ctaggttttg ttgctgtcac taggtaaaga aacacctctt taaccacagt

43801 ctggggacaa gcatgcaaca ttttaaaggt tctctgctgt gcatgggaaa agaaacatgc

43861 tgagaaccaa tttgcatgaa catgttcact tgtaagtaga attcactgaa tggaactgta

43921 gctctagata tctcacatgg ggggaagttt aggaccctct tgtctttttg tctgtgtgca

43981 tgtatttctt tgtaaagtac tgctatgttt ctctttgctg tgtggcaact taagcctctt

44041 cggcctggga taaaataatc tgcagtggta ttaataatgt acataaagtc aacatatttg

44101 aaagtagatt aaaatttttt ttaaatatat caatgatggc aaaaaggtta aagggggcct

44161 aacagtactg tgtgtagtgt tttattttta acagtagtac actataactt aaaatagact

44221 tagattagac tgtttgcatg attatgattc tgtttccttt atgcatgaaa tattgatttt

44281 acctttccag ctacttcgtt agctttaatt ttaaaattac attaactgag tcttccttct

44341 tgttcgaaac cagctgttcc tcctcccact gacctgcgat tcaccaacat tggtccagac

44401 accatgcgtg tcacctgggc tccaccccca tccattgatt taaccaactt cctggtgcgt

44461 tactcacctg tgaaaaatga ggaagatgtt gcagagttgt caatttctcc ttcagacaat

44521 gcagtggtct taacaagtaa gcagttgaat gtatctgttc cataaatatt aacctagagc

44581 atagcaaatg aattctaaat tctcaagtag gaggagctaa gagcaagaga gctgcaacca

44641 agctacaaac taaactctga attcaatgca cagctccatt aattttgaaa gatgtaatgt

44701 ttgttgctat cttaatatac ttttgatatc tacagcttta aaaaaatcat agtggaaaaa

44761 cacctgcagg aaagttccat gacttcaaac aaattctgct tctaaataag cacgtaaaaa

44821 taagtgaata tcaagagaaa ttatatgact aaatctaaat ctttagagaa aaaaatgaga

44881 actgaaaata gtgtcaccat atgtgcttta ttctcatttt tataaaaaaa gtgtcagcag

44941 ttgattgatt taggatttga atacttagaa aagtgactga ttgtttggtc tagattagaa

45001 tgttgttgtg aagagagtca gaagtttaat ttgtacttca aaaagaatct gttagaagga

45061 tttctcagaa gactgagagc ttagaaaaaa aactgacatt aaataaataa caacaattta

45121 tggaaattgt ctctttctag tcccaaccat tatagaatag acatcttttg ttaaagaata

45181 aaacagtagg ctgcaagatg gtgctgtgtt tcacataaac agtgcttttt attattttca

45241 ctgtaatagt caaatatata acaacagcaa aagattctac ataagggaaa aatagcttac

45301 atttaggtac attaccaagt attagtctga aaacatctac ctttcaaaca taatttagat

45361 aatgaaacac aaagaagagc agctcagcgt gaccataatc ttggtttctt actttgtggc

45421 tgagggcaag aatatcttta tattggcata tccaccaccc cagggctgtt gcttctgttc

45481 tagagcaccc tggaatcact aattacagca tcacccagta tacaagcccc tgcatcacaa

45541 tgtctgtccc ttagccgtag acctgtcaca tgctaatcat gtgttctaag accttattat

45601 aatcctaatg ctacagatga cctcagggta gcccctctcc ctcctagcaa agtcattatt

45661 atcctctttt aaagatgaag caaaccagtg cagtggctct cgcctgtata atcccagcac

45721 tttgggaggc tgaggtgggc caattgcttg agcccaggag ttcgagacca gcctgggcaa

45781 cacagtgaga ccaagtctct acaaaaaata caaaaattag ccgggcatgg tggtgcacac

45841 ctgtggtctc agctatgtag gaagttgagg taggaggatc acctgagcct ggggaggttg

45901 aggctgcagc aagccatgat cgtgccactg cactccagcc tgggtgacag agtgagaccc

45961 tgtctcaaaa aaataaacaa acaaataaac atgaagccgt cgaggtcccc agcagttaag

46021 taaattgcca ctggccagct agtatggtgg aaatggaatc caggcatctg gtcctccatt

46081 ctggcacttt tccaaacttt tgtagggggc tttatagaaa cgctgaaggg caaatggtgt

46141 gcaggggaat gagggtttag tgagtaggag ttccaaattt agaaacctca ctctctgtat

46201 cttttagata tacaaatatt taccatatat tacagttgcc tctagtgttc agtacagtaa

46261 catgctgcac aggctcatag cctaggagca ataggctaga ccacatagct tatagcttag

46321 gtgtgtcaaa ggctctacca tctaggtttt ttgttttgtt ttgttttgtt ttgtttttga

46381 gacggagtct ccctctgttg cccaggatgg agtgcagtgg cacgatctgg gctcactgca

46441 acctccacca ccaaggctca agtgattctc ctgcctcagc ctcccgagta gctgggatta

46501 caggcgtgag ccaccacgcc cagctaattt ttttgtattt ttagtagacg aggggtttca

46561 ccatgttggc caggctggtt tcaaactcct gacctcaagt gatccaccct cctcggcctc

46621 ccaaagtgct gggattacaa gcccatctag gtttgtgtag gtacactcta tgatgcttac

46681 acaataaaat caccaaatca cacacttatc aaaatgtatc tccaccatta agctatgcct

46741 gactgtgtat caaaatggaa gaagaagctg ggcacagtgg ctcacgcctg taatcccagc

46801 actttgagag gccaaggcgg gtggatcaca aggtcaggat ttccagtcca agcctggtta

46861 acacggtgaa gccccgtctc tattaaaaat acaaaaatta gccaggcatg gtggcaggca

46921 cctgtaatcc cagctactca ggagtctgag gcaggagaat cacttgaacc caggaggcag

46981 aggtttcagt gagccaagat cacaccactg cactccggcc tgggcaacag agtaaaacct

47041 cgtcaaaaag aaaaaataat aaaaataaaa aaaaatggaa caagatactc agggatgtat

47101 atttaatttt ttaaaaaata ttctgctctc attttaatat ggcagaaccg attgctttct

47161 aagtgtggct tttttccagt aaaggttaat tattaagacc actagtcctg gcctgggtca

47221 atcccagtat gatcctgggc aagtaaatta aagaagataa cttctctgtg cctcagtttt

47281 tttttgtttt gttttttgtt ttttcattta caaaatggag ataattgtag taaatcaaat

47341 ttttagaggt gataggtttg ttcatttctt gaatgcggtg atggtctccc aagtcacaca

47401 tatgtaaaaa cccatcactt taaagatatg cagtacgttg tatgacaaga aattgctttt

47461 aaaaggagca aactaccttc cagggttgtt gtgaggcata aatggcaatc cacagcacca

47521 cagcaaggat tatcatgtgc cctccagaga catactctca ggtggatgcg agaaatatcc

47581 agctgttgca gcaacttcat cccactcgaa atccagctga gtcacactca caggtggaat

47641 ggagggcttc gagaggccat ggggcaaggt gacccttcct tatcatctaa ttacagacct

47701 tctcaaggtc tgttcactga acacttcgct gtagtgttca cttaggtgta agtaggctat

47761 aggactggac atttggatat ttcatcagtt caaatagtcc tgggcgtgct ttagtttctc

47821 atgcttttga gcagagtttt aaaataagcc ccatttgccc ctacagatct cctgcctggt

47881 acagaatatg tagtgagtgt ctccagtgtc tacgaacaac atgagagcac acctcttaga

47941 ggaagacaga aaacaggtga gtggtgttgg cagtatgact atccagtagc ttttgcctat

48001 caattctgta taacaaatga aatgctactt ctaaaaatac atctccattt tttgttgtca

48061 tggtgtgtgt acctttgtca tcacagtatg attttatcgc tggtctcaaa aactaaaaga

48121 taccttactc aacaatcacc tagactttca gtcactaaca aattaagaaa tttgttgtct

48181 gtccttttaa aaaacatttt ctaagaagat ctttgttatt tagatttagc agacattcct

48241 tttcattagg cagctctgtc taatggctga cccaacactc attgtcatct atttgtcttc

48301 ctttactaag ccagcaagtt tacattttct ttttacttaa taaaatatgc atttactaga

48361 aggaagttga attgaatctc ataaatatta catacttaaa tatgaatgct tttaattttt

48421 tctttcaaaa ggtacacttt agtgtattca ttaatttatt tatagtccac ttgcttccaa

48481 aaaggactta tgatatctta gtttggtttc ttattgaaaa gaactagtaa atgctgtaac

48541 tgaaacagaa atttgctgga agtcccagag actaagtgat ttgaatttgc aacaaactct

48601 gaatttttgt gcatttttga aaaatgcatt tttcaaaact gtcaattcac gaggaattat

48661 cagcattgta atttgtctgg gataatgtct ttagtttcag aaagttttgt gtttggcatc

48721 attaccactc tgttgacata taaatttcct cttgagctta ggaggcttct ctgagagtca

48781 aacatttact ttgagagtgg gcagatcttg ctttacttgg aaggatacac ttacaggata

48841 gaaacacaga atacttgaac actgaagaat ttgaaaatgt caattctcag aagatcttga

48901 acacttatct ccaaatgtga cacagaaact tactgtaata acccctaaaa tctgcttgaa

48961 ttacttagca caagaaaaaa atgaatgctt gagctggcta ttttgaattg agtcaattta

49021 agattttaaa attcatatgt agcttagaat cagtacatct tactctttgg tttatggcaa

49081 atcatggtat tgatgagaca ggaacgaaat gttggatgta cgttaatttc ccctacacct

49141 tcctcacttc ctaaactggt ggtgtctttt cttttttttt tctcttcctc ccccgggtgg

49201 gaaaaacagg tcttgattcc ccaactggca ttgacttttc tgatattact gccaactctt

49261 ttactgtgca ctggattgct cctcgagcca ccatcactgg ctacaggatc cgccatcatc

49321 ccgagcactt cagtgggaga cctcgagaag atcgggtgcc ccactctcgg aattccatca

49381 ccctcaccaa cctcactcca ggcacagagt atgtggtcag catcgttgct cttaatggca

49441 gagaggaaag tcccttattg attggccaac aatcaacagg taacttttct tgtctgcaaa

49501 gaaactcaga agactttcct acccagttgg tagattctgt aaagtagctt gctgttgtct

49561 gtcatcagct ctcaaaaaaa aaaaaaaaaa aaaaaaaaat agatcattgt catggtacat

49621 ggagagggaa gtgagaaaat gtggagaaac atcttcctta gaatatggta aagaagcccg

49681 ggcgtggtgg cagtaaagaa gataattttt ttcctctcaa gaaatttctc acctgatttg

49741 ggtatttatg catttctaat aacacaagtt ttgttgaaaa tgtagaaaat tggccggacg

49801 gggtggctca cgtcagtaat ctcagcactt tgggaggccg aaatgggcag atcacttgag

49861 gtcagaagtt caagaccagc ctggccaaca tagtgaaacc ccatctctac taaatataca

49921 aaaattagca aggtgtggtg gcatgcacct gtaatcccag ctactgggga ggctgaggca

49981 ggagaatctt tgaacctggg aggcgaaggt tgcagtgagc tgagatcagg ccattgcact

50041 ccaacctggg tgacagagca agaccctgtc tcaaaaaaaa aaaaaaaaaa agggccaggc

50101 gcaatggctc acgcttgtaa tcccagcact ttgggaggcc aaggcgggtg gatcacgagg

50161 tcaagagatc gagaccatcc tggccaacat gatgaaacct cgtctctact aaaaatacaa

50221 aaattagctg ggcgtggtgg catgcacctg tagtcccagc tactcaggag gctgaggcag

50281 gagaattgct tgaacccagg aggcggaggt tgcagtaagc caagattgtg tcactgcact

50341 ccagcctggt gacagaggga gactctgtct caaaaaaaaa aaaaaaaaag gtggaaaact

50401 gaacactgtt tcaaagtacc tttaaaaata taattttagg gtaatagtgt cattgttctt

50461 agcagataga ggctgaagta cttacgggaa cagtagcatc atgttatctg tattttagtc

50521 tcaagtcgtc aagccagaga caaataccta agggaagggt atataggtgt tcattgtatt

50581 actttttttt tttttttttt cttcctgaaa tggagtcttg ctctgtcgcc caggctggag

50641 tgcaatggtg ggatcttggc tcactgcaac ctctgcctcc caggctcgag caattctctt

50701 gcctcagcct cccaagtagc tgggactaca ggtgcccgcc accacgcccg gctaattttt

50761 gtatttttag tagagatggg attttaccat gttggccagg ctggttttga actcctgacc

50821 tcaaatgatc cacccgcctc ggcctcccaa agtgctggga ttacaggcgt gagccaccac

50881 gcccggcctt gtatcacttt tttttttttt ttttttttta acttttctgt agttttgaaa

50941 tttttccaaa taaaatgttg agggaaaaaa actcttcccc aaatttgaaa taatcatttt

51001 atcacaattt gaatgggctc tgtaacccct tatcttgaat tcgtcataat ataaaattct

51061 gctaattaca cgtagtattt acatgattgt atggaagaat cattaagaca attatctgga

51121 aaatgaacaa acagtaaatc tgaatattgt ttgaaaatta cggatgtgaa aagtttccct

51181 tttttttcta gtttctgatg ttccgaggga cctggaagtt gttgctgcga cccccaccag

51241 cctactgatc agctgggatg ctcctgctgt cacagtgaga tattacagga tcacttacgg

51301 agagacaggt acagcagtaa aatgctattt tacactctga ttaaatcaga ttctgttgtg

51361 gataacctga aagcccaaca gtgaacaaag aattaaagaa actttggcaa gtccattcaa

51421 cggagccctt gttttttcca agaaaatacg taagatatag atgatataat ttgttctaaa

51481 acccaaataa aaagttgttt atatactaca actagagggg gaacggcaga gctgaggaaa

51541 taaaaggatt gtaaattcac aaacatatta tcagtggtgg aaataagtga tttttatttt

51601 ttcttctctt tacttttctg tattttccaa attttattta aaaggaatgt attctgttaa

51661 aagttttaaa aaggacacaa tgcatgcaat cctgggttga gggcttacct tctcccactt

51721 ctaatgctac tctactactc agtgacattt taaagctgaa atgttaaaac agcgctaact

51781 gtaattttct ctcaatgttt atacacttac caaggtttgc tacatgcata aatacccctt

51841 tctgttcaag atagcgctct ttaaaaggga ataagcaaga agatgtgatt tacatgctgc

51901 tataaatgtg gtaattcaat taatcagtaa tacccaagta gctctaaacc cctcacactc

51961 tgaactaacc ctttttcata caggaggaaa tagccctgtc caggagttca ctgtgcctgg

52021 gagcaagtct acagctacca tcagcggcct taaacctgga gttgattata ccatcactgt

52081 gtatgctgtc actggccgtg gagacagccc cgcaagcagc aagccaattt ccattaatta

52141 ccgaacaggt acaaacttct actctggggt gacaccagct tttacttatt cagatactgt

52201 tttgcaatgt tctcccaagg tatttttcta attgtagaat agattttcct ttttaatgag

52261 caacaacctg cagctagcac ctgcagcgaa cagagttttg agccagataa agaaggaagc

52321 accccaaggg caggaagttc agtcagtttt gtcgatatat tccgcatgtc tgcaatacga

52381 caggcataga gagtgttcag taagtatttg tgggaaaaga atggatgagt tgataaagta

52441 ggaagagaca cctgcttgtg gaatgtagct tctttgtgaa tgaagcaacc atctcaaaaa

52501 taggaaatgg tattgagatg cctgccccat ccctctaaaa gctctctctg tattctttcg

52561 agaagaaata cctttctcat gtaagcgatc attcgaatat gtaccagacc tagagaggag

52621 gacttgtcca atcttgtctc caaggactgg ggcttcactg gtttctccct gcttttattt

52681 gtagaaattg acaaaccatc ccagatgcaa gtgaccgatg ttcaggacaa cagcattagt

52741 gtcaagtggc tgccttcaag ttcccctgtt actggttaca gagtaaccac cactcccaaa

52801 aatggaccag gaccaacaaa aactaaaact gcaggtccag gtaagaatca tctgcatctc

52861 ggccaggtgc ggtggctcac tcctataatc ccagaacttt gggaggctga tgcgggcaga

52921 tcacttgagg ttaggagttc gagaccagcc tggccaatat ggcgaaaccc cgtctgtact

52981 aaaaaataca aaaaaattag ctgggcatgg tggcttgtgc ctgtaatccc agctactcag

53041 gaggctgagg caggagaatg gcttgaagtc tggaggcaga ggttgcagtg agccaagata

53101 gccccactgc actccagcct gggtgacaga gtgagagact ccatctcagg gaaaaaaaaa

53161 aaaaaagagt aatctgcatc tcatatacaa caggatagat ggggtaggac cacctaatat

53221 tcttttttat ataaatggct accttgttgt gagtactatg tatttttttg tcctatgtca

53281 tcattgtccc cattcatgag ttcagggctc aagatcatta tcaacccttt tcacagtaga

53341 agtcttaagt gcatttctgt ttttacatgg atagttctat ttagtgatat ggacatctta

53401 aattactaga ttcacccttc tggttttgtt tatcattcac actaagaaga gataaatggc

53461 ctaactgact ttttcagctc tttttagcta tgttgtcttt gtttttaaat agaatacttg

53521 tgaaattagg atcttaaggc aatttattag agtcaagtta attttcattt tttctgagag

53581 cagtatcact aattgttggg ggcatcatat taagttttag atcttatcct tgagtgtgac

53641 ttcactccca tatggtaatt tgtattagca atgaacaggt ttgtccaaga ggaaatcaaa

53701 gtctgactct ccatattttt gttacaattc tgcaaataaa aattctaggc caccatatgt

53761 ttactaccaa actctagacg ccacttgagg actttatagt ggatgacgtg gatgttgcat

53821 ttgcttttca ctccctttgc agatcaaaca gaaatgacta ttgaaggctt gcagcccaca

53881 gtggagtatg tggttagtgt ctatgctcag aatccaagcg gagagagtca gcctctggtt

53941 cagactgcag taaccagtac gtaaccactg cttggtttcc attttcaaag tcaaattttg

54001 ttcttgggtg tctgaatgcc cacgacatgt cttttgcaat tacacatagg gaaagtgaac

54061 ttgttggtta gtttatgtct tgagctgagc cctttacgaa catctttttt ccttctcagt

54121 gccaagcgag gaatttacag agaaagaagt tgtgaaacca ccatagttag ttgctgtgct

54181 ttgaatttct tttgctcaaa tggcctcagc gaaatcttat ttgcctatag caaatctaca

54241 aaaaattttc ctagaccgtc ttttctacaa ctggatggta aagttgattg aagtgtgcct

54301 catgtagctt tatgtttggg gcatttgaag ggctatggct ggaccagagt gtaatataaa

54361 tgcttaatag agaggggaaa agaagagtgt aagaaccatt atagggctgg gctcacgcct

54421 gtaatcccag cattttggga ggctgaggca ggcggatcac gaggtcagga gttcgagacc

54481 agcctgacca acatggtgaa accccatctc tactaaaaat acaaaaatta gccagtcgcg

54541 gtggcacgtg cctgtaatcc cagctactca ggaggctgag gcagaagaat cacttggacc

54601 caggaggcag aagttgcagt gagccaagat catgcctctg caccccagcc taggtgatag

54661 agtgagactc catctcaaaa aaaaacaaaa caaaacaatt ataacaattt gaatctgaca

54721 ttgcaaatca gctttaccac ttccaaggta tagaaaatcc aggtctatga gactaacatc

54781 acattgtaaa aatcaaatcg tggtagaata tctttaaatt aatataaata catccccatt

54841 gtggggacat tttgcagggt atctgcttat ctcacataca cctatgtttt aataagtgat

54901 gcaacattgc atattttcta aaccaagaaa aattaagcaa gtgtttaagt gatttttcct

54961 tttgatagtg ggttaattgg acttcatcaa agaaaatggt atctgcaaaa ctgctttgca

55021 tgttataaaa atgcttattt cacaacttgc ttttcacata acctcttacc attaatttgc

55081 ctaacagaca ttgatcgccc taaaggactg gcattcactg atgtggatgt cgattccatc

55141 aaaattgctt gggaaagccc acaggggcaa gtttccaggt acagggtgac ctactcgagc

55201 cctgaggatg gaatccatga gctattccct gcacctgatg gtgaagaaga cactgcagag

55261 ctgcaaggcc tcagaccggg ttctgagtac acagtcagtg tggttgcctt gcacgatgat

55321 atggagagcc agcccctgat tggaacccag tccacaggta tatggttaat tgcaccacca

55381 ggtgcccatg ggagcagcgg ctttatgccc tactgaatga attatgcttc actgggctat

55441 tgattcccgt gtaagggtga aaaagaatta ttaggaaaga tcctctttaa agaggaatgg

55501 taagaaacaa taaaacttag gtgatattta aggaaacaag tctgattaaa agaaattttg

55561 gagtatcctg gcttatacac aagaccataa agcaagacat ttgaagagga tactaaagtt

55621 gtggattatt tcctaagctc tgactccctg tgattaccct cactatgtat aaagaaaaga

55681 agtttggcat tacagagctt acttataaaa aggaacccaa actcgggcat ttcatagcag

55741 catgattctg agcacacgtg ggtaagacct ttcttctctg gttagatatc atatgctggt

55801 gtataattag cttaaatgat tgtgatttag acacctagga aataatcaat agggcaattg

55861 ctttccataa tactttatct tcttgtgctt tatttctgaa gcagagtaga atgctaaaga

55921 tgtatcctag tgacagcata aaccctagag gtgacagtct gtattattgc ttttcgcttc

55981 tcttttctgc ttctgttggg agccagtttt cttcttacgc cgcattacag agagaacgtc

56041 aaatttagca gccatatctg ccatagggtc caaataaaga gacaataaaa acattattct

56101 ctcttttttg gatggaatac tgcgtgaaat ggttatccat acaaagatac tttatgtaga

56161 atagaaaaag gaggccgggt gcagtggctc acacatgtaa tcctagtgct ttgggaggct

56221 aagccgggag cactgattga ggccaggagt tcatgatcag cctgggcaat gaagtgagac

56281 cccgtctcta caaaaaaata tgaaaaaatt agcgaggtgt ggtgacacat gcctgtagtc

56341 ccagctactc aagaggctga ggtagaggat cacttgagcc tacgagttca aggctgcagt

56401 gagctatgat aactccactg cactgccgcc tggatgacac agagagaccg tttctaaatt

56461 aattaattaa caattttaag aaagaaaaag ggccattgct tatttttcca tacaaaagta

56521 aaataaatca taatggccaa taagccaatg taactttttt ttttaaggga aagcaaaact

56581 tgtaaaacct aaaatctctt agagttttgg catttaccca aatgttttca gtgattctga

56641 gaattggtgg atataaaaca catttctcag caaacacttt cttcattttg catcccttac

56701 tgtacgtact ttcttgtact gaatctttgc ttgaccaggg aacccaccta gcccaacaag

56761 aacaatccat tctacttctt ggaactcact ttattttcct tttcccccat ttcctataag

56821 ataacctcta accaatgaca atctcgacag ctattcctgc accaactgac ctgaagttca

56881 ctcaggtcac acccacaagc ctgagcgccc agtggacacc acccaatgtt cagctcactg

56941 gatatcgagt gcgggtgacc cccaaggaga agaccggacc aatgaaagaa atcaaccttg

57001 ctcctgacag ctcatccgtg gttgtatcag gacttatggt aagacatgac cgttgttcat

57061 tggaataaag atggagatca tctctaacac agtttctaag gtggtgaaaa tataatatca

57121 taataaatct aactgttctt ttcctctgca tcaaataatc ttattgtaat tttatatcaa

57181 cggaattcct ttatgttgac ctaagttttc cagatgacta ttgggacaga attttataaa

57241 tagctttgga ttttgtgcag ctcttttaga tgtattgtgc ttattttaaa aggttgtggg

57301 gggcaattta catatccatt ggttgaatgc ataaatcgac ttagttatgc attttctgag

57361 ctctgttacc ttggtaaaga atattttaca gtttgtacca gtctaccttg agcctaccct

57421 cattaaaaca ttttaaaatc cttccagaca tacatgcaga aaactgctag gaacctaggg

57481 gactgatgta cctcttaaca taaggccaat ttcaggggaa actacagaaa gagggttcag

57541 agacaaaatg gaacattctc tttgcctctc tatgataagg aaaaaattat gatttacacc

57601 tgtcagatca taaaaaagaa aaatacgcta atacccactt ttctcatttt ttttaccagc

57661 ttagtttaag tatataatct atggcttact taagcttaac cgctaagagc attttaaaat

57721 tgataaatac atttatcacc tgcactgtag gaatgaaatt aatctaggaa ttttcaaggt

57781 tgtgggtttt gctggtttgt ttatttttta ttttctaacc attgcattta cctaatgctg

57841 tagtgaaact ccttgggttt cagttgagga cgttgctaaa gctcaccatg cccttatttc

57901 tctaggtggc caccaaatat gaagtgagtg tctatgctct taaggacact ttgacaagca

57961 gaccagctca gggagttgtc accactctgg agagtaagta acaaaatgtc ttcatatgga

58021 caaaccttct gtatagacaa aaattaaaga atggtaaatc agtggggttc agtggctcat

58081 gtctaaaatc caagcacttt gggaagctga ggcgggagcg tcacttgagg ccaggagttt

58141 gagacctacc cgggcaaata gcaaggccct gtctcttaaa aaaaataaaa taaataaaat

58201 aaataatttt ttagatttat atgttaacag tggaatgagt cctaatttga aaatcaattt

58261 gattgccttt ttgacgcatg actgtcatct tttatactcc ttcagaaagg ggtctactga

58321 cccataaaat ggaatcactt cataagctta taatgttgat attatggact atgactgaca

58381 tctagtttat gctctacttg ttagaatttg ttttcataga gctaagcttg gggagacccc

58441 actggcttct gctatatctt aacaatgcat attaggccat tcttgcatta ctataaagaa

58501 ataccggaga ctgggtaatt tctaaagaaa agaggcttaa ttggcccaca ggccagcagg

58561 ctttacagga agcatggtgc tggtatcttc ttggcttcta gggaggcctt gggaagctta

58621 ctcatggtgg aaggccaagg gggagcaggc acatcacatg gctgtggcaa aagcaagacc

58681 gagagagaga gagagttggg gggggaggac cttatacatt taaatgaccc agtctcttga

58741 gaactcactg tcataaagag ggcaccaagc cacaagggat ctgcccccat gatccaaaca

58801 cctctcacca ggccccacct ccagcattgg agattacaac tcaacagaga tttggacagg

58861 gacaaatatc caaattatat cacagcacag taaccattgg accaaatcag gcttagattc

58921 tagtcttctg ttatatcaat accttgatgt atgccttttc aaaagtcagg taaagtgtca

58981 aagttttatc atttataaaa gagggatggc attgtacctg ttgagagaaa atacaaaata

59041 cttgccgtaa tattagacac acacacacac acacacacac acacactctc tctctctctc

59101 acacacacat acacacacac acacacaaaa ttgttagctg gccatgttat tgtaactcct

59161 accacacata tttttacatt ataatacatt aataatttta atatttattg aagtatttgt

59221 agatactata aagccagccc tgggaaccac tggtagtatc tataaagctt ttcagctctt

59281 caaaataaaa tgtctgagag gtagatattt tcctattttc taattacagt tgacctttct

59341 ctctgaatgc caaaggagat aatctacaca ttactagtta tatatttctt gaaatggatg

59401 aatttgatat ataccaagga aacgttttaa aataccaaaa ctttacatgg atgagccaag

59461 caggcactaa tctctagcta tgctcctgtg cagatgtcag cccaccaaga agggctcgtg

59521 tgacagatgc tactgagacc accatcacca ttagctggag aaccaagact gagacgatca

59581 ctggcttcca agttgatgcc gttccagcca atggccagac tccaatccag agaaccatca

59641 agccagatgt cagaagctac accatcacag gtcagggaac tcattgcact aaccacattt

59701 gttaacaaat acccacaatg taaacgggct tattaactgt tctacgactg acactgataa

59761 aatttatttt cagtgttatc atcataaccc agttttagaa cgttattttc atgctatgat

59821 cagaaatagt tttgtccttt gaatgcctga ttttgtgtaa tatttgtcat ggaaattgcg

59881 taagtgtcaa tcaacaagtt tgatcttcca tcattgtgcc ctttcttatt taaaaaattg

59941 taacataagg tttaaaacta aaagaaataa aaaacagtga tgtatagatc ttagcattaa

60001 aaagcatagt taatataaaa gtaaacaata ccacttaata aaggccaaaa ttgtaaccga

60061 agaatattca atatctgagg tcttttttag ctttttaaaa ttgtgattcc aaggctcaac

60121 tattgaccat ctgattacgg taaagagaaa acctcaataa gtggctgacc cccattctgc

60181 aagagggcct cttccaacat agcatttttg cattccagaa tttactttac cagtgtcctt

60241 gtctgtatca gtgattcact ttcgagatat gtttcttgtt aacagttaac atccatagca

60301 tgctctactt tactgttcaa atgtggacca ctttggtagt ctatataaat atgggatgat

60361 agaagaaccc agaaaaattg caggctagct tgagaattct cctagtaaaa agcaagaact

60421 gttaaaaatc atctcttctc aaatcccagg tttacaacca ggcactgact acaagatcta

60481 cctgtacacc ttgaatgaca atgctcggag ctcccctgtg gtcatcgacg cctccactgg

60541 taactatacc ttctactgag gaaatgccat tgacttgtat gcaatcagtt tcatgaactc

60601 aaaaaacaaa tgtgaggcgt atatttttgt attatagatt ccagagaatc ttgtttccgg

60661 tttacagtat tctcagattc ttttaagtgt gtttagaacg gctcgggaga aaagtgtggg

60721 agtaattttc ttggttattt gccttcttag agacttaatt ttgttttctt tcagccattg

60781 atgcaccatc caacctgcgt ttcctggcca ccacacccaa ttccttgctg gtatcatggc

60841 agccgccacg tgccaggatt accggctaca tcatcaagta tgagaagcct gggtctcctc

60901 ccagagaagt ggtccctcgg ccccgccctg gtgtcacaga ggctactatt actggtattg

60961 ctgcttccat gctgtcattt tccttcttac tacctaggac acatgaagtc cttagcaaac

61021 tcccacagcg tctttgatac tgtgtcatga gaatgcgaaa ctctgttcct gataacctca

61081 aaaagcattc tctgtgtagg agtggtagag cctaatacat cccaaaaggc atgagtgaag

61141 gaaaatgcaa tttcaagact gtactaatgg catgactaga ctcatgtttt cctttcgctg

61201 caagtttgcc agatacctgt caattcagtc ctggagaaag atatttttca aagcatacta

61261 gctgattgtg attctgtcat tacactcagc tctctataga tatggcaatc ttgcaggact

61321 tgccagtgca ccacctgcca ttgaccttgt tgaccactat cacaggatag gtcttgaggc

61381 agagcagtcc caaccaccca cattggaaga atgcctggaa tggggaataa gagttgtcta

61441 ccttggtggg aaagactata agcctctagt attatttttg cccaagagat gaaatattta

61501 aactatctgt attagtctgt tttcatactg ctataaagag gtttaattga ctcacagttc

61561 cgcatggctg gggaggcctc agaaaatgta caatcatggt ggaaggaaaa gcaggcatgt

61621 cttacatggc agcaggagag agaagcacac aggaggaact tccagatact tacaaaacta

61681 tcagatcttg tgagaactca ctatcatgag aacagcctgg gggaaccacc ccatgatcca

61741 atcacctcct ctcctcaata catggggatt acaattccag atgagatttg gatgcagaca

61801 cagagccaaa ccatatcagt ctgtatagag tatcacctgg actttaaaat tcccacagaa

61861 catacagaca ttagaaggag acactggctt tttagaattg gggggaacag gaaaatagaa

61921 gcagacatga gaggaattga actagacact tcccacagag gctgcacaaa cactggccaa

61981 tctctcctac ccttcacttg cctttagttt cacttttcat tgatctgcca ctgaggactg

62041 cttggttatt aggcctaagt agattcatgt atataatctg caggactctc tttcaaattt

62101 atattccagt ggtggtatat gatgctgata gattttctta aattcaaaaa ggcaaataag

62161 accacgttaa aagaataccc tggaaaggcc aggcgcggtg gctcacgcct gtaatcccag

62221 cactttggga ggccgaggca ggcagatcac aaggtcagga gatcgagacc atcctggcta

62281 acacggtgaa accccatctc tactaaaaat acaaaaacaa aattagccag gcgtggtgat

62341 gggtgcctgt agtcccagct actcgggtgg ctgaggcagg agaatggctg aacctgggaa

62401 gcggagcttg cagtgagccg agatagcacc actgcactcc agcctgggtg acagagccag

62461 actccatctc aaaaaaaaaa aaaaaaaaaa aaaaaagaat accctggaaa agttagccaa

62521 aaaatgtcta ttcaggcgtc agatatgata gtaagataat tagtttgcga tgcggacttt

62581 atatgcagga tatttgggtg tttatggagg aaaagtgaag tcgattttac cttcaagagg

62641 ccaacagcca gctggagagg aagtgcctgc tcccagtagc gtctgctggt gagaccgact

62701 tccacttgac tagctgagcc cattgacata atgtgatggt tctattctcc cttcaggcct

62761 ggaaccggga accgaatata caatttatgt cattgccctg aagaataatc agaagagcga

62821 gcccctgatt ggaaggaaaa agacaggtaa gagtatcttg caggtaacaa ggagaaagat

62881 aggacaaaac taataacaaa tgagcaatct tgcaatatga aaaggttctc catgttttga

62941 tgcatttctt gtgatttttt ttatctaaca gcatagtgta tatattgtat tctttaatag

63001 gagaaataat ttaacatgca ctgcagagtt tggttttatt ttttttcttt actacagcca

63061 ctcaatataa agccttgtta ttcacctttt aaaaattcaa acaagatgtt aaaatgtaaa

63121 agaagagcta tcattgctct tcttttatac cttctgttga attttaaaat gtttcctttt

63181 ttaaaggagg gagagaaacc tctcacattt atctttattt ggtttctaca acttagagct

63241 aaataatgtc ttacttttgc atccagtttc agttaatttc aagaaaatgt gtattcctga

63301 tataggaaaa tttcaaaaat gaacatgtgt gttttatcta tttttaccat ttcaaaccat

63361 gaaaaactgt tgagccaaac ctctgtaatt ctcatactta tgacactgat atgattagtc

63421 tggattctac ttcctacaac ttgcttctca aatttaaaaa agaaagagag aaagagaaag

63481 actgcacatt tcagttccat taggtctaat ttgagcagag gcagcttcta cggggctcag

63541 cggtttaaag ctgtgtgtat gataaattca tactaacact tttttctttc taaactataa

63601 agaaaccttt gagaaaaatc ctaaagattt ctttctggaa aaagtgtttt gtgatctcag

63661 aactgctcat tttctggtgg cttttatcaa attgatgaac agtcattgtt gcctgaatcg

63721 attattatca ttgctgctac ttcctggagc ttaatgcgct ttgctttttt ggctctaacc

63781 tctctcggct agacgagctt ccccaactgg taacccttcc acaccccaat cttcatggac

63841 cagagatctt ggatgttcct tccacagttc aaaagacccc tttcgtcacc caccctgggt

63901 atgacactgg aaatggtatt cagcttcctg gcacttctgg tcagcaaccc agtgttgggc

63961 aacaaatgat ctttgaggaa catggtttta ggcggaccac accgcccaca acggccaccc

64021 ccataaggca taggccaaga ccatacccgc cgaatgtagg tgaggaaatc caaattggtc

64081 acatccccag ggaagatgta gactatcacc tgtacccaca cggtccggga ctcaatccaa

64141 atgcctctac aggacaagaa gctctctctc agacaaccat ctcatgggcc ccattccagg

64201 acacttctga gtacatcatt tcatgtcatc ctgttggcac tgatgaagaa cccttacagg

64261 taattaattg ttctcttcac ttctcatggg gcagcacaga aaggaataag ttaggtaact

64321 gaagtgacca gccctcgaat aaaaagtggc ttcatggccg ggtgtgatgg ctcacgcctg

64381 taatcccagc actttgggag gccgaggcag gtggatcatt tgaggttagg agttcaagac

64441 cagcctggcc aacatggtga aacctcgtct cttgaaaaaa aaaaaaaaaa aaaagtggct

64501 ccacttttag aacctcttag aagatggcac atttaagccc tgcttttttt tttttttaaa

64561 tcccaatatg gctctacttt ggaggacata ccagagagtc actagctttt atttcataga

64621 gaaaatgaaa ctatttctct tattctcaca catttgaggt tcctttttga gtaagataga

64681 tgattctaga aaagaaagat attctacctg aatttccatt tgtgtgcaga agtctaaaac

64741 actaccttta cgatttgtcc ttgaagaacc ccactatcta caacatatct aaagaaaaaa

64801 aaaaacaggc gaagctgtgc atagcagctg ataagtgatt gattctctaa aacgtatatt

64861 atttaatttg tgttgacagt atccattttt ttttttcccc gagatggagt cttgctctat

64921 ggccctggct ggagtgcagt ggcgtgatct cggctcactg caacctctgc ctcccaggtt

64981 caagcaattc tcctgcctca gcctcccaaa tagctgggat tacaggcatg tgccaccgca

65041 cccagctaat ttttgtattt ttagtagaga cggggtttca cgatgttggc caggatggtc

65101 tcgatctcct gacctcgtga tccgcccgcc ttggcctccc aaagtgctgg gactacaagc

65161 atgagccacc cactacaccc ggcccactga cagtatcaat ttttattgtg ttgttacttt

65221 tagaaagtgg cagaatttaa aaactgacaa cactgtagga aatttatgag cttagaaaca

65281 tgagtttgag gatttgccca actgttttaa ggactccaca ctggggtcag atgtcacctg

65341 gaggagcatg accgtgtctc ccatatagcg cagtgtccag gttttatgtg aagcaaacat

65401 ggccagggct tccagagggc ttatgcagac ctgcgactga agcaagatca atggcaggcc

65461 gtctctagta ttgtcgaggg ctcctgttaa ctacggagca cgtaggtaga ttgttggcag

65521 gaaaatctgg caggaacgat ggcccctatc cttgttccat ttctctcctc agctggttag

65581 gaccactata ccctccctct tttttttttt tttgtttttt gttttttgtc ttcctttgct

65641 ttgtttaaac agtgagggtt attggtaaga ggagagcccg tgtcattcct cactataatg

65701 ctttctctct gctttggatg taccgataat tgcagttcag ggttcctgga acttctacca

65761 gtgccactct gacaggcctc accagaggtg ccacctacaa cgtcatagtg gaggcactga

65821 aagaccagca gaggcataag gttcgggaag aggttgttac cgtgggcaac tctggtatgt

65881 aaacacgtac tatttagaca caggctcccc tctgctgtac accagagatg ggcttttctg

65941 ttgactgtac ctttgttgcc attgtctttt tatctttggg atttaatgca acacatcaac

66001 atgaaataaa tgagcaactt tatattaaat taatctctcc cccacctcct gccatatcct

66061 gttgtcttca caaaatgcat acgtaattga cagactctca aatggtgata tgattataga

66121 tctggaaggg atttcaaata ttatttagta caacactctg agtccttact tctgagtatc

66181 tgagttgata tagggcacag gtttcctgat gtcttttccc agaccctctc catctcacca

66241 tgctgctgtc ctcttgagtg attaaatact gaaacgatta cctataaaga aaatacccct

66301 tctgcagaca tggggacagt tggcttttgc tcctgatata aaatgctacc aacattgtgc

66361 atttctgtct gcagagaatg ttattccaat gttatttcca tttttttcca atgttatttc

66421 catttttttt tctgactata caggttaaaa gcttctatag aggttaaaag atcattaact

66481 cttctttgta gcacctggga aatcctttta aatcaatagc gtgccacctg gctgctcaat

66541 ttgcagcagc tgaaaattca ccaaggcaca tgagataggg gataatcaaa accgtgaatc

66601 cccaatcttc caacaggaga gttctctact ccacaccaac agagagtgct aagtcctgtc

66661 tatgccaagt gacagatttt attcctaagg ccagttgttt aattttagcc ccttccctca

66721 tctgatagaa gactgtgcta ctttacatgt ataaattcct gtgaattaag cagttgagca

66781 tttggctgga gagaggttgg gaggagatta ttttgtgttt gttgtattac atatccacag

66841 taatgcttat ctttgccttt tgtggtttta ctagtagaat gccacgtgaa cagaattttc

66901 aagagcaaaa aggtctttgt gcttttctaa gtcatttttt tttttttttt ttaaagattc

66961 catctcttta actttagtta ggatggaatt tgaactcctg gctcttttga gtatagaaac

67021 ccctagtaac aatttaagtt ccttccattt ttcttttaaa ctccttattc ccagcagcag

67081 tattctacat tctaaccagg ttctcccagc tttgagacgt ctcagactta ccagttctcc

67141 aaaacgctat tttctttaag ggtgacacct tttaaaaatt aggcacctca aatatctact

67201 gcttttgagc ttttgagttt tgcactgtaa aaagaaaaat acacagtggg attttaagtc

67261 aaattagttt atctaatttt tagggaataa tttgaagcat gctttgtttg catagatttt

67321 tttaaaataa gcttttccaa atcataaaga gataagatct taggtaacat gaagagactc

67381 ccttacttat tcctaaatca tctatattcc aagggcattt tcttatttgg aacagttgac

67441 ctcactgata aagctgtctc accactataa taacaatgtc caaaatctag gctttctgca

67501 ctattatgca aaaattacaa taataaaagt gaaaattaca ttataatggt atattaaaat

67561 gctaagactt ttgcattata agcaaaagac agcctttaat aattattctt tatttagtga

67621 acattttcta agtcttggaa aagggtcaat gttttgaatt catggcctta tataatcttc

67681 acaagattcc ccaggaggta tagatatttt tattattacg ctagtattgc agatgaggga

67741 agcaaggcag agtggtatta aatagctggc ccaaggtcac tcaggtacca atggagaggc

67801 atcattagtc tttgcatccc actaaagttc tccactagct tcaattgcct caagatctgt

67861 tccatgttct atgaagtagt ttcaacagaa atggcaatta tcttagaagc aagggaaaaa

67921 taaaagatgg gcttcctgtc gggtgcctgt gacaggtgtc acatctaacc atggtttttt

67981 agagcagtta atgccttgat agaacagatg aatgcctctt aatcctcctg gaattcttgt

68041 tttagattaa gtcattgtat acagtcattc gattttcttc ttatggtcca aatcgattaa

68101 taagatgtct ctttttgctt tttcttcctt ttcttcatag tcaacgaagg cttgaaccaa

68161 cctacggatg actcgtgctt tgacccctac acagtttccc attatgccgt tggagatgag

68221 tgggaacgaa tgtctgaatc aggctttaaa ctgttgtgcc agtgcttagg ctttggaagt

68281 ggtcatttca gatgtgattc atctagtgag tagttgcttt gtccatccac ttccgtgttt

68341 gtctcctcaa gttccatgca tgcactcatg tgccaaggaa gcatgtttgg aagacacagg

68401 ttcttccaaa catgaagcaa acaagagaat actgtttgac tcgaagtaat attttgcatc

68461 atagaaaaat gatgggaaat tttacttgtt ggacattgct tcatttcaag ggttgtatgc

68521 caatacaact attaattaca cataagatta tggtgctaat ttgatttttg aaattttctg

68581 tgaaaacaaa tggataaaga cttttggaac caggtctatt taagagtatt agagacacag

68641 aaaaacctca aatctctttt aatcttcagt gttgaatgag atcagaggtg aacatttaga

68701 ctcaaaaaca gcctccttca acataaacca aacatgcaca tatcatagta cccatgcaca

68761 cacttttgcg tcacacacat agcccaggta gcttgaacgt tgctagaaat atgaaagaaa

68821 aaacagataa tctgctttta gatcattaaa aatcaacttg aattgataaa tgtttgattt

68881 tcaaattcta atacgtttta attttcaaat tttttaagtt aaaatgtgcc taggaaatat

68941 ctattatgct ttgagattag gattagaatt tataaacctt tcatttattc tttgtgttta

69001 ggagatgtga tgattattga caattggttc atttttatag gtgttgaccg ttatgcctat

69061 aaataagcct cctatagaca tacagaaatc atatcctgtg gaattagaat ataagacttg

69121 gtaaaagaga ttttcaaagt attttactta acttgtatac ttgaaatcat ttaatccaga

69181 ctgaagttgt aaaagccagc cagtgttttc aatatagact tccatgtttg accatctgaa

69241 aatgaaaaac actaaaaaca tcacatgctg tttaggagct ggaaatttta atatttgact

69301 tcaagtagat ggtttttaac tcctgaaatc gaactacgtt taagtttgta tgtttattac

69361 ctgtttgagc acttaggtgc aattgtggga gcggggatgt caagttcatt tatgtgactc

69421 tttggctcaa cttacataat ctttgttttg atatcacagt tgtctaatta ttttactttg

69481 tagcttaagg caggctgaat tgttgataaa atggaaaaag tagtatattg ttatataagc

69541 ttctgaggtg tgttttgttg tataagccct ggaggttaaa aagtcatccc ttatgtatag

69601 tagttaaagg cataaaactg tgacttttag atattccaca gaaccagact tatttgatgt

69661 ggataataac caatgattta gcattttgtt tgcttttgtt ttattttatc cgggttcatt

69721 ttttactctt cccatgtaca tgaaacaggt ggtggcgtgt agagatcagc tgatccttgt

69781 tttatggtta attgaactac tttgtatcca gggtttctgc aaatccaaaa gtgatttttc

69841 atctaggatc tattcctaac agtctactcc aatcccactt tagttttcca caattttaaa

69901 tcttaatagt gagaattcaa atgaaagtca tttcatttga ctattctgat gacatgattg

69961 tggcagaata aattgggtct taaaatgccc tagaaaatgg taaatgataa aaaataatat

70021 tttaaaattc aaccaaagaa atggcccatt ggccaggtgt ggtggctcac acctgtaatc

70081 ccagcacttt tggaggctga ggcgggtgga tcacctgagc tcacgagttt gagaccagcc

70141 tacccaacat ggtaaaaccc catctctaca aaaaatacaa aaaaaaaaaa aaaaaaaaaa

70201 atagcactgt ggggagtgcc tgtaatccca gctactcagg aggctgaggc aggataactg

70261 catgaaccca ggagatggag gttacagtga gccgagattg caccacttca ctccagtctg

70321 ggcgacagag aaagactttg tctcaaaaaa aaaaaaaaaa ataaaaagta aataaataaa

70381 taaaataaat ggcccattat aggggttttt atctttaact tgctattttt ccagatcatg

70441 gttctgaaga ccctgtgaca cgtcccagtt cacctactgt cttgtgagtc agaatataca

70501 aataactttt tggtcctgac tttccccacc cctacaggat ggtgccatga caatggtgtg

70561 aactacaaga ttggagagaa gtgggaccgt cagggagaaa atggccagat gatgagctgc

70621 acatgtcttg ggaacggaaa aggagaattc aagtgtgacc ctcgtatgtc atcacagatc

70681 atttttagtg ccttattaag cattctcact ttcattatca ggctgtaact ctcattcaca

70741 gaaatgattg gagactttag gtctccttga ggagtgaaca gtgggtttct taatcttttg

70801 atttgggaaa gtggagacaa gcttcaaaaa tgagtcatga tttaatgtta ttacaggaca

70861 ctttagcact tgtccaacct gagtattttg accattatct gcagtaaaat gctacaaaga

70921 agctttattg gtctgtagat tcaactttta aaatatgatt tccatcttcc cgttggaccc

70981 tttccagtgt attaggtcta atttttggaa gtgccaccct aagatctgta tagcagtact

71041 gctcttaggg atgattcaca taaatatgtg gtgtttgcgc tgtgatgata caaatttagg

71101 acagaaatag aacccacccc tagatcaagt ctgcagtatt gttctcagct tatgcgtgca

71161 tctgtcttgt gtctatatgc agatgaggca acgtgttatg atgatgggaa gacataccac

71221 gtaggagaac agtggcagaa ggaatatctc ggtgccattt gctcctgcac atgctttgga

71281 ggccagcggg taagactgga tgtgccaggc tccctacaag ttagataaga taaagggtgg

71341 gctcctgcaa ggatgtgtcg tacacacagg aggggcagag acccttcgga agtattaaaa

71401 taccacattt cctgttggca tacaactgct gacatagagc tctagagcag ctctatgtct

71461 accttacatg ccattcattc tttctattac tcttagtaga aagaatgaat gaatggcatg

71521 tagagtacca aaaacacaag tcttgagtca ttcttaatag caacacctgt catttatatg

71581 atgttagaat cattttccta agctccctag catgtcagag atactattta cactgaaaaa

71641 tagtgaagca gagatactat tcaaattaat tagtggtaaa tagaatgtgt ttcatttcag

71701 ccggttctcc ccatcctggg cagcctgaga ccctcccctc ccctactatt ctcaggctgc

71761 ttctattttt cagcaaagtg ttaagtgcag tgtagctcta ggcctccaac tccattctga

71821 tggacaggtg tcccatggca acgttgttaa atattttgaa taatatctca gatgtaagaa

71881 aatgccactt cttttaacct ctctcttgat tcagaacaga tgcttgttat aggtctagca

71941 ctgtgctaag tagtatagga aaaacagagg aaatgagaaa tggcttggct cttaatgata

72001 tagttgaaga tgttaaatta gcatacattt caaagtcaag ctaattaagt tctaagtggg

72061 tctgacaaat acagttctgg gtaggctgga attagcaaga aagagaagca tgaactggct

72121 gaggtttacg atgactaagg tttagttggg aggggagaaa gcagagagac acaccccctg

72181 ggatagaaag gagctggccc aggtgggctt tggtgagcca accctctgcc tgctgtcttc

72241 tggtaagaaa atagatggga agaagtggct tatggagggc cttggcaacc cattatttaa

72301 gccagtactt ctcaaccatt tctaaaatat gcccagtata acaaaaaata ataagccttt

72361 ctctaatatg atttcaaatt tcaaaatgaa attatgtgta actcaaaagc aatggaatgt

72421 gacagccctt tgttttcaac gaagacatgc cctcccagca actccccaaa tcctggtggg

72481 tgaggggcat gcttcacact caagggtgag actcattggt taatgccaaa tgcattaacc

72541 aattacaggt atccaagatg caaagaaaca tgatggaaaa tagtctttgg gaaaattaat

72601 ctggcagcag gggtgttcag gggcctgtct tggctccacc aggggcagcc catggaaact

72661 actatgatct tgtttcaccc ccagtgatta catggggagg gaggtgctcc caattctgat

72721 ggaggagaat tggagattgg aatttagatt gaattcagta tctctctctg tctctctctc

72781 tctctctctc ccattaacac ttacaacgac tgtgattgta tgaccttaga actcagtcat

72841 tctggtatga aattgtgtga tggagaatga atttgctggg aagttgattt tggtctcact

72901 tcagcatctt ctcattattt atgcacatga aacctttcat gtgcgacact tattctattc

72961 tcaagtgcta aatgaaacat ttaagacagg agtggaaact gttcactttc tcatatgaaa

73021 gcaagattca gtgattctgt aaggaggtag tcactggtat tgtgttaggt attaaggggc

73081 atatgtgctt aaacagagaa atatgtctaa aatatttaaa ttctaatata aaaaagaaag

73141 tgactgtatt atttagggct gcattttagt tgtaagaaaa aagtccaact caagcaaaaa

73201 tggcccacac aatggaacag tcccaggacc caccggcttc aggggctgct ccagcaatgg

73261 cgcccggact ccctcttgct ccgcgtgcct tcccatgcac tggcttcgtg cttcagcggg

73321 gtctctgctg atggtgccat tgatgactga cctccatgag cttgctttac cccctgccag

73381 cttaagaaca gtagtgaaag agaacatgtg tgtcctccca tttccagtaa aaacttcagg

73441 caggagcctc actggctcag cttggtcccg tttccatctc ccatgccatc tccggccagg

73501 tgacaggcta ccatgtcact gcctagggaa gtttaggaag agagtggcaa agtggtgcat

73561 tagaaagaac atggccaggt caccccacct cctgggcggc aggcccaact ccaccagtgg

73621 tccactgtgt gacttccctg ctccctctaa gcaagtcact cctctcctct gggtctctgt

73681 ttccttacct ataaaatgag aacgtttctt catgtgatct caagtccctt ttaaaatcgc

73741 taggattctt tgaaaacctt ttctatcatc tagtgcagag aacttgttga ggaagttggg

73801 attggaatga gcctcagcag atgggcaagg tttgaatagg aagagaagag acatttcagg

73861 agaaagaaac aacatagaga gacagatgta ggtataagat atggtaataa gccaaaatgt

73921 attaagagtt ataaatgcat gaaatcatca tcaaagcttg cttagtgatt aactgcttat

73981 attttgccag tgcatatgat gtgacatttt tctttaactc aaacactaaa ttacgatgtc

74041 ctcaggttat cataaacccc atttgacttc atgcctctac tctctcaggg ctggcgctgt

74101 gacaactgcc gcagacctgg gggtgaaccc agtcccgaag gcactactgg ccagtcctac

74161 aaccagtatt ctcagagata ccatcagaga acaaacactg taagtgcatt agcagcacaa

74221 gtgtgttccc tcatactaga cagtctcttt ctacaggtat ctttcttcag aatgaaccaa

74281 gtgttttaat taattaaaaa aaaaaacaac tcataaatga cttaagtgaa acactgtatt

74341 ccataatata gtttaagtta taatttatgt aactcttgaa catctcctat tgcccagtat

74401 gctgctaggt tcttgaaact aggaagaaat attatcctat ctataagcag ctgtcatgag

74461 tccccacctc cccgcatttt tttttctgta cactttacag tatttgccac taattttttt

74521 ttccttcttc ctttttaaca gaatgttaat tgcccaattg agtgcttcat gcctttagat

74581 gtacaggctg acagagaaga ttcccgagag taaatcatct ttccaatcca gaggaacaag

74641 catgtctctc tgccaagatc catctaaact ggagtgatgt tagcagaccc agcttagagt

74701 tcttctttct ttcttaagcc ctttgctctg gaggaagttc tccagcttca gctcaactca

74761 cagcttctcc aagcatcacc ctgggagttt cctgagggtt ttctcataaa tgagggctgc

74821 acattgcctg ttctgcttcg aagtattcaa taccgctcag tattttaaat gaagtgattc

74881 taagatttgg tttgggatca ataggaaagc atatgcagcc aaccaagatg caaatgtttt

74941 gaaatgatat gaccaaaatt ttaagtagga aagtcaccca aacacttctg ctttcactta

75001 agtgtctggc ccgcaatact gtaggaacaa gcatgatctt gttactgtga tattttaaat

75061 atccacagta ctcacttttt ccaaatgatc ctagtaattg cctagaaata tctttctctt

75121 acctgttatt tatcaatttt tcccagtatt tttatacgga aaaaattgta ttgaaaacac

75181 ttagtatgca gttgataaga ggaatttggt ataattatgg tgggtgatta ttttttatac

75241 tgtatgtgcc aaagctttac tactgtggaa agacaactgt tttaataaaa gatttacatt

75301 ccacaacttg aagttcatct atttgatata agacaccttc gggggaaata attcctgtga

75361 atattctttt tcaattcagc aaacatttga aaatctatga tgtgcaagtc taattgttga

75421 tttcagtaca agattttcta aatcagttgc tacaaaaact gattggtttt tgtcacttca

75481 tctcttcact aatggagata gctttacact ttctgcttta atagatttaa gtggacccca

75541 atatttatta aaattgctag tttaccgttc agaagtataa tagaaataat ctttagttgc

75601 tcttttctaa ccattgtaat tcttcccttc ttccctccac ctttccttca ttgaataaac

75661 ctctgttcaa agagattgcc tgcaagggaa ataaaaatga ctaagatatt aaaagtattt

75721 gaatagta

**hsa_circ_0058124_SPLICED**

>hsa_circ_0058124|NM_212482|FN1

GCAACACCGTGACAGGAGAGACGACTCCCTTTTCTCCTCTTGTGGCCACTTCTGAATCTGTGACCGAAATCACAGCCAGTAGCTTTGTGGTCTCCTGGGTCTCAGCTTCCGACACCGTGTCGGGATTCCGGGTGGAATATGAGCTGAGTGAGGAGGGAGATGAGCCACAGTACCTGGATCTTCCAAGCACAGCCACTTCTGTGAACATCCCTGACCTGCTTCCTGGCCGAAAATACATTGTAAATGTCTATCAGATATCTGAGGATGGGGAGCAGAGTTTGATCCTGTCTACTTCACAAACAACAGCGCCTGATGCCCCTCCTGACACGACTGTGGACCAAGTTGATGACACCTCAATTGTTGTTCGCTGGAGCAGACCCCAGGCTCCCATCACAGGGTACAGAATAGTCTATTCGCCATCAGTAGAAGGTAGCAGCACAGAACTCAACCTTCCTGAAACTGCAAACTCCGTCACCCTCAGTGACTTGCAACCTGGTGTTCAGTATAACATCACTATCTATGCTGTGGAAGAAAATCAAGAAAGTACACCTGTTGTCATTCAACAAGAAACCACTGGCACCCCACGCTCAGATACAGTGCCCTCTCCCAGGGACCTGCAGTTTGTGGAAGTGACAGACGTGAAGGTCACCATCATGTGGACACCGCCTGAGAGTGCAGTGACCGGCTACCGTGTGGATGTGATCCCCGTCAACCTGCCTGGCGAGCACGGGCAGAGGCTGCCCATCAGCAGGAACACCTTTGCAGAAGTCACCGGGCTGTCCCCTGGGGTCACCTATTACTTCAAAGTCTTTGCAGTGAGCCATGGGAGGGAGAGCAAGCCTCTGACTGCTCAACAGACAACCA
